# Independent-channels models of temporal-order judgment revisited: A model comparison

**DOI:** 10.3758/s13414-024-02915-5

**Published:** 2024-08-06

**Authors:** Paul Kelber, Rolf Ulrich

**Affiliations:** Department of Psychology, University of Tübingen, Schleichstraße 4, Tübingen, 72076 Germany

**Keywords:** Temporal-order judgment, Simultaneity judgment, Independent-channels models, Psychometric functions, Cognitive modeling

## Abstract

The perception of temporal order or simultaneity of stimuli is almost always explained in terms of independent-channels models, such as perceptual-moment, triggered-moment, and attention-switching models. Independent-channels models generally posit that stimuli are processed in separate peripheral channels and that their arrival-time difference at a central location is translated into an internal state of order (simultaneity) if it reaches (misses) a certain threshold. Non-monotonic and non-parallel psychometric functions in a ternary-response task provided critical evidence against a wide range of independent-channels models. However, two independent-channels models have been introduced in the last decades that can account for such shapes by considering misreports of internal states (response-error model) or by assuming that simultaneity and order judgments rely on distinct sensory and decisional processes (two-stage model). Based on previous ideas, we also consider a two-threshold model, according to which the same arrival-time difference may need to reach a higher threshold for order detection than for successiveness detection. All three models were fitted to various data sets collected over a period of more than a century. The two-threshold model provided the best balance between goodness of fit and parsimony. This preference for the two-threshold model over the two-stage model and the response-error model aligns well with several lines of evidence from cognitive modeling, psychophysics, mental chronometry, and psychophysiology. We conclude that the seemingly deviant shapes of psychometric functions can be explained within the framework of independent-channels models in a simpler way than previously assumed.

**Supplementary Information:**

The online version contains supplementary material available at 10.3758/s13414-024-02915-5.

## Introduction

A fundamental and classical question in psychophysics is how humans perceive temporal relations between different stimuli. This question arises for stimuli encountered in the same sensory modality (e.g., two lights) as well as for stimuli encountered in different sensory modalities (e.g., one light and one sound). Although accurate temporal integration is essential for many cognitive operations such as speech recognition and motor coordination (see e.g., Buonomano & Karmarkar, 2002; Lashley, 1951), the perceived and the actual temporal relation of physical events often mismatch (e.g., Dennett & Kinsbourne, 1992; Shore et al., 2002; Stone, 1926; Zampini et al., 2003). Astronomers of the 18$^{\text {th}}$ and 19$^{\text {th}}$ century already became aware of this when they noticed considerable discrepancies between their judgments about the time of star transits (Mollon & Perkins, 1996). The astronomer Friedrich Bessel thus established the concept of the “personal equation” to take into account the dependence of temporal judgments on the observer’s perceptual processes. These events constituted a starting point of experimental psychology (Boring, 1950), as the research of the astronomers was picked up by pioneering psychologists like Wilhelm Wundt. Since then, psychologists have been devoted to related topics, such as whether an attended stimulus gains faster access to consciousness compared to an unattended stimulus (“prior entry”; Titchener, 1908). In fact, a wealth of research suggests that attended stimuli tend to be perceived earlier than concurrently presented, unattended stimuli (e.g., Jaśkowski, 1993; Schneider & Bavelier, 2003; Sternberg et al., 1971; Shore et al., 2001; Spence et al., 2001; Tünnermann et al., 2015).

### Judgment of temporal order and simultaneity

The two most widespread tasks used to study such phenomena are *temporal-order judgment* (TOJ) and *simultaneity judgment* (SJ) tasks.^1^ In each of these binary-response tasks, the presentation of two stimuli $S_x$ and $S_y$ (e.g., light and tone) starts at times $t_x$ and $t_y$, where the stimulus-onset difference $d = t_y - t_x$ is manipulated across trials.^2^ Since $t_y = t_x + d$, positive, zero, and negative values of *d* reflect that $S_x$ is presented first ($t_x < t_y$), that $S_x$ and $S_y$ are presented simultaneously ($t_x = t_y$), and that $S_y$ is presented first ($t_x > t_y$). In the TOJ task, subjects respond whether $S_x$ appeared first ($R_{xy}$) or whether $S_y$ appeared first ($R_{yx}$). In the SJ task, subjects respond whether $S_x$ and $S_y$ appeared simultaneous ($R_{si}$) or successive ($R_{su}$). The TOJ task yields the psychometric function $P(R_{xy}\,|\,d) = 1 - P(R_{yx}\,|\,d)$, which tends to increase with *d*. The SJ task yields the psychometric function $P(R_{si}\,|\,d) = 1 - P(R_{su}\,|\,d)$, which tends to exhibit a bell shape over values of *d*.

TOJ and SJ tasks are commonly used as tools to infer effects on perceptual latency (see e.g., Badde et al., 2020; Vroomen et al., 2004; Zampini et al., 2005). Nowadays, this usually involves fitting a somewhat arbitrary function to the data (e.g., logistic psychometric functions for TOJs, Gaussian functions for SJs), based on which certain indices such as the “point of subjective simultaneity” (PSS) are derived. A shift of the PSS is then often used as a proxy to support statements about perceptual latencies without further specifying the underlying mechanisms.

The present study takes up recent research that strives to explicitly understand the mechanisms underlying human performance in such tasks (e.g., García-Pérez & Alcalá-Quintana, 2018; Sternberg et al., 2023; Tünnermann & Scharlau, 2016, 2018a, 2021; Schneider & Bavelier, 2003; Yarrow et al., 2015, 2022, 2023). To this end, a multiple-psychometric-functions approach (Allan, 1975a; Ulrich, 1987; Sternberg et al., 1975, 2023) is used to distinguish between different versions of independent-channels models (Sternberg & Knoll, 1973) that were introduced to account for non-monotonic and non-parallel psychometric functions. More specifically, we evaluate the response-error model (García-Pérez & Alcalá-Quintana, 2012b), the two-stage model (Jaśkowski, 1991b), and a two-threshold model that is based on an idea originally considered by Sternberg et al. (1975, 2023)^3^ and more recently by García-Pérez and Alcalá-Quintana (2015b, 2018). To foreshadow, the simplest of the three models, the two-threshold model, provided the most satisfactory account.

### Independent-channels models

If explanations of performance in TOJ and SJ tasks are offered at all, they usually fall within the class of *independent-channels models* (Sternberg & Knoll, 1973). Their basic form is as follows: The stimuli $S_x$ and $S_y$ are processed in separate peripheral channels until each stimulus is registered at a central location in the brain. The arrival latencies to register $S_x$ and $S_y$ are represented by the random variables $\textbf{L}_x$ and $\textbf{L}_y$. Thus, $S_x$ is registered at arrival time $\textbf{A}_x = \textbf{L}_x + t_x$ and $S_y$ at $\textbf{A}_y = \textbf{L}_y + t_y = \textbf{L}_y + t_x + d$. A central decision mechanism determines the temporal order of $S_x$ and $S_y$ according to a decision rule that operates on the arrival-time difference $\mathbf {\Delta A} = \textbf{A}_y - \textbf{A}_x = \textbf{L}_y - \textbf{L}_x + d = \mathbf {\Delta L} + d$. This architecture of independent-channels models implies that processing in one peripheral channel ($\textbf{L}_x$) is unaffected by processing in the other peripheral channel ($\textbf{L}_y$), and vice versa. It also implies that the arrival-latency difference $\mathbf {\Delta L}$ is independent of the stimulus-onset difference *d*. Further, the decision rule generated by the central decision mechanism is assumed to be unaffected by the arrival latencies generated in the peripheral channels, and vice versa.

Sternberg and Knoll (1973) have shown that a general independent-channels model encompasses many models of timing judgment, such as perceptual-latency models (Baron, 1969; Gibbon & Rutschmann, 1969), perceptual-moment models (Gibbon & Rutschmann, 1969; Stroud, 1955), triggered-moment models (Baron, 1971; Venables, 1960), and attention-switching models (Allan & Kristofferson, 1974; Allan, 1975a; Kristofferson, 1967a, b; Schmidt & Kristofferson, 1963). All of these models are special cases of the general independent-channels model, since they result from a certain specification of the decision rule.

According to the deterministic decision rule assumed by perceptual-latency models, the stimulus that arrives first appears first. However, perceptual-latency models have been invalidated by several findings (e.g., Allan, 1975a; Heath, 1984). Unlike perceptual-latency models, other independent-channel models (perceptual-moment, triggered-moment, and attention-switching models) posit that the temporal resolution of the arrival-time comparator is limited by a central timing mechanism. Specifically, according to perceptual-moment models, stimuli are only perceived as ordered if they arrive in different “moments” (the assumed discrete building blocks of psychological time), where the timing of moments is unaffected by the central arrival of stimuli. Triggered-moment models follow perceptual-moment models, except that the first central arrival of a stimulus is assumed to initiate a new moment. Finally, according to attention-switching models, attention can only be directed to one channel at a time, and attention can only be switched from one channel to another at certain time points separated by one “time quantum”. Further, an arrival is only registered if the respective channel is attended. Thus, two stimuli are only perceived as ordered in time if the temporal order of their registrations can be determined.

Differences aside, perceptual-moment, triggered-moment, and attention-switching models hypothesize a central oscillatory process, whose period (i.e., the duration of a moment or time quantum) is formally reflected in a threshold (or criterion) *c* that the magnitude of the arrival-time difference, $|\mathbf {\Delta A}|$, must reach to perceive temporal order. Therefore, these independent-channels models may also be referred to as *threshold models* (Ulrich, 1987).

Theoretically important, Sternberg and Knoll (1973) demonstrated that psychometric functions are shaped not only by the specific assumptions about the central decision mechanism but also by the distribution of the arrival-latency difference. As a result, it is impossible to examine the central decision mechanism in isolation. Instead, empirical testing of hypotheses about the central decision mechanism requires subsidiary assumptions about the arrival-latency difference.

One common assumption is that peripheral processing times (i.e., the arrival latencies $\textbf{L}_x$ and $\textbf{L}_y$) follow an exponential distribution (e.g., Colonius & Diederich, 2004; Diederich & Colonius, 2015; García-Pérez & Alcalá-Quintana, 2012a, b; Heath, 1984). In this case, the arrival-latency difference $\mathbf {\Delta L}$ follows a Laplace (bilateral exponential) distribution. Another common assumption is that $\mathbf {\Delta L}$ follows a Gaussian (normal) distribution (e.g., Jaśkowski, 1991b; Schneider & Bavelier, 2003; Yarrow et al., 2011).

In general, it seems realistic that stimulus detection requires several successive neural pulses rather than only a single neural pulse. Therefore, one might also assume that the arrival latencies $\textbf{L}_x$ and $\textbf{L}_y$ each reflect the sum of a fixed number of independent, exponentially distributed latencies, which in turn reflect the times between two successive neural pulses. In that case, $\mathbf {\Delta L}$ corresponds to the difference of two Erlang distributions. As shown in Appendix B, this difference converges to a normal distribution with an increasing number of required neural pulses. Similarly, one might assume that $\textbf{L}_x$ and $\textbf{L}_y$ each reflect the duration of a noisy accumulation process drifting towards a single absorbing boundary (cf. Ratcliff, 1978). The first-passage times of such a Wiener process follow a Wald (inverse Gaussian) distribution (see e.g., Luce, 1991). Thus, in this case, $\mathbf {\Delta L}$ corresponds to the difference of two Wald distributions. This difference is again reasonably well approximated by the normal distribution for a range of plausible mean values of $\textbf{L}_x$ and $\textbf{L}_y$ (see Appendix B). Overall, these theoretical considerations appeal to the subsidiary assumption of a normally distributed (rather than Laplace-distributed) arrival-latency difference $\mathbf {\Delta L}$.

### Shape of psychometric functions in the ternary-response task

To empirically distinguish between competing accounts of the central decision mechanism, timing-judgment tasks that rely on more than two response alternatives and thus yield multiple psychometric functions proved to be more informative than the binary TOJ and SJ tasks (Allan, 1975a; Sternberg et al., 1975, 2023). In the simplest extension of the TOJ and the SJ task, the *ternary-response task* (Ulrich, 1987), subjects choose between the three response alternatives $R_{xy}$, $R_{si}$, and $R_{yx}$. Thus, this task yields the three psychometric functions $P(R_{xy}\,|\,d)$, $P(R_{si}\,|\,d)$, and $P(R_{yx}\,|\,d)$, which can also be represented by the two psychometric functions $P(R_{xy}\,|\,d)$ and $1 - P(R_{yx}\,|\,d) = P(R_{xy} \cup R_{si}\,|\,d) =$ $P(R_{xy}\,|\,d) + P(R_{si}\,|\,d)$.

Specific independent-channels models constrain the relationship between the two psychometric functions $P(R_{xy}\,|\,d)$ and $1 - P(R_{yx}\,|\,d)$ (Allan, 1975a; Ulrich, 1987; Sternberg et al., 1975, 2023). In particular, many independent-channels models require that $P(R_{xy}\,|\,d)$ and $1 - P(R_{yx}\,|\,d)$ are monotonically increasing functions (*monotonicity*) that can be transformed into each other solely by horizontal translation (*parallelism*). On the one hand, the functions must be monotonically increasing according to perceptual-moment, triggered-moment, and attention-switching models, because these functions are conceptualized as cumulative distribution functions in the general independent-channels model. On the other hand, the functions must be parallel according to several perceptual-moment, triggered-moment, and attention-switching models, because the distributions that are assumed to govern the observed psychometric functions are identical in shape (see Ulrich, 1987).

Contrary to these predictions, $P(R_{xy}\,|\,d)$ and $1 - P(R_{yx}\,|\,d)$ were non-monotonic for several subjects (Ulrich, 1987). Substantial non-monotonicities were again observed in multiple subsequent studies (Jaśkowski, 1991a; van Eijk et al., 2008; García-Pérez & Alcalá-Quintana, 2018). In addition, Ulrich (1987) found that the skewness (third central moment) was much higher for $1 - P(R_{yx}\,|\,d)$ than for $P(R_{xy}\,|\,d)$, as measured by a modified Spearman-Kärber method (Spearman, 1908; Kärber, 1931; Sternberg et al., 1982; Ulrich, 1987; Miller & Ulrich, 2001; Sternberg et al., 2023). This skewness relation implies that $P(R_{xy}\,|\,d)$ and $1 - P(R_{yx}\,|\,d)$ are not parallel (see also Allan, 1975a). Taken together, these results have been taken as strong evidence against several prominent independent-channels models.

### Explanations for non-monotonic and non-parallel psychometric functions

In the last decades, different independent-channels models have been developed to account for observed non-monotonic and non-parallel shapes of psychometric functions (e.g., Allan, 1975a; Ulrich, 1987; Jaśkowski, 1991a). To this end, the three independent-channels models described below elaborate on the mapping from unobservable internal (perceptual) states *I* to observable responses *R*. In other words, these models assume that there is no one-to-one relation between internal states and responses in the ternary-response task. Critically, the models differ in how many internal states they posit.

First, the *response-error model* (REM; García-Pérez & Alcalá-Quintana, 2012b) assumes that after each presentation of $S_x$ and $S_y$, one of three internal states results: (a) the state $I_{xy}$ that $S_x$ appears before $S_y$, (b) the state $I_{si}$ that $S_x$ and $S_y$ appear simultaneous, or (c) the state $I_{yx}$ that $S_y$ appears before $S_x$. Crucially, the REM allows for mistranslations of these internal states $I \in \{I_{xy}, I_{si}, I_{yx}\}$ into the responses $R \in \{R_{xy}, R_{si}, R_{yx}\}$: Each internal state *I* (e.g., $I_{xy}$) is misreported with some probability, and given a misreport of *I*, each non-corresponding response *R* (e.g., $R_{si}$ and $R_{yx}$) is provided with a certain probability. This resembles modeling approaches in visual psychophysics to account for finger errors and attentional lapses (e.g., Swanson & Birch, 1992; Wichmann & Hill, 2001).

Second, in addition to the internal states $I_{xy}$, $I_{si}$, and $I_{yx}$, the *two-stage model* (TSM; Jaśkowski, 1991b) also postulates the internal state $I_{su}$ that $S_x$ and $S_y$ appear successive as well as the internal state $I_{?}$ that no order of $S_{x}$ and $S_{y}$ is perceived. Each of these five internal states is elicited by one of two distinct processes, which are assumed to govern successiveness and order detection: The arrival-time difference in the “successiveness center” (or “simultaneity center”) is translated into an internal state of simultaneity ($I_{si}$) or successiveness ($I_{su}$). Independently of this, the distinct arrival-time difference in the “order center” is translated into one of three internal states – precedence of *x* ($I_{xy}$), precedence of *y* ($I_{yx}$), or imperceptible order ($I_{?}$). In the ternary-response task, $I_{si}$ leads to $R_{si}$ irrespective of the internal order-detection state. Moreover, $I_{su} \cap I_{xy}$ leads to $R_{xy}$, $I_{su} \cap I_{yx}$ to $R_{yx}$, and $I_{su} \cap I_{?}$ to each response with a certain guessing probability.

**Fig. 1:**
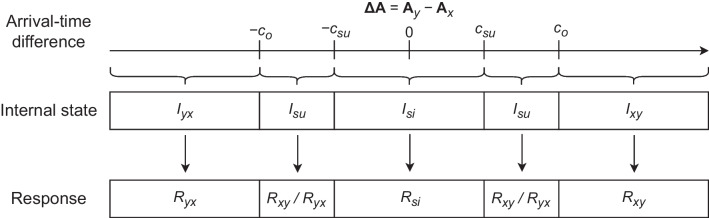
Mapping from the arrival-time difference $\mathbf {\Delta A}$ via the internal state *I* to the response *R* in the ternary-response task according to the two-threshold model (TTM). *Note.* $\textbf{A}$: arrival time; *x*: one channel (e.g., vision); *y*: other channel (e.g., audition); *c*: threshold (i.e., criterion); *su*: successiveness (or: *x* and *y* successive); *o*: order; *xy*: *x* before *y*; *si*: *x* simultaneous to *y*; *yx*: *x* after *y*

Third, the *two-threshold model* (TTM) proceeds from the assumption that successiveness detection need not suffice for order detection. Specifically, observers might simply set a higher threshold of $|\mathbf {\Delta A}|$ for order detection than for successiveness detection, which implies the possibility of detecting successiveness without detecting order.^4^ This idea was initially discussed by Sternberg et al. (1975, 2023) and more recently by García-Pérez and Alcalá-Quintana (2015b, 2018). According to the TTM, one of the four internal states $I_{xy}$, $I_{si}$, $I_{su}$, and $I_{yx}$ results depending on the size of the arrival-time difference $\mathbf {\Delta A}$. If $|\mathbf {\Delta A}|$ does not reach the threshold for successiveness detection, $c_{su}$, simultaneity is perceived ($I_{si}$). If $|\mathbf {\Delta A}|$ reaches the threshold for order detection, $c_{o}$ (with $c_{o} \ge c_{su}$), order is perceived ($I_{xy}$ and $I_{yx}$ for positive and negative values of $\mathbf {\Delta A}$). Finally, if $c_{su} \le |\mathbf {\Delta A}| < c_{o}$, $I_{su}$ results. In this case, $R_{xy}$ is provided with guessing probability *g*, and $R_{yx}$ with the complementary probability $1 - g$. The TTM thus allows for “order-uncertainty bands” at the two response boundaries in the ternary-response task (see Fig. 1). Theoretically, this implies another perceptual state besides order perception and simultaneity perception, namely the perception of “unordered successiveness”. In this way, the TTM bears resemblance to other two-threshold models of signal detection (Krantz, 1969) and pitch discrimination (Wickelgren, 1969).

### Present study

The goal of the presented research was to systematically evaluate the explanatory power of the three independent-channels models (REM, TSM, TTM) informally introduced above. To this end, these models were compared in terms of their balance between goodness of fit and parsimony against many data sets collected over a span of more than a century. Moreover, the model assumptions are discussed in relation to empirical findings and theoretical questions such as whether successiveness detection is necessary or even necessary and sufficient for order detection. In the next section, the three models to be compared are formalized.

## Formalization of the models

The specification of the REM and the TSM provided by García-Pérez and Alcalá-Quintana (2012b) and Jaśkowski (1991b) differ in several respects that are not the main focus of the present study. In particular, García-Pérez and Alcalá-Quintana (2012b) assumed that the arrival-latency difference $\mathbf {\Delta L}$ follows a Laplace (bilateral exponential) distribution, whereas Jaśkowski (1991b) assumed $\mathbf {\Delta L}$ to be normally distributed. To attribute the model selection results to the differences of interest here (role of response errors, separate successiveness and order processes, or unequal thresholds), all models had to be brought onto a common basis. Therefore, we assumed that $\mathbf {\Delta L}$ is normally distributed with mean $\mu _{\mathbf {\Delta L}}$ and variance $\sigma ^2_{\mathbf {\Delta L}}$. A normal distribution was assumed because it provides a simple and, in many realistic scenarios, satisfactory approximation of the difference between two Erlang-distributed or Wald-distributed arrival latencies (see Appendix B). However, to check whether our conclusions are robust to the use of different distributional assumptions, we repeated the model comparison with the alternative assumption that $\mathbf {\Delta L}$ is Laplace-distributed with location $\mu _{\mathbf {\Delta L}}$ and scale $b_{\mathbf {\Delta L}}$. As described below, this led to the selection of the same model, thus giving hope that the results of the present model comparison are not specific to our particular distributional assumptions in this study.

**Fig. 2:**
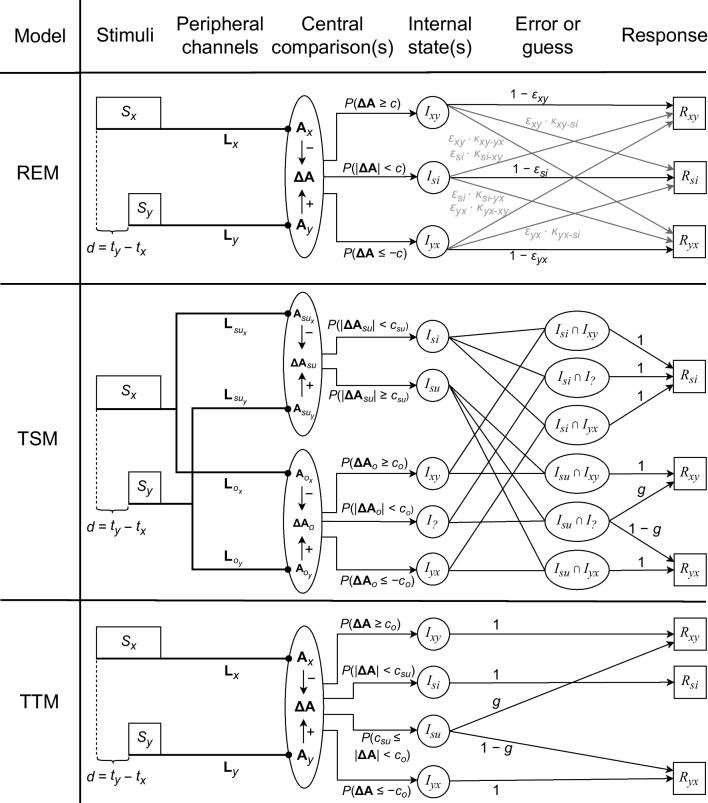
Response-error model (REM), two-stage model (TSM), and two-threshold model (TTM). *Note.* *S*: stimulus; *x*: one channel (e.g., vision); *y*: other channel (e.g., audition); *t*: stimulus-onset time; *d*: stimulus-onset difference; $\textbf{L}$: latency; $\textbf{A}$: arrival time; $\mathbf {\Delta A}$: arrival-time difference; *c*: threshold (i.e., criterion); *su*: successiveness (or: *x* and *y* successive); *o*: order; *I*: internal state; *xy*: *x* before *y*; *si*: *x* simultaneous to *y*; *yx*: *x* after *y*; ?: imperceptible order; $\epsilon$: response-error probability; $\kappa$: conditional misreport probability; *g*: guessing probability; *R*: response

Furthermore, we assumed that the threshold for successiveness detection, $c_{su}$, is constant and symmetric around the simultaneity band. The three parameters $\mu _{\mathbf {\Delta L}}$, $\sigma _{\mathbf {\Delta L}}$, and $c_{su}$ yield a basic independent-channels model, according to which $I_{yx}$ results if $\mathbf {\Delta A} \le -c_{su}$, $I_{si}$ if $|\mathbf {\Delta A}| < c_{su}$, and $I_{xy}$ if $\mathbf {\Delta A} \ge c_{su}$. This basic model can, for instance, be conceived as a triggered-moment model, where the first central arrival of a peripheral signal elicits the start of a new moment. Because each of the three models (REM, TSM, TTM) comprises the three parameters $\mu _{\mathbf {\Delta L}}$, $\sigma _{\mathbf {\Delta L}}$, and $c_{su}$, they all represent extensions of this basic independent-channels model. These extended models are described below and illustrated in Fig. 2. Appendix A includes the derivations of the formulas for the internal-state probabilities according to the three models.

### Response-error model (REM)

Unlike the TSM and the TTM, the REM requires that successiveness and order are detected if $\mathbf {\Delta A}$ reaches a single threshold $c = c_{su} = c_{o}$. Importantly, the REM introduces the response-error rates $\epsilon _{xy}$, $\epsilon _{si}$, and $\epsilon _{yx}$ that reflect the probability of misreporting $I_{xy}$, $I_{si}$, and $I_{yx}$, respectively. Moreover, $\kappa _{xy-yx}$, $\kappa _{si-xy}$, and $\kappa _{yx-xy}$ denote the conditional probability of misreporting $I_{xy}$ as $R_{yx}$, $I_{si}$ as $R_{xy}$, and $I_{yx}$ as $R_{xy}$ given that the respective internal state is misreported. It follows that $\kappa _{xy-si} = 1 - \kappa _{xy-yx}$, $\kappa _{si-yx} = 1 - \kappa _{si-xy}$, and $\kappa _{yx-si} = 1 - \kappa _{yx-xy}$. Unlike García-Pérez and Alcalá-Quintana (2012a, 2012b), we did not include an additional parameter $\tau$, which reflects a further processing delay for one channel.^5^

The resulting nine-parameter model predicts that

1a $$\begin{aligned} P(R_{xy}\,|\,d)&= (1 - \epsilon _{xy}) \cdot P(I_{xy}\,|\,d) \nonumber \\&\quad + \epsilon _{si} \cdot \kappa _{si-xy} \cdot P(I_{si}\,|\,d) \nonumber \\&\quad + \epsilon _{yx} \cdot \kappa _{yx-xy} \cdot P(I_{yx}\,|\,d), \end{aligned}$$

1b $$\begin{aligned} P(R_{si}\,|\,d)&= \epsilon _{xy} \cdot \kappa _{xy-si} \cdot P(I_{xy}\,|\,d) \nonumber \\&\quad + (1 - \epsilon _{si}) \cdot P(I_{si}\,|\,d) \nonumber \\&\quad + \epsilon _{yx} \cdot \kappa _{yx-si} \cdot P(I_{yx}\,|\,d), \end{aligned}$$

1c $$\begin{aligned} P(R_{yx}\,|\,d)&= \epsilon _{xy} \cdot \kappa _{xy-yx} \cdot P(I_{xy}\,|\,d) \nonumber \\&\quad + \epsilon _{si} \cdot \kappa _{si-yx} \cdot P(I_{si}\,|\,d) \nonumber \\&\quad + (1 - \epsilon _{yx}) \cdot P(I_{yx}\,|\,d). \end{aligned}$$

The frequency and direction of response errors may differ across studies depending on how the response buttons are arranged (García-Pérez & Alcalá-Quintana, 2012a, b). Therefore, we followed García-Pérez and Alcalá-Quintana (2012a) and Alcalá-Quintana and García-Pérez (2013) by fitting seven reduced versions of the REM to the data from each study: One three-parameter “zero model” $\text {REM}_{\emptyset }$ – which forms the base of REM, TSM, and TTM alike – has no response-error or -bias parameter at all. Three five-parameter models ($\text {REM}_{xy}$, $\text {REM}_{si}$, $\text {REM}_{yx}$) have an error parameter and a bias parameter for one response. Finally, three seven-parameter models ($\text {REM}_{xy, si}$, $\text {REM}_{xy, yx}$, $\text {REM}_{si, yx}$) have an error parameter and a bias parameter for two responses. The full nine-parameter $\text {REM} = \text {REM}_{xy, si, yx}$, which includes an error parameter and a bias parameter for each of the three responses in the ternary-response task, was superior to all reduced REMs for seven out of eight studies (see Table S24 in the Supplementary Material). It is thus not surprising that the $\text {REM}_{\textrm{min}}$, which takes the form of the best model for each study, performs the same as the full-fledged REM when compared to the TSM and the TTM. Therefore, we only consider the nine-parameter REM in the following.

### Two-stage model (TSM)

The TSM includes separate arrival-latency differences $$\mathbf {\Delta L}_{su} \sim N(\mu _{\mathbf {\Delta L}_{su}}, \sigma ^2_{\mathbf {\Delta L}_{su}})$$, $$\mathbf {\Delta L}_o \sim N(\mu _{\mathbf {\Delta L}_{o}}, \sigma ^2_{\mathbf {\Delta L}_{o}})$$ as well as separate thresholds $$c_{su}$$, $$c_{o}$$ for successiveness and order detection. It is posited that $$I_{si}$$ in the successiveness center always leads to $$R_{si}$$ irrespective of the internal state in the order center ($$I_{xy}$$, $$I_{yx}$$, or $$I_{?}$$). However, for $$I_{su}$$, $$I_{xy}$$ leads to $$R_{xy}$$, $$I_{yx}$$ to $$R_{yx}$$, and $$I_{?}$$ to $$R_{xy}$$ ($$R_{yx}$$) with probability *g* ($$1 - g)$$.^6^ The seven-parameter model thus predicts that

2a $$\begin{aligned} P(R_{xy}\,|\,d)&= P(I_{su}\,\cap \,I_{xy}\,|\,d) \nonumber \\&\quad + g \cdot P(I_{su}\,\cap \,I_{?}\,|\,d), \end{aligned}$$

2b $$\begin{aligned} P(R_{si}\,|\,d)&= P(I_{si} \cap I_{xy}\,|\,d) + P(I_{si} \cap I_{?}\,|\,d) \nonumber \\&\quad + P(I_{si} \cap I_{yx}\,|\,d) = P(I_{si}\,|\,d), \end{aligned}$$

2c $$\begin{aligned} P(R_{yx}\,|\,d)&= P(I_{su}\,\cap \,I_{yx}\,|\,d) \nonumber \\&\quad + (1 - g) \cdot P(I_{su}\,\cap \,I_{?}\,|\,d). \end{aligned}$$

### Two-threshold model (TTM)

Similar to the TSM, the TTM includes separate thresholds for the detection of successiveness ($c_{su}$) and order ($c_{o}$). However, while the TSM allows $c_{su}$ and $c_{o}$ to vary independently, the TTM imposes the constraint that $c_{o} \ge c_{su}$. Furthermore, *g* ($1 - g$) here reflects the probability of reporting $I_{su}$ as $R_{xy}$ ($R_{yx}$).^7^ Unlike the TSM, but like the REM, the TTM assumes that successiveness and order detection are based on the same arrival-latency difference $\mathbf {\Delta L} \sim N(\mu _{\mathbf {\Delta L}}, \sigma ^2_{\mathbf {\Delta L}})$. Like the TSM, but unlike the REM, the TTM assumes that observers do not produce response errors, or at least that they are so rare such that they can be ignored for parsimony in modeling. As a result, the TTM adds only two parameters to a basic independent-channels model that only has the three parameters $\mu _{\mathbf {\Delta L}}$, $\sigma ^2_{\mathbf {\Delta L}}$, and $c_{su}$.

Each of the two additional parameters is intended to address a shortcoming of many independent-channels models: By assuming that the threshold for order detection can exceed the threshold for successiveness detection (i.e., $c_{o} \ge c_{su}$), one can account for non-monotonic psychometric functions $P(R_{xy}\,|\,d)$ and $1 - P(R_{yx}\,|\,d)$ (even if $R_{xy}$ and $R_{yx}$ were given at random for $I_{su}$). By further adding the response-bias *g*, one can also account for differently shaped non-parallel psychometric functions $P(R_{xy}\,|\,d)$ and $1 - P(R_{yx}\,|\,d)$ by capturing the subject’s response tendency when uncertain about the temporal order of stimuli perceived as successive. Thus, both additional parameters in the TTM seem justified. According to the resulting five-parameter model, the predicted response probabilities are

3a $$\begin{aligned}&P(R_{xy}\,|\,d) = P(I_{xy}\,|\,d) + g \cdot P(I_{su}\,|\,d),\end{aligned}$$

3b $$\begin{aligned}&P(R_{si}\,|\,d) = P(I_{si}\,|\,d),\end{aligned}$$

3c $$\begin{aligned}&P(R_{yx}\,|\,d) = P(I_{yx}\,|\,d) + (1 - g) \cdot P(I_{su}\,|\,d). \end{aligned}$$

## Method

### Studies

We considered data from ternary-response tasks and data from other tasks that could be plausibly converted to the responses $R_{xy}$, $R_{si}$, and $R_{yx}$. As a result, data from eight studies were included in the present model comparison. These studies differed in several respects (e.g., stimulus modality, stimulus type, response format). Most notably, some studies used intramodal stimuli (i.e., two visual stimuli), whereas other studies used intermodal stimuli (i.e., a visual stimulus and an auditory stimulus). However, the models considered here are sufficiently general to be applicable to all these different experimental settings. A brief description of the eight studies follows.

Benussi (1913, pp. 363–369) conducted three experiments using a ternary-response task. The onset of two horizontally displaced light flashes was manipulated with $d = -150, -120, ..., 150$ ms in Experiment 1, and $d = -90, -60, ..., 90$ ms in Experiments 2 and 3 (small rounding errors due to actual step size of 29.7 ms). For each experiment and each value of *d*, Benussi (1913) added the number of trials in which a particular response was given across all 32 subjects. By dividing this sum by the total number of trials for each value of *d*, an average psychometric function across all subjects was obtained for each of the three experiments.

Allan (1975a) tasked three subjects to indicate their SJ ($R_{si}$ or $R_{su}$) followed by their TOJ ($R_{xy}$ or $R_{yx}$) with regard to the stimulus-offset difference ($d = -100, -75, ..., 100$ ms) of a light and a tone (see also Yarrow et al., 2011). In accordance with Ulrich (1987) and Jaśkowski (1991b), we coded $R_{su}$ followed by $R_{xy}$ ($R_{yx}$) as $R_{xy}$ ($R_{yx}$), and $R_{si}$ followed by $R_{xy}$ or $R_{yx}$ as $R_{si}$.

Allan (1975b) supplemented an audio-visual TOJ task (stimulus-offset difference $d = -100, -75, ..., 100$ ms; six subjects) with a confidence rating, yielding the four response alternatives $R_{xy\,\cap \,certain}$, $R_{xy\,\cap \,uncertain}$, $R_{yx\,\cap \,uncertain}$, and $R_{yx\,\cap \,certain}$ (see also Arnold et al., 2020; Keane et al., 2015). Following Ulrich (1987) and Jaśkowski (1991b), we coded $R_{xy\,\cap \,certain}$ as $R_{xy}$, $R_{xy\,\cap \,uncertain}$ and $R_{yx\,\cap \,uncertain}$ both as $R_{si}$, and $R_{yx\,\cap \,certain}$ as $R_{yx}$.

Ulrich (1987) used a ternary-response task with regard to the onset of two vertically displaced light flashes ($d = -100, -75, ..., 100$ ms). For each of the three subjects, the intensity of the stimuli was manipulated so that both visual stimuli were equally bright in the high-intensity condition but equally dim in the low-intensity condition.

Jaśkowski (1991a, Experiment 3) also devised a ternary-response task regarding the onset of two vertically displaced light flashes ($d = -75, -60, ..., 75$ ms). The task-irrelevant stimulus duration was manipulated within three subjects (bottom stimulus shorter, same duration, or longer than top stimulus). Note that the data from this study had to be read from a figure, which resulted in minimal deviations from the observed data.

Also van Eijk et al. (2008) applied an audio-visual ternary-response task ($d = -350, -300, ..., 350$ ms). It was manipulated across sessions whether the stimuli were simple (light flash and click) or complex (bouncing ball and appropriate impact sound). Twelve subjects were tested with the complex stimuli, of which eleven were also tested with the simple stimuli.

García-Pérez and Alcalá-Quintana (2018) asked 19 subjects to perform a quaternary-response task with the four response alternatives $R_{xy}$, $R_{yx}$, $R_{si}$, and $R_{su}$ (see also Weiß & Scharlau, 2010, 2011). The response $R_{su}$ here indicated that the stimuli appeared successive, but in an unclear temporal order. The onset of two horizontally displaced visual stimuli was manipulated in steps of 17 ms according to an adaptive procedure (García-Pérez, 2014). As predicted by the TTM and consistent with the TSM, $P(R_{su}\,|\,d)$ tended to follow a bimodal distribution, with the highest response probability left and right of the peak of $P(R_{si}\,|\,d)$. However, many subjects gave $R_{su}$ rarely or not all, possibly because they considered this response undesirable and therefore resorted to other response alternatives (Weiß & Scharlau, 2011). As noted by García-Pérez and Alcalá-Quintana (2018, p. 283), the psychometric functions of those subjects often exhibited non-monotonicities around the simultaneity band (i.e., at the locations at which two peaks of $R_{su}$ are expected based on the TTM). However, due to the rather infrequent use of $R_{su}$ and since it is unclear how the REM would have to be modified to fit the distribution of $R_{su}$ without invoking some form of TTM, trials with the response $R_{su}$ were excluded from the present analysis, and the three models were fitted to the distributions of the responses $R_{xy}$, $R_{si}$, and $R_{yx}$.

Finally, Lahkar et al. (2023) conducted a ternary-response task with two horizontally displaced light flashes ($d = -65, -60, ..., 65$ ms). Responses were summed across the 24 subjects for each of the 27 different values of *d*. Accordingly, we fitted the models to the data aggregated across all subjects.

### Model fitting

Each model was fitted to the observed frequency of $R_{xy}$, $R_{si}$, and $R_{yx}$ as a function of *d* by minimizing the likelihood-ratio goodness-of-fit statistic

4 $$\begin{aligned} G^2 = 2 \cdot \sum _{R} \sum _{d} O_{Rd} \cdot \textrm{ln} \, \frac{O_{Rd}}{E_{Rd}}, \end{aligned}$$

where $O_{Rd}$ ($E_{Rd}$) denotes the observed (expected) frequency of response $R \in \{R_{xy}, R_{si}, R_{yx}\}$ for a particular value of *d* (e.g., $d \in \{-100, -75, ..., 100\,\,\text {ms}\}$). It is well known that $G^2$ is inaccurate for small expected frequencies (see e.g., Cochran, 1952, 1954). Therefore, a somewhat arbitrary lower bound for expected frequencies was set at 0.1 to reduce the high leverage of very small expected frequencies. This improved the fits of all models, without affecting the outcome of the model selection.

The statistic $G^2$ was minimized using the quasi-Newton optimization algorithm L-BFGS-B. The parameter space was constrained as follows (initial parameter values in brackets): Mean arrival-latency differences ($\mu _{\mathbf {\Delta L}}$, $\mu _{\mathbf {\Delta L}_{su}}$, $\mu _{\mathbf {\Delta L}_{o}}$) could vary from $-100$ to 100 ms ($-33$, 34 ms), the standard deviations of arrival-latency differences ($\sigma _{\mathbf {\Delta L}}$, $\sigma _{\mathbf {\Delta L}_{su}}$, $\sigma _{\mathbf {\Delta L}_{o}}$) from 1 to 100 ms (33, 67 ms), thresholds (*c*, $c_{su}$, $c_{o}$) from 1 to 150 ms (50, 100 ms), response errors ($\epsilon _{xy}$, $\epsilon _{si}$, $\epsilon _{yx}$) from 0 to 0.8 (0.27, 0.53), and response biases ($\kappa _{xy-yx}$, $\kappa _{si-xy}$, $\kappa _{yx-xy}$, *g*) from 0 to 1 (0.33, 0.67). For each parameter, two initial values were evenly distributed in the search space (see above). Parameters were estimated for each combination of initial parameter values, thus yielding $2^9 = 512$ iterations for the REM, $2^7 = 128$ iterations for the TSM, and $2^5 = 32$ iterations for the TTM. Notably, parameter estimation based on such a multitude of initial values was necessary to identify the best-fitting parameter values of the REM and the TSM, whereas this turned out to be unnecessary for the TTM. That is, $G^2$ values varied as a function of the initial values for the REM and the TSM, but not for the TTM.

Because $G^2$ is only asymptotically $\chi ^2$-distributed, chi-square goodness-of-fit tests yield an inflation of significant results for small expected frequencies (García-Pérez, 1994; García-Pérez & Núñez-Antón, 2001, 2004), which were prevalent in many of the data sets considered here (e.g., Lahkar et al., 2023). Furthermore, estimation of confidence intervals via parametric bootstrapping (see Alcalá-Quintana & García-Pérez, 2013; García-Pérez & Alcalá-Quintana, 2012b; Wichmann & Hill, 2001) lead to large differences in the width of the confidence intervals for $G^2$ across the three models, which exhibited considerable differences in their best-fitting variability parameter values. It therefore seemed most sensible not to subject $G^2$ to a chi-square or parametric-bootstrap goodness-of-fit test, but instead to first assess the goodness of fit qualitatively based on visual inspection and then to offset goodness of fit ($G^2$) against model complexity in a quantitative model comparison. Note that each individual fit upon which the model comparison is based is presented either below or in the Supplementary Material.

Following previous TOJ model comparisons (e.g., García-Pérez & Alcalá-Quintana, 2012a; Lahkar et al., 2023; Tünnermann & Scharlau, 2018b; Wen et al., 2020), model selection was based on the Bayesian information criterion (BIC; Schwarz, 1978):

5 $$\begin{aligned} \text {BIC} = G^2 + k \cdot \text {ln}(n), \end{aligned}$$

where $G^2$ rewards goodness of fit and $k \cdot \text {ln}(n)$, with number of parameters *k* and number of observations *n*, punishes complexity. Thus, the model with the lowest overall BIC value across the eight studies was considered best. The BIC was used because it is consistent, that is, the probability of selecting the true (data-generating) model approximates 1 as *n* increases (e.g., Claeskens & Hjort, 2006). Unlike other information criteria such as the Akaike information criterion, the BIC therefore seemed appropriate for the objective of the present study, namely to compare the explanatory power of several candidate models.

Additionally, to quantify the specific ability of the models to fit non-monotonicities in the observed psychometric functions $P(R_{xy}\,|\,d)$ and $1 - P(R_{yx}\,|\,d)$ = $P(R_{xy} \cup R_{si}\,|\,d)$, we applied a non-monotonicity index (NMI):

6 $$\begin{aligned} \text {NMI} = \sum _{R} \sum _{i}\ \text {max}[0,\ P(R\,|\,d_{i}) - P(R\,|\,d_{i + 1})], \end{aligned}$$

where $R \in \{R_{xy}, R_{xy} \cup R_{si}\}$ and *i* runs from the first to the penultimate value of *d*. The higher the NMI (where NMI $\ge 0$), the stronger the non-monotonicity in the functions. The NMI was calculated for both observed and fitted psychometric functions to substantiate our inference from visual inspection that the models fitted the observed non-monotonic curves with different success.

**Fig. 3:**
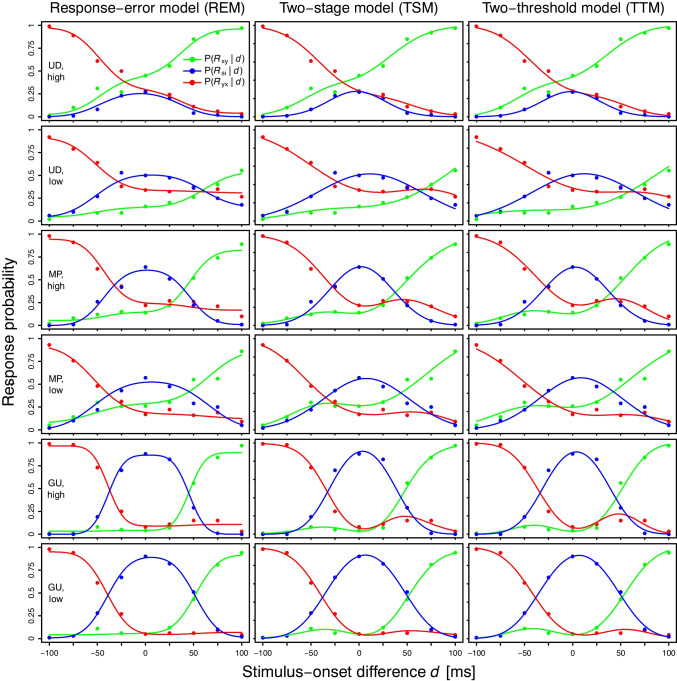
Observed (*points*) and fitted (*lines*) psychometric functions $P(R_{xy}\,|\,d)$, $P(R_{si}\,|\,d)$, and $P(R_{yx}\,|\,d)$ for the study by Ulrich (1987). *Note.* *x*: *top*; *y*: *bottom*

**Fig. 4:**
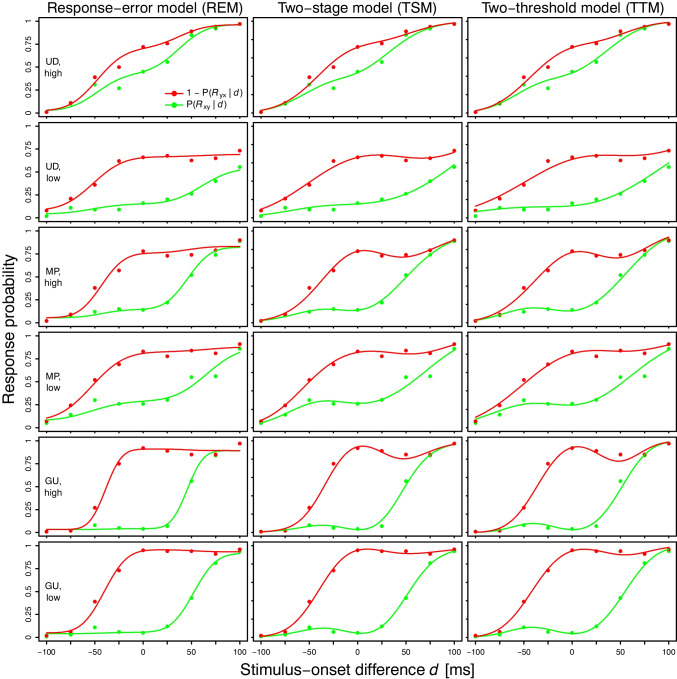
Observed (*points*) and fitted (*lines*) psychometric functions $P(R_{xy}\,|\,d)$ and $1 - P(R_{yx}\,|\,d)$ for the study by Ulrich (1987). *Note.* *x*: *top*; *y*: *bottom*

## Results

In general, visual inspection revealed that all three models fitted the observed psychometric functions $P(R_{xy}\,|\,d)$, $P(R_{si}\,|\,d)$, and $P(R_{yx}\,|\,d)$ well. As an example, Fig. 3 shows the model fits to the individual data from Ulrich (1987). Individual fits for all other studies are depicted in the Figs. S1–13 in the Supplementary Material. In addition, the observed and fitted psychometric functions $P(R_{xy}\,|\,d)$ and $1 - P(R_{yx}\,|\,d)$ are displayed in Fig. 4 for Ulrich (1987) and in the Figs. S14–26 in the Supplementary Material for the other studies. These figures allow a visual assessment of the shape characteristics (monotonicity, parallelism) that are predicted by many independent-channels models (perceptual-moment, triggered-moment, and attention-switching models) in the observed data and in the fits of the three independent-channels models considered here (REM, TSM, TTM).

First, the observed psychometric functions were often not monotonic (see e.g., subject GU, high-intensity condition in Fig. 4). In fact, non-monotonic curves were found in each of the eight studies considered. It is notable that the fits of the TSM and the TTM tended to follow non-monotonic trends in the data, whereas the REM fits rarely did so (see e.g., Fig. 4 as well as Figs. S17–25 in the Supplementary Material). Specifically, in the REM fits, the height of the asymptotes was often adjusted without taking into account the observed “bumps”. This does not imply that the REM cannot account for non-monotonic psychometric functions at all, since (1) its fits were non-monotonic in some cases (e.g., for subject 2 from García-Pérez and Alcalá-Quintana, 2018; see Fig. S23 in the Supplementary Material), and more generally, (2) mathematical models can typically generate various curves, not just the ones produced by their best-fitting parameter values (Roberts & Pashler, 2000). Nevertheless, the TSM and the TTM appeared more sensitive to non-monotonic curves compared to the REM. Quantitatively, this is supported by the fact that the NMI for the TSM fits and the TTM fits were much closer to the NMI in the observed psychometric functions than the NMI for the REM fits (see Table S25 in the Supplementary Material). Second, the observed psychometric functions $P(R_{xy}\,|\,d)$ and $1 - P(R_{yx}\,|\,d)$ were often not parallel (see e.g., subject UD, high-intensity condition in Fig. 4). The fits of all three models followed such non-parallel curves.

Crucially, the total BIC value summed across all studies was lowest for the TTM, second lowest for the TSM, and highest for the REM (see Table 1). Accordingly, the TTM provided the most satisfactory account of the three models. The selection of the TTM appeared to be fairly robust: For six of the eight studies considered, the TTM provided the lowest BIC value, with the TSM yielding the lowest value for the study by Allan (1975b) and the REM for the study by van Eijk et al. (2008).

When comparing the models separately for studies with intramodal (i.e., two visual) stimuli (Benussi, 1913; Ulrich, 1987; Jaśkowski, 1991a; García-Pérez & Alcalá-Quintana, 2018; Lahkar et al., 2023) and for studies with intermodal (i.e., visual and auditory) stimuli (Allan, 1975a, b; van Eijk et al., 2008), the TTM provided the lowest overall BIC value in both cases. However, while the TTM yielded the lowest BIC value for each of the five studies with intramodal stimuli, the preference for the TTM was less pronounced and not consistent across the three studies with intermodal stimuli (see Table 1).^8^

**Table 1:** Sum of $G^2$ and BIC values for the three independent-channels models with the assumption of normally distributed arrival-latency differences across subjects and conditions within and across studies

Study	$G^2_{\text {\tiny REM}}$	$G^2_{\text {\tiny TSM}}$	$G^2_{\text {\tiny TTM}}$	$\text {BIC}_{\text {\tiny REM}}$	$\text {BIC}_{\text {\tiny TSM}}$	$\text {BIC}_{\text {\tiny TTM}}$
Benussi (1913)	45.1	87.2	98.8	242.4	240.7	208.4
Allan (1975a)	40.0	45.2	46.7	232.1	194.6	153.5
Allan (1975b)	52.2	87.1	217.7	452.5	398.4	440.1
Ulrich (1987)	121.6	76.0	101.9	504.4	373.8	314.6
Jaśkowski (1991a, Experiment 3)	678.6	526.8	618.5	1236.5	960.7	928.4
van Eijk et al. (2008)	653.1	1199.0	1335.3	2061.2	2294.2	2117.6
García-Pérez and Alcalá-Quintana (2018)	459.3	419.4	574.5	1535.0	1256.0	1172.1
Lahkar et al. (2023)	76.0	118.3	92.4	155.4	180.0	136.5
$\sum$	2125.9	2559.0	3085.9	6419.5	5898.4	5471.2

**Table 2:** Sum of $G^2$ and BIC values for the three independent-channels models with the alternative assumption of Laplace-distributed arrival-latency differences across subjects and conditions within and across studies

Study	$G^2_{\text {\tiny REM}}$	$G^2_{\text {\tiny TSM}}$	$G^2_{\text {\tiny TTM}}$	$\text {BIC}_{\text {\tiny REM}}$	$\text {BIC}_{\text {\tiny TSM}}$	$\text {BIC}_{\text {\tiny TTM}}$
Benussi (1913)	54.1	53.3	101.9	251.4	206.8	211.6
Allan (1975a)	43.9	51.9	59.6	236.1	201.3	166.3
Allan (1975b)	54.7	87.9	237.3	455.0	399.2	459.7
Ulrich (1987)	152.9	116.1	147.3	535.7	413.9	360.0
Jaśkowski (1991a, Experiment 3)	720.0	465.5	614.8	1277.9	899.4	924.7
van Eijk et al. (2008)	671.6	949.2	1245.9	2079.7	2044.4	2028.1
García-Pérez and Alcalá-Quintana (2018)	517.6	432.8	623.7	1593.3	1269.4	1221.3
Lahkar et al. (2023)	104.9	93.1	127.9	184.2	154.8	172.0
$\sum$	2319.8	2249.8	3158.4	6613.3	5589.2	5543.7

Moreover, the BIC value of the TTM was lower than the minimum BIC value across a hierarchy of eight REMs with different types and numbers of response-error and -bias parameters. This suggests that the REM is inferior to the TTM even when taking into account that the frequency and direction of response errors might depend on the set-up in a particular study (e.g., arrangement of response buttons).

Finally, the TTM also yielded the lowest overall BIC value when comparing (otherwise equivalent) models with the alternative assumption of Laplace-distributed arrival-latency differences (see Table 2 and Figs. S27–54 in the Supplementary Material). When comparing the individual models assuming a normal distribution and a Laplace distribution, the normal distribution provided the better fits in the REM and in the TTM, whereas the Laplace distribution provided the better fits in the TSM. Thus, taken together, the TTM with the normal distribution assumption performed best.

The mean best-fitting parameter values of the REM, TSM, and TTM can be found in the Tables 3, 4, and 5. Best-fitting mean arrival-latency differences were similar across models, with the exception of $\mu _{\mathbf {\Delta L}_o}$ in the TSM, which sometimes differed greatly. Surprisingly, the mean best-fitting TSM parameters $\mu _{\mathbf {\Delta L}_{su}}$ and $\mu _{\mathbf {\Delta L}_o}$ had different signs in three out of eight studies. In these cases, the TSM fits seem to suggest a shorter mean arrival latency of one stimulus (e.g., $S_x$) in the successiveness center but of the other stimulus (e.g., $S_y$) in the order center. The standard deviation of the arrival-latency difference was estimated to be lowest for the REM, medium for the TTM and for the successiveness center of the TSM, and highest for the order center of the TSM.

**Table 3:** Mean best-fitting REM parameter values across subjects and conditions within and across studies

Study	$\mu _{\mathbf {\Delta L}}$	$\sigma _{\mathbf {\Delta L}}$	*c*	$\epsilon _{xy}$	$\epsilon _{si}$	$\epsilon _{yx}$	$\kappa _{xy-yx}$	$\kappa _{si-xy}$	$\kappa _{yx-xy}$
Benussi (1913)	2	24	43	0.11	0.34	0.10	0.72	0.41	0.77
Allan (1975a)	$-13$	28	55	0.09	0.34	0.13	0.94	0.51	0.69
Allan (1975b)	$-13$	24	63	0.16	0.35	0.15	0.43	0.70	0.32
Ulrich (1987)	$-3$	21	48	0.16	0.38	0.05	0.90	0.46	0.89
Jaśkowski (1991a, Experiment 3)	$-1$	10	31	0.12	0.22	0.07	0.97	0.58	0.75
van Eijk et al. (2008)	$-26$	40	113	0.05	0.09	0.05	0.52	0.60	0.61
García-Pérez and Alcalá-Quintana (2018)	$-2$	18	50	0.07	0.16	0.08	0.73	0.51	0.66
Lahkar et al. (2023)	0	12	27	0.04	0.03	0.03	0.37	0.32	0.77
*M*	$-7$	22	54	0.10	0.24	0.08	0.69	0.51	0.68

**Table 4:** Mean best-fitting TSM parameter values across subjects and conditions within and across studies

Study	$\mu _{\mathbf {\Delta L}_{su}}$	$\mu _{\mathbf {\Delta L}_{o}}$	$\sigma _{\mathbf {\Delta L}_{su}}$	$\sigma _{\mathbf {\Delta L}_{o}}$	$c_{su}$	$c_{o}$	*g*
Benussi (1913)	1	$-59$	35	52	31	73	0.83
Allan (1975a)	$-11$	$-10$	41	59	37	54	0.50
Allan (1975b)	$-14$	$-38$	53	39	50	72	0.78
Ulrich (1987)	$-5$	$-19$	32	39	30	52	0.55
Jaśkowski (1991a, Experiment 3)	$-1$	$-16$	15	20	24	40	0.69
van Eijk et al. (2008)	$-26$	4	57	63	108	97	0.40
García-Pérez and Alcalá-Quintana (2018)	4	$-4$	25	33	45	59	0.56
Lahkar et al. (2023)	0	28	14	17	27	31	0.02
*M*	$-7$	$-14$	34	40	44	60	0.54

Furthermore, the threshold was estimated to be lowest for detecting successiveness (TSM, TTM), medium for the REM, and highest for detecting order (TSM, TTM). Particularly, in seven out of eight studies, the mean best-fitting TSM parameter values conformed to the constraint of the TTM that the order threshold is at least as high as the successiveness threshold. The exception to this was the study from van Eijk et al. (2008), for which the estimated thresholds were generally much higher than in the other studies.

Response errors were estimated by the REM to occur quite frequently. This is particularly the case for $I_{si}$, which was estimated to be accidentally reported as $R_{xy}$ or $R_{yx}$ in an average of 24% of all trials. For Ulrich (1987), this value was as high as 38%, despite five practice sessions and ten experimental sessions. According to the mean best-fitting response-bias parameters of the REM, $I_{xy}$ ($I_{yx}$) was less likely to be reported as $R_{si}$ than as $R_{yx}$ ($R_{xy}$). This also hints at some region in the decision space where stimuli appear successive but their order cannot be resolved. Finally, the estimates of the response-bias parameter *g* in the TSM and the TTM differed substantially across subjects and studies, suggesting that this parameter is needed in the framework of the two models to account for different individual and study-related response tendencies. The trends in the best-fitting parameters reported here were similarly evident in the fits of the models assuming Laplace-distributed arrival-latency differences (see Tables S21–23 in the Supplementary Material).

## Discussion

The present study compared three independent-channels models, the REM (García-Pérez & Alcalá-Quintana, 2012b), the TSM (Jaśkowski, 1991b), and the TTM (inspired by previous ideas from Sternberg et al., 1975, 2023 and García-Pérez & Alcalá-Quintana, 2015b, 2018). In general, independent-channels models assume that stimuli are processed in separate peripheral channels. Temporal order (simultaneity) is perceived if the arrival-time difference of the stimuli at a central location reaches (misses) a certain threshold, which may reflect the duration of a perceptual moment or time quantum. More specifically, the three independent-channels models considered here were developed to explain non-monotonic and non-parallel psychometric functions generated in the ternary-response task. The REM attributes such shapes to finger errors, the TSM to independent simultaneity and order processing, and the TTM to a higher threshold for order detection than for successiveness detection. These three models were fitted to data from eight previous studies. In sum, the TTM provided the lowest BIC value and was thus considered superior to the TSM and the REM. A more detailed evaluation of each model follows.

**Table 5:** Mean best-fitting TTM parameter values across subjects and conditions within and across studies

Study	$\mu _{\mathbf {\Delta L}}$	$\sigma _{\mathbf {\Delta L}}$	$c_{su}$	$c_{o}$	*g*
Benussi (1913)	1	36	31	57	0.47
Allan (1975a)	$-11$	42	38	68	0.52
Allan (1975b)	$-13$	52	49	80	0.73
Ulrich (1987)	$-5$	32	30	63	0.45
Jaśkowski (1991a, Experiment 3)	$-1$	16	24	41	0.51
van Eijk et al. (2008)	$-26$	58	108	128	0.56
García-Pérez and Alcalá-Quintana (2018)	$-2$	22	45	61	0.57
Lahkar et al. (2023)	0	14	27	30	0.50
*M*	$-7$	34	44	66	0.54

### Response-error model (REM)

One issue with the REM is that, while it seemed to provide a good fit to non-parallel psychometric functions, it often did not fit non-monotonic curves. The REM is well-equipped to shift the asymptotes of the fitted psychometric functions up and down, and thus often provided a good fit to the data points at the ends of the curves. However, non-monotonicities were regularly due to a U-shaped curvature lateral to the simultaneity peak, which the REM cannot easily account for. Another issue with the REM is that its estimates of the response-error rate often seemed implausibly high, especially considering the amount of practice in some of the studies. Therefore, as argued by Tünnermann and Scharlau (2018b, p. 8), response-error parameters appear unwarranted for highly trained subjects, and might even lead to invalid estimates of the response-error rates. In any event, we commend García-Pérez and Alcalá-Quintana (2012b) for expanding the TOJ modeling repertoire by methods to account for response errors and attentional lapses.

The idea of explaining non-monotonic and non-parallel psychometric functions in terms of response errors also impresses with its conceptual simplicity: The basic independent-channels model outlined above is preserved and only supplemented with the possibility that any internal state is misreported as any of the other responses. Unfortunately, however, this supplementary assumption renders modeling rather complex. The basic independent-channels model with only three parameters already needs to be expanded by six parameters for the ternary-response task, and the quaternary-response task would even require a total of 12 additional response-error and -bias parameters (see Supplementary Material from García-Pérez & Alcalá-Quintana, 2018). Fortunately, estimates of the mean arrival-latency difference, which is arguably the psychologically most relevant parameter, turned out to be almost unaffected by whether response errors were modeled or not. The bottom line for most model applications (e.g., in the research on prior entry; for a review, see Spence & Parise, 2010) thus seems to be that neither considering nor ignoring response errors is likely to compromise the validity of one’s main conclusions.

### Two-stage model (TSM)

The TSM provided a good fit to non-monotonic and non-parallel psychometric functions. To achieve these fits, however, the TSM sometimes made use of implausible assumptions. Specifically, the TSM fits frequently indicated that one stimulus (e.g., a sound) arrived earlier in the successiveness center, whereas the other stimulus (e.g., a light) arrived earlier in the order center.

More generally, it is a far stretch from other independent-channels models to the TSM: The former proceed from the parsimonious assumption that SJs and TOJs are generated by the same internal process, whereas the TSM posits two distinct processes. In this respect, the TSM is conceptually closer to other dual-route models (Horsfall et al., 2021; Mitrani et al., 1986; Parise & Ernst, 2016, 2023; Stelmach & Herdman, 1991). For example, the temporal-profile model from Stelmach and Herdman (1991) assumes that temporal order (simultaneity) is determined by computing the difference (overlap) between the internal response functions elicited by the two stimuli. Similarly, in the multisensory-correlation detector proposed by Parise and Ernst (2016, 2023), pre-processed signals are subtracted (multiplied) to compute the lag (correlation) and thereby determine the temporal order (simultaneity) of stimuli (but see Yarrow et al., 2023).

The TSM offers a straightforward explanation for the sometimes uncorrelated PSS estimates from TOJ and SJ tasks (Linares & Holcombe, 2014; Love et al., 2013; van Eijk et al., 2008; Vatakis et al., 2008; but see Machulla et al., 2016; Maier et al., 2011; Sanders et al., 2011). This partial lack of correlation might be taken as evidence that TOJs and SJs are based on distinct processes. However, note that these PSS indices, which are typically obtained by fitting arbitrary functions to the observed data, at best reflect the interplay of sensory, decisional, and motor processes (e.g., perceptual latencies, decision thresholds, and response errors). Therefore, García-Pérez and Alcalá-Quintana (2012a, 2015a, 2015b, 2015c) compared TOJ and SJ task performance in more fine-grained model-based analyses. Their extensive analyses of many data sets pointed to invariant sensory parameters (e.g., arrival latencies) between the two tasks. This is consistent with the REM and the TTM, but casts doubt on the TSM. Besides, the TSM also struggles to account for within-trial relations of SJ and TOJ task performance (Allan, 1975a), as we will discuss in more detail below.

### Two-threshold model (TTM)

Like the TSM, the TTM provided a good fit to non-monotonic and non-parallel psychometric functions. Unlike the TSM, the TTM is committed to a specific origin of such shapes, namely a higher threshold for order detection than for successiveness detection. Put the other way around, compared to the TSM, the TTM imposes the additional constraints that SJs and TOJs are based on the same arrival-latency difference and that the threshold for order detection cannot be smaller than the threshold for successiveness detection.

These constraints are mostly in line with the modeling results from García-Pérez and Alcalá-Quintana (2012a, 2015a, 2015b, 2015c). As already mentioned, their analyses strongly suggested identical perceptual latencies in the two tasks. Further, in line with the TTM, they obtained larger threshold estimates for the TOJ task than for the SJ task in three studies (Capa et al., 2014; Li & Cai, 2014; Matthews & Welch, 2015). Interestingly, however, they found the reverse pattern in two other studies (Linares & Holcombe, 2014; van Eijk et al., 2008). This is reminiscent of the TSM fits in the present model comparison, which yielded a numerically larger threshold for order (vs. successiveness) detection in seven studies, but the reverse pattern for the results of van Eijk et al. (2008). An exploration of differences across all the studies considered here revealed that the two “deviant” studies (Linares & Holcombe, 2014; van Eijk et al., 2008) (1) covered a substantially wider range of *d* values and (2) yielded generally higher threshold estimates compared to all other studies, except for the study of Matthews and Welch (2015). Thus, one might speculate that the presence of large stimulus-onset differences led subjects to set sub-optimally high thresholds, such that threshold differences between SJ and TOJ no longer reflected differences in optimal task performance.

In addition, the TTM is in line with human performance in ternary- and quaternary-response tasks. In ternary-response tasks, non-monotonicities in the psychometric functions tended to occur at the locations at which the TTM allows them to occur – left and right of the simultaneity peak. In quaternary-response tasks, $R_{su}$ tended to be most frequent in precisely these regions of *d*, thus yielding a bimodal distribution (García-Pérez & Alcalá-Quintana, 2018; Weiß & Scharlau, 2011). Finally, the TTM also appears to be in line with psychophysiological data, which pointed to brain areas that are commonly activated during SJs and TOJs, additional regions that are more strongly activated during TOJs, but few to no regions that are more strongly activated during SJs (Binder, 2015; Love et al., 2018; Matsuzaki et al., 2014; Miyazaki et al., 2016). Taken together, the constraints imposed by the TTM seem to be largely supported by cognitive modeling, neural, and behavioral evidence.

The TTM is conceptually simpler than the TSM, and certainly computationally simpler than the TSM and the REM because its fits do not depend on the initial values for the numerical search procedure. However, the TTM does seem to require a re-interpretation of a “threshold”. In the traditional independent-channels models, the threshold is defined relative to the duration of a psychological moment (or a time quantum). However, according to the TTM, the threshold depends on whether observers are detecting order (as in the TOJ task) or successiveness (as in the SJ task). Thus, under the classic moment-conception of the threshold (e.g., Stroud, 1955), moments are prolonged in the TOJ (vs. SJ) task. Furthermore, in the ternary-response task, two psychological moments of different duration would need to elapse in parallel. We are not convinced that the psychological moment, so conceived, still represents a sensible concept. Instead, the TTM rather appeals to an interpretation of the threshold as somehow reflecting the amount of evidence needed for an observer to determine successiveness or order. Under the plausible assumption that evidence accrues over time rather than instantaneously, responses should be slower for higher thresholds. Thus, consistent with the TTM, responses have been found to be slower in the TOJ task than in the SJ task (Matsuzaki et al., 2014; Matthews et al., 2016; Pan & Huang, 2023).

### Relation of successiveness and order detection

For many decades now, a lingering question has been whether successiveness detection is necessary or even sufficient for order detection. Specifically, successiveness detection may be (1) necessary and sufficient (Allan & Kristofferson, 1974; Baron, 1969), (2) neither necessary nor sufficient (cf. Mitrani et al., 1986), or (3) necessary yet insufficient (Hirsh, 1959; Hirsh & Sherrick, 1961) for order detection. Each of these three positions is represented by one of the three independent-channels models considered here: (1) The REM requires that successiveness detection is necessary and sufficient for order detection, because SJs and TOJs are thought to be governed by the same sensory and decisional processes. (2) According to the TSM, successiveness detection is neither necessary nor sufficient for order detection, because SJs and TOJs are thought to be governed by independent sensory and decisional processes. (3) The TTM demands that successiveness detection is necessary but not sufficient for order detection, because SJs and TOJs are thought to be governed by the same sensory and decisional processes, except that the decisional threshold may be higher for TOJs than for SJs.

Many pieces of (psychophysical, cognitive modeling, mental chronometric, and psychophysiological) evidence described earlier indicate that successiveness detection does not suffice for order detection. Thus, we will now turn to the necessity assumption. Although successiveness detection may be regarded as a “logical prerequisite” (Arrouet et al., 2023, p. 364) for order detection, independent TOJ and SJ processes would make it possible to detect order without detecting successiveness.^9^ However, contrary to the independence assumption, Allan (1975a) observed that the conditional probability of a correct order response given a preceding correct successiveness response for the same stimulus pair was significantly higher than the unconditional probability of a correct order response in 19 out of 24 cases (three subjects $\times$ eight values of $d \ne 0$).

Critically, the data from Allan (1975a) also allow a more direct test of the necessity assumption: If order detection necessitates successiveness detection, order detection performance should be at chance level when stimuli are judged to be simultaneous. To test this prediction, we re-analyzed the data from Allan (1975a), and found that the probability of a correct order response after an incorrect simultaneity response $R_{si}$ differed significantly from 0.50 in only three out of 24 cases (12.5%). Even when summing responses across all values of *d*, a binomial test did not yield a significant deviation from 0.50 for either of the three subjects (AJ: $102/180 = 0.57$, $p = .086$; TM: $141 / 252 = 0.56$, $p = .068$; BP: $139 / 275 = 0.51$, $p = .904$). This somewhat surprising outcome suggests that successiveness detection is indeed necessary for order detection, as entailed by the TTM and the REM.

### Limitations of present study

In the present study, three independent-channels models (REM, TSM, TTM) were compared based on their fit to the psychometric functions observed in eight previous studies using more than two response alternatives. The preference for the TTM across the three studies using intermodal stimuli was less pronounced compared to the consistent preference for the TTM in the five studies using intramodal stimuli. Therefore, future studies with intermodal stimuli should ascertain whether the preference for the TTM holds for the judgment of temporal relations across sensory modalities.

Another limitation of the present study was that the model selection using the BIC rests on the simplifying rule that the models receive identical penalties for each parameter, irrespective of how much a particular parameter within a particular model can alter the predictions of that model. This approach appears unwarranted in some cases. For example, the order threshold allows for more flexibility in the TSM than in the TTM, since it is allowed to vary independently of the successiveness threshold in the TSM, whereas it is prohibited to fall below the successiveness threshold in the TTM. While this instance seems to strengthen our selection of the TTM, the overall impact of this simplifying rule remains unclear. Therefore, future model comparisons may go beyond enumerating the number of free parameters when taking into account how flexible the models are in fitting data.

Moreover, it would also be desirable to obtain a more precise specification of the shapes of the psychometric functions predicted by different independent-channels models. Such an approach is pursued in the current study by Sternberg et al. (2023), which evaluates several predictions of models closely related to the two-threshold model considered here concerning the cumulants of the psychometric functions.

Furthermore, independent-channels models have been subject to other critical tests apart from their predictions about the shape of psychometric functions. Perhaps most notably, independent-channels models have also been tested with respect to RT data (Heath, 1984) and to their assumptions of independent peripheral channels and independent peripheral and central processing (Sternberg & Knoll, 1973; Ulrich, 1987). An evaluation of independent-channels models based on such tests was also beyond the scope of the present study. Finally, the theoretical conclusions in the present study are yet to be experimentally validated based on new data.

### Conclusion

Many independent-channels models have been challenged by observations of non-monotonic and non-parallel psychometric functions in ternary-response tasks. Three independent-channels models attribute such shapes to response errors (REM), to distinct processes underlying SJs and TOJs (TSM), or to a larger threshold for order (vs. successiveness) detection (TTM). Of these three models, the TTM provided the best balance between goodness of fit and parsimony in the present model comparison. The TTM is also in line with many other results from different methods (cognitive modeling, psychophysics, mental chronometry, psychophysiology), which together seem to suggest that successiveness detection is necessary but not sufficient for order detection. Independent-channels models can thus be reconciled with non-monotonic and non-parallel psychometric functions in a simple and plausible way.

## Supplementary Information

Below is the link to the electronic supplementary material.

Supplementary file 1 (pdf 5668 KB)

## Figures and Tables

**Fig. 5:**
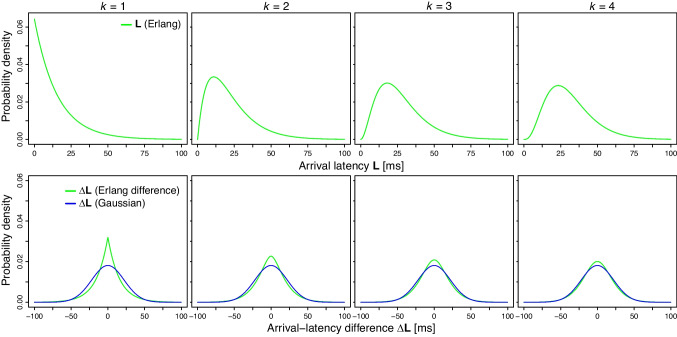
Theoretical comparison between the difference of two Erlang-distributed arrival latencies $\textbf{L} = \textbf{L}_x = \textbf{L}_y$ and the normally distributed arrival-latency difference $\mathbf {\Delta L}$

**Fig. 6:**
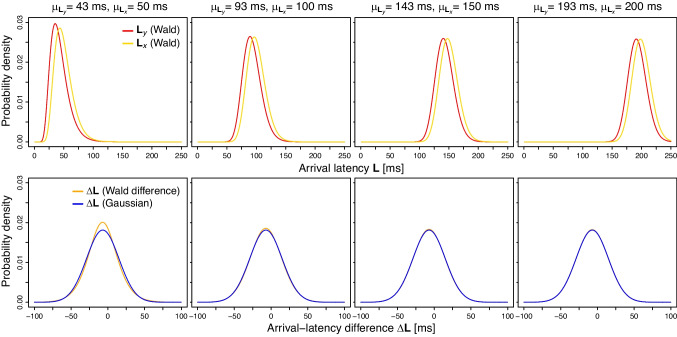
Theoretical comparison between the difference of two Wald-distributed arrival latencies $\textbf{L}_x$ and $\textbf{L}_y$ and the normally distributed arrival-latency difference $\mathbf {\Delta L}$

## Data Availability

R code to fit the models is available via the Open Science Framework at https://osf.io/ec2z3/.

## Appendix A: Internal-state probability formulas

### Response-error model (REM)

A1a $$\begin{aligned} P(I_{xy}\,|\,d) \nonumber&= P(\mathbf {\Delta A} \ge c) = P(\mathbf {\Delta L} + d \ge c) \\ \nonumber&= P(\mathbf {\Delta L} \ge c - d) = 1 - P(\mathbf {\Delta L} < c - d) \\&= 1 - F_{\mathbf {\Delta L}}(c - d) \end{aligned}$$

A1b $$\begin{aligned} P(I_{si}\,|\,d) \nonumber&= P(|\mathbf {\Delta A}|< c) = P(- c< \mathbf {\Delta A}< c) \\ \nonumber&= P(- c< \mathbf {\Delta L} + d< c) \\ \nonumber&= P(- c - d< \mathbf {\Delta L} < c - d) \\&= F_{\mathbf {\Delta L}}(c - d) - F_{\mathbf {\Delta L}}(- c - d) \end{aligned}$$

A1c $$\begin{aligned} P(I_{yx}\,|\,d) \nonumber&= P(\mathbf {\Delta A} \le - c) = P(\mathbf {\Delta L} + d \le - c) \\&= P(\mathbf {\Delta L} \le - c - d) = F_{\mathbf {\Delta L}}(- c - d) \end{aligned}$$

### Two-stage model (TSM)

A2a $$\begin{aligned} P(I_{si}\,|\,d) \nonumber&= P(|\mathbf {\Delta A}_{su}|< c_{su}) = P(- c_{su}< \mathbf {\Delta A}_{su}< c_{su}) \\ \nonumber&= P(- c_{su}< \mathbf {\Delta L}_{su} + d< c_{su}) \\ \nonumber&= P(- c_{su} - d< \Delta \textbf{L}_{su} < c_{su} - d) \\&= F_{\mathbf {\Delta L}_{su}}(c_{su} - d) - F_{\mathbf {\Delta L}_{su}}(- c_{su} - d) \end{aligned}$$

A2b $$\begin{aligned} P(I_{su}\,\cap \,I_{xy}\,|\,d) \nonumber&= P(|\mathbf {\Delta A}_{su}| \ge c_{su}) \cdot P(\mathbf {\Delta A}_{o} \ge c_o) \\ \nonumber&= [1 - P(|\mathbf {\Delta L}_{su} + d|< c_{su})] \cdot P(\mathbf {\Delta L}_{o} + d \ge c_o) \\ \nonumber&= [1 - P(\mathbf {\Delta L}_{su}< c_{su} - d) + P(\mathbf {\Delta L}_{su}< -c_{su} - d)] \\ \nonumber&\quad \cdot [1 - P(\mathbf {\Delta L}_o < c_o - d)] \\ \nonumber&= [1 - F_{\mathbf {\Delta L}_{su}}(c_{su} - d) + F_{\mathbf {\Delta L}_{su}}(-c_{su} - d)] \\&\quad \cdot [1 - F_{\mathbf {\Delta L}_o}(c_o - d)] \end{aligned}$$

A2c $$\begin{aligned} P(I_{su}\,\cap \,I_{?}\,|\,d) \nonumber&= P(|\mathbf {\Delta A}_{su}| \ge c_{su}) \cdot P(|\mathbf {\Delta A}_{o}|< c_o) \\ \nonumber&= [1 \!-\! P(|\mathbf {\Delta L}_{su} + d| \!<\! c_{su})] \cdot P(- c_o< \mathbf {\Delta L}_{o} + d< c_o)\\ \nonumber&= [1 - P(\mathbf {\Delta L}_{su}< c_{su} - d) + P(\mathbf {\Delta L}_{su}< - c_{su} - d)] \\ \nonumber&\quad \cdot P(- c_o - d< \mathbf {\Delta L}_o < c_o - d)\\ \nonumber&= [1 - F_{\mathbf {\Delta L}_{su}}(c_{su} - d) + F_{\mathbf {\Delta L}_{su}}(- c_{su} - d)] \\&\quad \cdot [F_{\mathbf {\Delta L}_o}(c_o - d) - F_{\mathbf {\Delta L}_o}(- c_o - d)] \end{aligned}$$

A2d $$\begin{aligned} P(I_{su}\,\cap \,I_{yx}\,|\,d) \nonumber&= P(|\mathbf {\Delta A}_{su}| \ge c_{su}) \cdot P(\mathbf {\Delta A}_{o} \le - c_o) \\ \nonumber&= [1 - P(|\mathbf {\Delta L}_{su} + d|< c_{su})] \cdot P(\mathbf {\Delta L}_{o} + d \le - c_o) \\ \nonumber&= [1 - P(\mathbf {\Delta L}_{su}< c_{su} - d) + P(\mathbf {\Delta L}_{su} < - c_{su} - d)] \\ \nonumber&\quad \cdot P(\mathbf {\Delta L}_o \le - c_o - d) \\ \nonumber&= [1 - F_{\mathbf {\Delta L}_{su}}(c_{su} - d) + F_{\mathbf {\Delta L}_{su}}(- c_{su} - d)] \\&\quad \cdot F_{\mathbf {\Delta L}_o}(- c_o - d) \end{aligned}$$

### Two-threshold model (TTM)

A3a $$\begin{aligned} P(I_{xy}\,|\,d) \nonumber&= P(\mathbf {\Delta A} \ge c_{o}) = P(\mathbf {\Delta L} + d \ge c_{o}) \\ \nonumber&= P(\mathbf {\Delta L} \ge c_{o} - d) = 1 - P(\mathbf {\Delta L} < c_{o} - d) \\&= 1 - F_{\mathbf {\Delta L}}(c_{o} - d) \end{aligned}$$

A3b $$\begin{aligned} P(I_{si}\,|\,d) \nonumber&= P(|\mathbf {\Delta A}|< c_{su}) = P(- c_{su}< \mathbf {\Delta A}< c_{su}) \\ \nonumber&= P(- c_{su}< \mathbf {\Delta L} + d< c_{su}) \\ \nonumber&= P(- c_{su} - d< \mathbf {\Delta L} < c_{su} - d) \\&= F_{\mathbf {\Delta L}}(c_{su} - d) - F_{\mathbf {\Delta L}}(- c_{su} - d) \end{aligned}$$

A3c $$\begin{aligned} P(I_{su}\,|\,d) \nonumber&= P(c_{su} \le |\mathbf {\Delta A}|< c_{o}) \\ \nonumber&= P(- c_{o}< \mathbf {\Delta A} \le - c_{su}) + P(c_{su} \le \mathbf {\Delta A}< c_{o}) \\ \nonumber&= P(- c_{o}< \mathbf {\Delta L} + d \le - c_{su}) + P(c_{su} \le \mathbf {\Delta L} + d< c_{o}) \\ \nonumber&= P(- c_{o} - d< \mathbf {\Delta L} \le - c_{su} - d) \\ \nonumber&\quad + P(c_{su} - d \le \mathbf {\Delta L} < c_{o} - d) \\ \nonumber&= F_{\mathbf {\Delta L}}(- c_{su} - d) - F_{\mathbf {\Delta L}}(- c_{o} - d) \\&\quad + F_{\mathbf {\Delta L}}(c_{o} - d) - F_{\mathbf {\Delta L}}(c_{su} - d) \end{aligned}$$

A3d $$\begin{aligned} P(I_{yx}\,|\,d) \nonumber&= P(\mathbf {\Delta A} \le - c_{o}) = P(\mathbf {\Delta L} + d \le - c_{o}) \\&= P(\mathbf {\Delta L} \le - c_{o} - d) = F_{\mathbf {\Delta L}}(- c_{o} - d) \end{aligned}$$

## Appendix B: Distributions of arrival-latency differences

### Difference of two Erlang distributions

Assume that the arrival-latency difference $\mathbf {\Delta L}$ is normally distributed with mean $\mu _{\mathbf {\Delta L}} = 0$ ms (for simplicity) and variance $\sigma ^2_{\mathbf {\Delta L}} = 22^2$ ms $= 484$ ms (following the mean best-fitting REM variability parameter value from Table 3). The probability density function (PDF) of the Erlang distribution is given by

B1 $$\begin{aligned} f_{\textbf{L}}(t, k, \lambda ) = \frac{\lambda ^k t^{k-1} e^{-\lambda t}}{(k-1)!}, \end{aligned}$$

with $$t > 0$$, $$k \in \{1, 2, ...\}$$, and $$\lambda > 0$$.

The parameter *k* can be conceptualized as the number of neural pulses required for stimulus detection (e.g., Luce & Green, 1972). For simplicity, assume that $\lambda _x = \lambda _y = \lambda$. In this case, the distribution of the difference of Erlang-distributed arrival latencies $\textbf{L}_x$ and $\textbf{L}_y$ has the mean

B2 $$\begin{aligned} \mu _{\mathbf {\Delta L}} = \mu _{\textbf{L}_y} - \mu _{\textbf{L}_x} = \frac{k}{\lambda } - \frac{k}{\lambda } = 0\ \textrm{ms} \end{aligned}$$

and the variance

B3 $$\begin{aligned} \sigma ^2_{\mathbf {\Delta L}} = \sigma ^2_{\textbf{L}_x} + \sigma ^2_{\textbf{L}_y} = \frac{k}{\lambda ^2} + \frac{k}{\lambda ^2} = \frac{2k}{\lambda ^2}. \end{aligned}$$

Rearranging yields

B4 $$\begin{aligned} \lambda = \sqrt{\frac{2k}{\sigma ^2_{\mathbf {\Delta L}}}}. \end{aligned}$$

Let the number of required neural pulses $k \in \{1, 2, 3, 4\}$ and $\sigma ^2_{\mathbf {\Delta L}} = 484$ ms (see above). For the special case of a single required pulse ($k = 1$), the Erlang distributions of $\textbf{L}_x$ and $\textbf{L}_y$ reduce to exponential distributions, and their difference $\mathbf {\Delta L}$ reduces to a Laplace distribution. In the first row of Fig. 5, the PDFs of the Erlang-distributed arrival latencies $\textbf{L}$ are shown for the four cases. In the second row, the difference between these PDFs is compared with the PDF of the normally distributed arrival-latency difference $\mathbf {\Delta L}$. The difference between the two Erlang distributions quickly approaches the normal distribution as the number of required neural pulses *k* increases. Despite the residual discrepancies in kurtosis, it may be doubted whether the difference of two Erlang distributions with $k > 1$ can be empirically distinguished from a normal distribution.

### Difference of two Wald distributions

Assume that the arrival-latency difference $\mathbf {\Delta L}$ is normally distributed with mean $\mu _{\mathbf {\Delta L}} = -7$ ms and variance $\sigma ^2_{\mathbf {\Delta L}} = 22^2$ ms $= 484$ ms (following the mean best-fitting REM parameter values from Table 3). Further, assume that $\sigma ^2_{\textbf{L}_x} = \sigma ^2_{\textbf{L}_y} = \sigma ^2_{\textbf{L}}$. Thus,

B5 $$\begin{aligned} \sigma ^2_{\mathbf {\Delta L}} = \sigma ^2_{\textbf{L}_x} + \sigma ^2_{\textbf{L}_y} = 2 \cdot \sigma ^2_{\textbf{L}}. \end{aligned}$$

Substituting $\sigma _{\mathbf {\Delta L}}^2 = 484$ ms yields $\sigma _{\textbf{L}}^2 = 242$ ms. The PDF of the Wald distribution reads

B6 $$\begin{aligned} f_\textbf{L}(t, \mu , \lambda ) = \sqrt{\frac{\lambda }{2 \pi t^3}} \cdot \exp \left[ -\frac{\lambda (t - \mu )^2}{2 \mu ^2 t}\right] , \end{aligned}$$

with $t > 0$, $\mu > 0$, and $\lambda = \mu ^3 / \sigma ^2 > 0$. Let $\mu _{\textbf{L}_x} \in \{50\,\textrm{ms},\,100\,\textrm{ms},\,150\,\textrm{ms},\,200\,\textrm{ms}\}$ and $\mu _{\textbf{L}_y} = \mu _{\textbf{L}_x} - 7$ ms, yielding $\mu _{\mathbf {\Delta L}} = -7$ ms in each of these four cases. In the first row of Fig. 6, the PDFs of the Wald-distributed arrival latencies $\textbf{L}_x$ and $\textbf{L}_y$ are shown for each case. In the second row, the difference of these PDFs is compared with the PDF of the normally distributed arrival-latency difference $\mathbf {\Delta L}$. The difference between two Wald distributions quickly approaches the normal distribution with increasing latencies. In fact, the two distributions are virtually indistinguishable starting from the second case, where $\mu _{\textbf{L}_x} = 100$ ms and $\mu _{\textbf{L}_y} = 93$ ms.

## Appendix C: Glossary

The main symbols and abbreviations used in the manuscript are explicated below. They are largely based on the terminologies used by Sternberg and Knoll (1973), Ulrich (1987), and Jaśkowski (1991b). Entries in this glossary are ordered by their first appearance in the manuscript.

TOJ: Temporal-order judgment
SJ: Simultaneity judgment
$S_x$ ($S_y$): Stimulus processed in peripheral channel *x* (*y*)
$t_x$ ($t_y$): Time at onset (or offset) of $S_x$ ($S_y$)
*d*: Time difference between onset (or offset) of $S_x$ and $S_y$ ($t_y - t_x$)
$R_{xy}$ ($R_{yx}$): Subject reports that $S_x$ appeared before (after) $S_y$
$R_{si}$ ($R_{su}$): Subject reports that $S_x$ and $S_y$ appeared simultaneous (successive)
PSS: Point of subjective simultaneity
$\textbf{L}_x$ ($\textbf{L}_y$): Arrival latency of $S_x$ ($S_y$) [REM, TTM]
$\textbf{A}_x$ ($\textbf{A}_y$): Arrival time of $S_x$ ($S_y$) [REM, TTM]
$\mathbf {\Delta L}$: Arrival-latency difference ($\textbf{L}_y - \textbf{L}_x$) [REM, TTM]
$\mathbf {\Delta A}$: Arrival-time difference ($\textbf{A}_y - \textbf{A}_x$) [REM, TTM]
*c* ($-c$): Threshold value of $\mathbf {\Delta A}$ above (below) which the internal state $I_{xy}$ ($I_{yx}$) is elicited (else: $I_{si}$) [REM]
REM: Response-error model
$I_{xy}$ ($I_{yx}$): Subject perceives $S_x$ before (after) $S_y$
$I_{si}$: Subject perceives $S_x$ and $S_y$ as simultaneous
TSM: Two-stage model
$I_{su}$: Subject perceives $S_x$ and $S_y$ as successive [TSM, TTM]
$I_{?}$: Subject does not perceive the order of $S_x$ and $S_y$ [TSM]
TTM: Two-threshold model
$c_{su}$: Threshold value of |$\mathbf {\Delta A}_{su}$| (TSM) or |$\mathbf {\Delta A}$| (TTM) below which $I_{si}$ is elicited [TSM, TTM]
$c_{o}$ ($-c_{o}$): Threshold value of $\mathbf {\Delta A}_o$ (TSM) or $\mathbf {\Delta A}$ (TTM) above (below) which $I_{xy}$ ($I_{yx}$) is elicited (else: $I_{?}$ for TSM, and $I_{su}$ for TTM if $|\mathbf {\Delta A}| \ge c_{su}$) [TSM, TTM]
*g* ($1 - g$): Probability that $I_{su} \cap I_{?}$ [TSM] or $I_{su}$ [TTM] is reported as $R_{xy}$ ($R_{yx}$) [TSM, TTM]
$\mu _{\mathbf {\Delta L}}$ ($\sigma ^2_{\mathbf {\Delta L}}$): Mean (variance) of $\mathbf {\Delta L}$ [REM, TTM]
$b_{\mathbf {\Delta L}}$: Scale of Laplace-distributed $\mathbf {\Delta L}$
$\epsilon _{xy}$: Probability that $I_{xy}$ is misreported as $R \in \{R_{si}, R_{yx}\}$ [REM]
$\epsilon _{si}$: Probability that $I_{si}$ is misreported as $R \in \{R_{xy}, R_{yx}\}$ [REM]
$\epsilon _{yx}$: Probability that $I_{yx}$ is misreported as $R \in \{R_{xy}, R_{si}\}$ [REM]
$\kappa _{xy-yx}$ ($\kappa _{xy-si}$): Probability that a misreport of $I_{xy}$ takes the form of $R_{yx}$ ($R_{si}$) [REM]
$\kappa _{si-xy}$ ($\kappa _{si-yx}$): Probability that a misreport of $I_{si}$ takes the form of $R_{xy}$ ($R_{yx}$) [REM]
$\kappa _{yx-xy}$ ($\kappa _{yx-si}$): Probability that a misreport of $I_{yx}$ takes the form of $R_{xy}$ ($R_{si}$) [REM]
$\textbf{L}_{su_x}$ ($\textbf{L}_{su_y}$): Arrival latency of $S_x$ ($S_y$) at the successiveness center [TSM]
$\mathbf {\Delta L}_{su}$: Arrival-latency difference for successiveness center ($\textbf{L}_{su_y} - \textbf{L}_{su_x}$) [TSM]
$\mu _{\mathbf {\Delta L}_{su}}$ ($\sigma ^2_{\mathbf {\Delta L}_{su}}$): Mean (variance) of $\mathbf {\Delta L}_{su}$ [TSM]
$\textbf{L}_{o_x}$ ($\textbf{L}_{o_y}$): Arrival latency of $S_x$ ($S_y$) at the order center [TSM]
$\mathbf {\Delta L}_o$: Arrival-latency difference for order center ($\textbf{L}_{o_y} - \textbf{L}_{o_x}$) [TSM]
$\mu _{\mathbf {\Delta L}_o}$ ($\sigma ^2_{\mathbf {\Delta L}_o}$): Mean (variance) of $\mathbf {\Delta L}_o$ [TSM]
$\textbf{A}_{su_x}$ ($\textbf{A}_{su_y}$): Arrival time of $S_x$ ($S_y$) at the successiveness center [TSM]
$\mathbf {\Delta A}_{su}$: Arrival-time difference at the successiveness center ($\textbf{A}_{su_y} - \textbf{A}_{su_x}$) [TSM]
$\textbf{A}_{o_x}$ ($\textbf{A}_{o_y}$): Arrival time of $S_x$ ($S_y$) at the order center [TSM]
$\mathbf {\Delta A}_{o}$: Arrival-time difference at the order center ($\textbf{A}_{o_y} - \textbf{A}_{o_x}$) [TSM]
BIC: Bayesian information criterion
NMI: Non-monotonicity index
PDF: Probability density function

## Footnotes

1 See Appendix C for a glossary of symbols and abbreviations used in this manuscript.

2 Occasionally, the stimulus-offset difference is manipulated instead of the stimulus-onset difference (e.g., Allan, 1975a, b). In this case, *d* refers to the stimulus-offset difference.

3 The seminal, but unpublished work from Sternberg et al. (1975) was recently released in extended form (Sternberg et al., 2023).

4 The name of each of the three considered models points to the characteristic that distinguishes it from a basic independent-channels model assuming a variable (e.g., normally distributed) arrival-latency difference $\mathbf {\Delta L}$ and a constant threshold *c*: (1) The response-error model (REM) allows for erroneous translations of internal states into responses, (2) the two-stage model (TSM) posits that the perception of simultaneity and temporal order are governed by two distinct processes (i.e., two independently distributed arrival-latency differences and two independent thresholds), and (3) the two-threshold model (TTM) assumes that the threshold for order detection may exceed the threshold for successiveness detection. Note that the TSM also assumes two thresholds, but unlike the TTM, the TSM is not limited to this, as it assumes two entirely distinct SJ and TOJ processes. We use the name TSM instead of the possibly more precise name “two-process model” to be consistent with the terminology introduced by Jaśkowski (1991b).

5 This parameter may be useful for studies in which attention is manipulated (e.g., by cueing one stimulus). However, none of the studies considered here includes such a manipulation. Furthermore, Tünnermann and Scharlau (2018b) found that the delay parameter $\tau$ interferes with the parameters reflecting the arrival latencies (here: the arrival-latency difference). They thus concluded that the use of $\tau$ should be avoided (see also Tünnermann & Scharlau, 2016; Tünnermann et al., 2017).

6 Unlike Jaśkowski (1991b), we did not include another response-bias parameter in the TSM to translate $I_{su} \cap I_{?}$ into $R_{si}$ because this complication would contradict the basic tenet of separate successiveness and order mechanisms if $I_{su}$ in the successiveness center could be “overwritten” by the order center.

7 Pilot modeling suggested that it is also not necessary for the TTM to allow the theoretically questionable possibility that $I_{su}$ is reported as $R_{si}$ (see also Footnote 6).

8 We refrain from strong interpretations of the clearer preference for the TTM with intramodal compared to intermodal stimuli, as the two subsets of studies did not exclusively differ in terms of modalities (e.g., judgment of stimulus-offset differences in Allan, 1975a, b).

9 Mitrani et al. (1986, p. 163) and Jaśkowski (1991b, p. 77) assumed that the order center is no longer evaluated after $I_{si}$ in single-response paradigms in order to prevent a double response. However, in the informative dual-response paradigm used by Allan (1975a), an order response must also be given after $R_{si}$. In this situation, it seems only plausible for these models to assume that the available information in the order center is evaluated.

## Availability of data and materials

Data sets are available via the Open Science Framework at https://osf.io/ec2z3/.

## Declarations

### Open practices

The present study does not include any original experiment for which we could provide data, materials, or preregistrations.
