# Supplementary material for: Independent-channels models of temporal-order judgment revisited: A model comparison
Source: Atten Percept Psychophys. 2024 Aug 6;86(6):2187–209. doi: 10.3758/s13414-024-02915-5 (PMC11410913; doi:10.3758/s13414-024-02915-5)
Supplement: Supplementary file 1 — (pdf 5668 KB) [file 13414_2024_2915_MOESM1_ESM.pdf]

Supplementary material for:

Independent-channels models of temporal-order judgment revisited: A model comparison

Paul Kelber and Rolf Ulrich
University of Tübingen

Contents

Tabular representation of the data **3**
Representation by response proportion 3
Representation by response frequency 13

Graphical representation of data and model fits **23**
Models assuming normally distributed arrival-latency differences 23
Representation by three psychometric functions 23
Representation by two psychometric functions 36
Models assuming Laplace-distributed arrival-latency differences 49
Representation by three psychometric functions 49
Representation by two psychometric functions 63

Tabular representation of other model comparison results **77**
Parameter values for models with Laplace-distributed arrival-latency differences . 77
Selection among models with different response-error and -bias parameters 80
Non-monotonicity indices of observed and fitted psychometric functions 81

Descriptions of the experiments **82**

Tabular representation of the data

Representation by response proportion

Tables S1–10 include the response proportions observed in the eight studies considered in the model comparison.

Table S1

Response proportions in the study by Benussi (1913)

Experiment	Response	Stimulus-onset difference d [ms]										
		−150	−120	−90	−60	−30	0	30	60	90	120	150
1	R_{xy}	.016	.032	.032	.120	.032	.052	.312	.648	.864	.936	.960
	R_{si}	.016	.024	.056	.256	.608	.560	.600	.240	.088	.008	.000
	R_{yx}	.984	.944	.920	.640	.352	.196	.096	.088	.056	.064	.040
2	R_{xy}			.084	.092	.124	.182	.448	.676	.836		
	R_{si}			.060	.170	.484	.586	.312	.120	.044		
	R_{yx}			.848	.724	.408	.216	.208	.130	.124		
3	R_{xy}			.085	.190	.220	.257	.470	.745	.865		
	R_{si}			.015	.100	.400	.577	.360	.130	.060		
	R_{yx}			.890	.675	.335	.155	.125	.095	.020		

Note. The values were reported by Benussi (1913, pp. 521–523). We used them to calculate the response frequencies in Table S11. x: left; y: right.

Table S2

Response proportions in the study by Allan (1975a)

Subject	Response	Stimulus-offset difference d [ms]								
		−100	−75	−50	−25	0	25	50	75	100
AJ	$R_{su \cap xy}$	.06	.15	.11	.21	.21	.26	.39	.67	.83
	$R_{si \cap xy}$	.01	.06	.12	.21	.29	.31	.30	.10	.01
	$R_{si \cap yx}$	.01	.02	.13	.18	.26	.20	.13	.06	.02
	$R_{su \cap yx}$	.91	.76	.62	.37	.24	.21	.16	.15	.13
TM	$R_{su \cap xy}$	.00	.07	.13	.13	.08	.13	.31	.66	.89
	$R_{si \cap xy}$	.04	.06	.17	.28	.38	.47	.41	.17	.01
	$R_{si \cap yx}$	.04	.07	.09	.21	.40	.30	.20	.06	.04
	$R_{su \cap yx}$	.91	.79	.58	.36	.14	.08	.06	.09	.05
BP	$R_{su \cap xy}$	.10	.17	.16	.17	.21	.32	.32	.58	.73
	$R_{si \cap xy}$	.04	.12	.16	.25	.36	.26	.27	.23	.06
	$R_{si \cap yx}$	.02	.04	.08	.20	.18	.18	.20	.09	.09
	$R_{su \cap yx}$	.84	.66	.60	.38	.25	.24	.21	.10	.12

Note. The values were reported by Allan (1975a, p. 31). We used them to calculate the response frequencies in Table S12. x: visual; y: auditory.

Table S3

Response proportions in the study by Allan (1975b)

Subject	Response	Stimulus-offset difference d [ms]								
		−100	−75	−50	−25	0	25	50	75	100
NC	$R_{xy \cap certain}$	.000	.000	.000	.021	.116	.188	.510	.854	1.000
	$R_{xy \cap uncertain}$	.000	.000	.000	.096	.374	.520	.334	.125	.000
	$R_{yx \cap uncertain}$	.021	.010	.083	.330	.448	.261	.125	.021	.000
	$R_{yx \cap certain}$	.979	.990	.917	.553	.062	.031	.031	.000	.000
BM	$R_{xy \cap certain}$	.126	.132	.115	.161	.141	.132	.161	.268	.442
	$R_{xy \cap uncertain}$	.109	.114	.267	.369	.435	.425	.470	.428	.251
	$R_{yx \cap uncertain}$	.084	.155	.236	.327	.323	.365	.315	.232	.209
	$R_{yx \cap certain}$	.681	.599	.382	.143	.101	.078	.054	.072	.098
LM	$R_{xy \cap certain}$	.021	.077	.098	.237	.249	.259	.352	.514	.773
	$R_{xy \cap uncertain}$	.014	.049	.141	.216	.362	.468	.437	.289	.163
	$R_{yx \cap uncertain}$	.043	.084	.198	.295	.266	.165	.126	.148	.043
	$R_{yx \cap certain}$	.922	.790	.563	.252	.123	.108	.085	.049	.021
VR	$R_{xy \cap certain}$	.049	.029	.111	.153	.212	.245	.444	.688	.909
	$R_{xy \cap uncertain}$	.034	.047	.118	.277	.382	.503	.410	.220	.063
	$R_{yx \cap uncertain}$	.084	.188	.236	.313	.297	.203	.090	.056	.000
	$R_{yx \cap certain}$	.833	.736	.535	.257	.109	.049	.056	.036	.028
AT	$R_{xy \cap certain}$	.049	.133	.175	.189	.274	.271	.278	.399	.437
	$R_{xy \cap uncertain}$	.152	.266	.315	.328	.339	.382	.340	.342	.267
	$R_{yx \cap uncertain}$	.139	.189	.286	.238	.223	.180	.257	.140	.148
	$R_{yx \cap certain}$	.660	.412	.224	.245	.164	.167	.125	.119	.148
VV	$R_{xy \cap certain}$	.125	.174	.292	.347	.378	.458	.430	.566	.799
	$R_{xy \cap uncertain}$	.021	.027	.104	.097	.192	.167	.188	.119	.034
	$R_{yx \cap uncertain}$	.000	.035	.076	.209	.115	.153	.139	.105	.056
	$R_{yx \cap certain}$	.854	.764	.528	.347	.315	.222	.243	.210	.111

Note. We calculated the values based on the values reported by Allan (1975b, p. 370). We used these values to calculate the response frequencies in Table S13. x: visual; y: auditory.

Table S4

Response proportions in the study by Ulrich (1987)

Subject	Intensity	Response	Stimulus-onset difference d [ms]								
			−100	−75	−50	−25	0	25	50	75	100
UD	High	R_{xy}	.01	.10	.31	.27	.45	.55	.85	.92	.97
		R_{si}	.00	.01	.08	.23	.27	.20	.04	.02	.00
		R_{yx}	.99	.89	.61	.50	.28	.24	.11	.06	.03
	Low	R_{xy}	.02	.11	.09	.09	.16	.20	.26	.40	.56
		R_{si}	.06	.10	.27	.53	.50	.47	.36	.25	.18
		R_{yx}	.92	.79	.64	.38	.34	.32	.37	.35	.27
MP	High	R_{xy}	.02	.08	.12	.15	.14	.22	.52	.74	.89
		R_{si}	.00	.01	.26	.42	.64	.51	.22	.05	.01
		R_{yx}	.98	.91	.62	.43	.22	.27	.26	.21	.10
	Low	R_{xy}	.05	.14	.30	.26	.26	.30	.55	.56	.86
		R_{si}	.02	.10	.22	.43	.57	.47	.29	.25	.05
		R_{yx}	.93	.75	.48	.31	.17	.22	.16	.19	.09
GU	High	R_{xy}	.01	.02	.08	.05	.04	.07	.56	.84	.97
		R_{si}	.00	.00	.19	.70	.88	.82	.29	.01	.00
		R_{yx}	.99	.98	.73	.25	.08	.11	.15	.15	.03
	Low	R_{xy}	.01	.03	.11	.06	.05	.12	.43	.81	.94
		R_{si}	.01	.03	.28	.67	.90	.82	.51	.10	.02
		R_{yx}	.98	.94	.61	.27	.05	.06	.06	.09	.04

Note. The values were reported by Ulrich (1987, p. 232). We used them to calculate the response frequencies in Table S14. x: top; y: bottom.

Table S5

Response proportions in the study by Jaśkowski (1991a, Experiment 3)

Subject	Duration	Response	Stimulus-onset difference d [ms]										
			−75	−60	−45	−30	−15	0	15	30	45	60	75
PJ	Shorter	R_{xy}	.003	.001	.029	.130	.160	.160	.323	.741	.861	1.000	1.000
		R_{si}	.030	.000	.032	.232	.783	.703	.524	.063	.042	.004	.004
		R_{yx}	.972	.993	.939	.641	.058	.138	.160	.190	.111	.003	.003
	Equal	R_{xy}	.029	.029	.058	.231	.069	.029	.100	.354	.721	.972	1.000
		R_{si}	.001	.028	.003	.160	.862	.972	.872	.653	.072	.002	.002
		R_{yx}	.969	.939	.939	.612	.069	.001	.029	.001	.211	.031	.003
	Longer	R_{xy}	.000	.000	.000	.064	.225	.057	.228	.392	.807	.998	.969
		R_{si}	.001	.002	.001	.000	.230	.680	.769	.582	.134	.004	.004
		R_{yx}	.995	.996	.996	.927	.547	.256	.000	.027	.057	.003	.026
PT	Shorter	R_{xy}	.124	.112	.123	.017	.044	.046	.280	.300	.596	.755	.824
		R_{si}	.067	.160	.675	.937	.767	.805	.577	.170	.001	.028	.029
		R_{yx}	.800	.719	.190	.030	.162	.131	.130	.517	.391	.194	.131
	Equal	R_{xy}	.048	.182	.090	.029	.152	.014	.091	.101	.468	.665	.817
		R_{si}	.002	.099	.578	.915	.767	.968	.865	.816	.348	.028	.003
		R_{yx}	.938	.708	.319	.041	.058	.002	.031	.068	.161	.291	.174
	Longer	R_{xy}	.026	.017	.016	.347	.249	.150	.017	.053	.563	.652	.821
		R_{si}	.003	.025	.026	.107	.417	.735	.854	.745	.189	.075	.001
		R_{yx}	.956	.937	.936	.535	.317	.096	.137	.178	.230	.259	.160
WW	Shorter	R_{xy}	.000	.000	.020	.030	.022	.277	.436	.726	.955	.879	.984
		R_{si}	.002	.068	.228	.585	.546	.418	.335	.056	.026	.000	.000
		R_{yx}	.997	.927	.740	.367	.418	.288	.208	.188	.001	.110	.000
	Equal	R_{xy}	.000	.019	.050	.230	.022	.000	.087	.506	.826	.956	.986
		R_{si}	.000	.000	.057	.305	.805	.933	.805	.444	.124	.000	.000
		R_{yx}	.998	.969	.869	.448	.158	.060	.098	.033	.031	.027	.001
	Longer	R_{xy}	.000	.000	.020	.245	.276	.176	.195	.435	.784	.855	.985
		R_{si}	.024	.000	.000	.024	.154	.452	.650	.512	.194	.055	.000
		R_{yx}	.964	.993	.965	.706	.545	.347	.137	.031	.000	.054	.001

Note. We read the values from a figure (Jaśkowski, 1991a, p. 721). As described in the manuscript, this led to minimal deviations from the observed data. We used these values to calculate the response frequencies in Table S15. x: bottom; y: top.

Table S6

Response proportions in the study by van Eijk et al. (2008) with simple stimuli

Subject	Response	Stimulus-onset difference d [ms]														
		−350	−300	−250	−200	−150	−100	−50	0	50	100	150	200	250	300	350
1	R_{xy}	.000	.000	.000	.000	.000	.000	.000	.000	.033	.150	.550	.667	.850	.883	.950
	R_{si}	.000	.000	.033	.000	.050	.167	.650	.967	.933	.800	.417	.283	.117	.067	.033
	R_{yx}	1.000	1.000	.967	1.000	.950	.833	.350	.033	.033	.050	.033	.050	.033	.050	.017
2	R_{xy}	.017	.033	.017	.017	.017	.017	.017	.033	.100	.300	.517	.867	.883	.850	.900
	R_{si}	.000	.083	.067	.067	.317	.633	.900	.933	.900	.633	.417	.067	.067	.067	.067
	R_{yx}	.983	.883	.917	.917	.667	.350	.083	.033	.000	.067	.067	.067	.050	.083	.033
3	R_{xy}	.000	.000	.000	.000	.050	.033	.000	.000	.017	.117	.383	.567	.900	.817	.967
	R_{si}	.000	.000	.000	.000	.017	.067	.733	1.000	.983	.883	.600	.433	.100	.183	.033
	R_{yx}	1.000	1.000	1.000	1.000	.933	.900	.267	.000	.000	.000	.017	.000	.000	.000	.000
4	R_{xy}	.017	.017	.000	.017	.033	.000	.033	.050	.067	.200	.533	.783	.917	.967	.983
	R_{si}	.000	.000	.000	.033	.167	.233	.483	.800	.883	.700	.417	.067	.050	.033	.000
	R_{yx}	.983	.983	1.000	.950	.800	.767	.483	.150	.050	.100	.050	.150	.033	.000	.017
6	R_{xy}	.017	.017	.050	.017	.017	.033	.117	.550	.550	.950	.967	1.000	1.000	1.000	.983
	R_{si}	.000	.000	.000	.000	.000	.000	.233	.250	.333	.017	.000	.000	.000	.000	.000
	R_{yx}	.983	.983	.950	.983	.983	.967	.650	.200	.117	.033	.033	.000	.000	.000	.017
7	R_{xy}	.017	.000	.000	.017	.000	.000	.000	.000	.017	.167	.700	.950	.967	1.000	1.000
	R_{si}	.000	.017	.033	.017	.150	.400	.750	.900	.950	.833	.283	.033	.017	.000	.000
	R_{yx}	.983	.983	.967	.967	.850	.600	.250	.100	.033	.000	.017	.017	.017	.000	.000
8	R_{xy}	.033	.017	.017	.100	.083	.017	.000	.000	.000	.217	.683	.833	.933	.950	.950
	R_{si}	.000	.017	.033	.033	.183	.633	.917	1.000	1.000	.783	.300	.150	.000	.000	.017
	R_{yx}	.967	.967	.950	.867	.733	.350	.083	.000	.000	.000	.017	.017	.067	.050	.033
9	R_{xy}	.000	.000	.000	.017	.050	.050	.033	.067	.117	.200	.717	.867	.917	.967	.983
	R_{si}	.000	.000	.000	.017	.100	.233	.633	.650	.783	.717	.250	.117	.033	.000	.000
	R_{yx}	1.000	1.000	1.000	.967	.850	.717	.333	.283	.100	.083	.033	.017	.050	.033	.017
10	R_{xy}	.000	.017	.017	.033	.083	.150	.050	.067	.050	.100	.250	.517	.917	.967	.983
	R_{si}	.000	.000	.000	.033	.167	.433	.867	.917	.933	.883	.717	.350	.067	.000	.000
	R_{yx}	1.000	.983	.983	.933	.750	.417	.083	.017	.017	.017	.033	.133	.017	.033	.017
11	R_{xy}	.150	.133	.117	.117	.067	.033	.017	.067	.183	.567	.700	.900	.917	.983	.967
	R_{si}	.067	.150	.350	.667	.800	.883	.983	.933	.817	.433	.300	.050	.067	.000	.000
	R_{yx}	.783	.717	.533	.217	.133	.083	.000	.000	.000	.000	.000	.050	.017	.017	.033
12	R_{xy}	.017	.000	.017	.050	.000	.000	.033	.000	.017	.083	.317	.717	.850	.967	.967
	R_{si}	.067	.067	.100	.267	.650	.917	.967	.983	.983	.900	.667	.250	.150	.033	.033
	R_{yx}	.917	.933	.883	.683	.350	.083	.000	.017	.000	.017	.017	.033	.000	.000	.000

Note. We calculated the values based on the response frequencies in Table S16 (rounded to three decimal places). x: visual; y: auditory.

Table S7

Response proportions in the study by van Eijk et al. (2008) with complex stimuli

Subject	Response	Stimulus-onset difference d [ms]														
		−350	−300	−250	−200	−150	−100	−50	0	50	100	150	200	250	300	350
1	R_{xy}	.017	.000	.033	.017	.000	.033	.100	.100	.083	.200	.567	.783	.950	1.000	1.000
	R_{si}	.000	.000	.000	.000	.000	.033	.200	.883	.917	.800	.433	.217	.050	.000	.000
	R_{yx}	.983	1.000	.967	.983	1.000	.933	.700	.017	.000	.000	.000	.000	.000	.000	.000
2	R_{xy}	.000	.017	.000	.000	.000	.017	.017	.017	.000	.117	.667	.850	.967	.933	.967
	R_{si}	.000	.000	.017	.033	.050	.100	.583	.900	.950	.850	.283	.133	.033	.050	.017
	R_{yx}	1.000	.983	.983	.967	.950	.883	.400	.083	.050	.033	.050	.017	.000	.017	.017
3	R_{xy}	.000	.000	.017	.000	.050	.033	.000	.017	.000	.033	.233	.650	.817	.983	1.000
	R_{si}	.000	.000	.000	.000	.017	.067	.850	.967	1.000	.967	.767	.350	.183	.017	.000
	R_{yx}	1.000	1.000	.983	1.000	.933	.900	.150	.017	.000	.000	.000	.000	.000	.000	.000
4	R_{xy}	.000	.000	.017	.017	.033	.067	.117	.100	.050	.100	.417	.867	.917	.933	.950
	R_{si}	.017	.017	.000	.033	.000	.100	.400	.883	.933	.900	.550	.133	.083	.050	.050
	R_{yx}	.983	.983	.983	.950	.967	.833	.483	.017	.017	.000	.033	.000	.000	.017	.000
5	R_{xy}	.050	.033	.000	.033	.067	.083	.067	.033	.050	.217	.683	.833	.933	.900	.967
	R_{si}	.000	.017	.033	.033	.067	.433	.833	.933	.917	.750	.283	.150	.017	.067	.017
	R_{yx}	.950	.950	.967	.933	.867	.483	.100	.033	.033	.033	.033	.017	.050	.033	.017
6	R_{xy}	.000	.000	.000	.000	.000	.000	.067	.133	.033	.533	.983	1.000	.983	1.000	1.000
	R_{si}	.000	.000	.000	.000	.000	.000	.100	.833	.967	.467	.017	.000	.000	.000	.000
	R_{yx}	1.000	1.000	1.000	1.000	1.000	1.000	.833	.033	.000	.000	.000	.000	.017	.000	.000
7	R_{xy}	.000	.000	.000	.000	.000	.000	.000	.000	.033	.200	.633	.933	.983	1.000	1.000
	R_{si}	.017	.000	.000	.000	.067	.233	.783	1.000	.967	.800	.367	.050	.017	.000	.000
	R_{yx}	.983	1.000	1.000	1.000	.933	.767	.217	.000	.000	.000	.000	.017	.000	.000	.000
8	R_{xy}	.050	.033	.033	.117	.083	.183	.050	.017	.017	.250	.850	.917	.967	.983	.967
	R_{si}	.000	.000	.000	.017	.017	.317	.933	.983	.983	.750	.117	.017	.017	.000	.000
	R_{yx}	.950	.967	.967	.867	.900	.500	.017	.000	.000	.000	.033	.067	.017	.017	.033
9	R_{xy}	.017	.000	.033	.033	.000	.033	.033	.067	.200	.217	.700	.817	.950	.950	.950
	R_{si}	.000	.017	.017	.017	.067	.217	.717	.817	.750	.700	.217	.083	.017	.017	.033
	R_{yx}	.983	.983	.950	.950	.933	.750	.250	.117	.050	.083	.083	.100	.033	.033	.017
10	R_{xy}	.000	.000	.000	.000	.000	.000	.017	.017	.000	.067	.550	.967	.967	1.000	.983
	R_{si}	.000	.000	.017	.000	.000	.000	.233	.967	.983	.917	.400	.033	.033	.000	.017
	R_{yx}	1.000	1.000	.983	1.000	1.000	1.000	.750	.017	.017	.017	.050	.000	.000	.000	.000
11	R_{xy}	.033	.050	.033	.017	.000	.000	.000	.000	.000	.017	.250	.683	.817	.933	.967
	R_{si}	.083	.183	.250	.500	.667	.917	1.000	.983	1.000	.967	.717	.300	.133	.050	.000
	R_{yx}	.883	.767	.717	.483	.333	.083	.000	.017	.000	.017	.033	.017	.050	.017	.033
12	R_{xy}	.017	.000	.000	.050	.050	.067	.100	.100	.167	.417	.700	.900	.950	1.000	1.000
	R_{si}	.000	.000	.033	.033	.133	.483	.733	.850	.817	.567	.300	.050	.033	.000	.000
	R_{yx}	.983	1.000	.967	.917	.817	.450	.167	.050	.017	.017	.000	.050	.017	.000	.000

Note. We calculated the values based on the response frequencies in Table S17 (rounded to three decimal places). x: visual; y: auditory.

Table S8

Response proportions from the subjects 1–10 in the study by García-Pérez and Alcalá-Quintana (2018)

Subject	Response	Stimulus-onset difference d [ms]																						
		−200	−183	−167	−150	−133	−117	−100	−83	−67	−50	−33	−17	0	17	33	50	67	83	100	117	133	150	167
1	R_{xy}							.000	.000	.000	.016	.016	.040	.065	.109	.044	.296	.625	.825	.968	1.000			
	R_{si}							.000	.171	.404	.475	.758	.860	.806	.783	.822	.500	.188	.175	.032	.000			
	R_{su}							.000	.000	.106	.131	.129	.100	.129	.109	.133	.204	.188	.000	.000	.000			
	R_{yx}							1.000	.829	.489	.377	.097	.000	.000	.000	.000	.000	.000	.000	.000	.000			
2	R_{xy}			.000	.000	.333	.000	.059	.083	.171	.070	.075	.080	.000	.026	.075	.392	.655	.860	.872	1.000	1.000		
	R_{si}			.000	.000	.000	.000	.000	.000	.000	.281	.717	.880	1.000	.895	.875	.490	.145	.020	.000	.000	.000		
	R_{su}			.000	.000	.000	.000	.029	.000	.000	.000	.000	.040	.000	.000	.025	.000	.000	.000	.000	.000	.000		
	R_{yx}			1.000	1.000	.667	1.000	.912	.917	.829	.649	.208	.000	.000	.079	.025	.118	.200	.120	.128	.000	.000		
3	R_{xy}							.000	.000	.000	.000	.000	.000	.000	.000	.087	.323	.604	.838	1.000				
	R_{si}							.000	.000	.073	.368	.859	.854	.971	.978	.761	.548	.245	.000	.000				
	R_{su}							.000	.000	.098	.074	.031	.042	.000	.022	.130	.081	.151	.162	.000				
	R_{yx}							1.000	1.000	.829	.559	.109	.104	.029	.000	.022	.048	.000	.000	.000				
4	R_{xy}							.000	.000	.000	.000	.048	.000	.000	.000	.111	.377	.761	.938	1.000				
	R_{si}							.000	.000	.048	.356	.683	.962	1.000	.981	.815	.393	.065	.000	.000				
	R_{su}							.000	.000	.000	.119	.048	.019	.000	.000	.056	.180	.174	.062	.000				
	R_{yx}							1.000	1.000	.952	.525	.222	.019	.000	.019	.019	.049	.000	.000	.000				
5	R_{xy}						.000	.000	.000	.000	.000	.035	.000	.000	.000	.067	.455	.690	.879	.929	1.000	1.000		
	R_{si}						.000	.000	.000	.000	.122	.404	.611	.639	.750	.517	.255	.000	.000	.000	.000	.000		
	R_{su}						.000	.036	.133	.150	.327	.404	.315	.278	.250	.417	.291	.286	.091	.036	.000	.000		
	R_{yx}						1.000	.964	.867	.850	.551	.158	.074	.083	.000	.000	.000	.024	.030	.036	.000	.000		
6	R_{xy}	.000	.000	.333	.000	.143	.120	.161	.193	.321	.182	.100	.048	.043	.111	.093	.224	.528	.897	.963	1.000			
	R_{si}	.000	.000	.000	.000	.000	.000	.032	.088	.245	.727	.800	.952	.957	.889	.907	.724	.472	.051	.000	.000			
	R_{su}	.000	.000	.000	.000	.000	.000	.000	.000	.000	.000	.000	.000	.000	.000	.000	.034	.000	.026	.037	.000			
	R_{yx}	1.000	1.000	.667	1.000	.857	.880	.806	.719	.434	.091	.100	.000	.000	.000	.000	.017	.000	.026	.000	.000			
7	R_{xy}					.000	.000	.032	.028	.102	.057	.042	.109	.111	.119	.125	.300	.659	.833	.889	.889	.833	1.000	1.000
	R_{si}					.000	.000	.000	.000	.122	.377	.583	.696	.806	.810	.792	.600	.227	.056	.000	.000	.000	.000	.000
	R_{su}					.000	.000	.000	.000	.000	.000	.000	.000	.000	.000	.000	.000	.000	.000	.000	.000	.000	.000	.000
	R_{yx}					1.000	1.000	.968	.972	.776	.566	.375	.196	.083	.071	.083	.100	.114	.111	.111	.111	.167	.000	.000
8	R_{xy}			.000	.000	.200	.000	.103	.059	.119	.098	.028	.065	.030	.023	.250	.462	.614	.917	.968	1.000	1.000		
	R_{si}			.000	.000	.000	.000	.000	.118	.322	.549	.917	.806	.939	.977	.750	.500	.295	.028	.000	.000	.000		
	R_{su}			.000	.000	.000	.000	.000	.000	.000	.000	.000	.000	.000	.000	.000	.000	.000	.000	.000	.000	.000		
	R_{yx}			1.000	1.000	.800	1.000	.897	.824	.559	.353	.056	.129	.030	.000	.000	.038	.091	.056	.032	.000	.000		
9	R_{xy}					.000	.000	.026	.085	.127	.019	.000	.000	.000	.000	.054	.263	.610	.830	.953	1.000	1.000		
	R_{si}					.000	.000	.026	.064	.255	.574	.850	.969	1.000	.960	.892	.526	.136	.038	.000	.000	.000		
	R_{su}					.000	.000	.000	.106	.073	.056	.050	.000	.000	.040	.000	.070	.051	.075	.000	.000	.000		
	R_{yx}					1.000	1.000	.947	.745	.545	.352	.100	.031	.000	.000	.054	.140	.203	.057	.047	.000	.000		
10	R_{xy}					.000	.000	.031	.027	.077	.081	.040	.075	.074	083	.234	.623	.805	.935	.962	1.000			
	R_{si}					.000	.000	.031	.000	.038	.274	.580	.700	.852	.792	.562	.213	.073	.032	.000	.000			
	R_{su}					.000	.333	.000	.027	.058	.161	.280	.200	.074	.125	.188	.148	.122	.032	.038	.000			
	R_{yx}					1.000	.667	.938	.946	.827	.484	.100	.025	.000	.000	.016	.016	.000	.000	.000	.000			

Note. We calculated the values based on the response frequencies in Table S18 (rounded to three decimal places). x: right; y: left.

Table S9

Response proportions from the subjects 11–19 in the study by García-Pérez and Alcalá-Quintana (2018)

Subject	Response	Stimulus-onset difference d [ms]																						
		−200	−183	−167	−150	−133	−117	−100	−83	−67	−50	−33	−17	0	17	33	50	67	83	100	117	133	150	167
11	R_{xy}						.000	.000	.022	.061	.373	.140	.212	.333	.385	.429	.615	.770	.930	.976	1.000	1.000		
	R_{si}						.000	.000	.000	.000	.000	.000	.000	.000	.000	.000	.000	.000	.000	.000	.000	.000		
	R_{su}						.000	.000	.000	.000	.039	.140	.273	.074	.269	.071	.019	.014	.000	.000	.000	.000		
	R_{yx}						1.000	1.000	.978	.939	.588	.721	.515	.593	.346	.500	.365	.216	.070	.024	.000	.000		
12	R_{xy}					.000	.000	.043	.204	.161	.121	.094	.062	.000	.095	.129	.500	.604	.776	.815	.900	.846	1.000	1.000
	R_{si}					.000	.000	.022	.019	.145	.448	.750	.812	1.000	.810	.710	.325	.083	.061	.000	.000	.077	.000	.000
	R_{su}					.000	.000	.000	.000	.065	.069	.094	.125	.000	.095	.129	.075	.042	.000	.000	.000	.000	.000	.000
	R_{yx}					1.000	1.000	.935	.778	.629	.362	.062	.000	.000	.000	.032	.100	.271	.163	.185	.100	.077	.000	.000
13	R_{xy}						.000	.000	.048	.123	.117	.182	.107	.200	.077	.184	.163	.564	.717	.851	1.000	.800	1.000	1.000
	R_{si}						.000	.032	.024	.105	.350	.568	.714	.640	.731	.579	.694	.309	.170	.064	.000	.000	.000	.000
	R_{su}						.000	.000	.000	.035	.000	.000	.000	.000	.000	.026	.000	.000	.000	.000	.000	.000	.000	.000
	R_{yx}						1.000	.968	.929	.737	.533	.250	.179	.160	.192	.211	.143	.127	.113	.085	.000	.200	.000	.000
14	R_{xy}			.000	.000	.067	.048	.194	.067	.137	.029	.038	.000	.000	.000	.000	.088	.333	.582	.667	.667	.750		
	R_{si}			.000	.000	.067	.238	.177	.400	.549	.743	.923	.917	1.000	1.000	.912	.860	.565	.309	.179	.222	.250		
	R_{su}			.000	.000	.000	.000	.065	.033	.078	.114	.038	.000	.000	.000	.029	.053	.072	.073	.051	.111	.000		
	R_{yx}			1.000	1.000	.867	.714	.565	.500	.235	.114	.000	.083	.000	.000	.059	.000	.029	.036	.103	.000	.000		
15	R_{xy}					.000	.000	.093	.106	.155	.125	.045	.000	.000	.000	.074	.507	.816	.968	.963	1.000			
	R_{si}					.000	.000	.000	.000	.086	.375	.795	.960	.944	.969	.778	.333	.061	.000	.000	.000			
	R_{su}					.000	.000	.047	.000	.086	.172	.068	.040	.056	.031	.130	.116	.102	.000	.037	.000			
	R_{yx}					1.000	1.000	.860	.894	.672	.328	.091	.000	.000	.000	.019	.043	.020	.032	.000	.000			
16	R_{xy}							.000	.000	.000	.000	.091	.197	.275	.517	.552	.865	.974	1.000	1.000				
	R_{si}							.000	.000	.000	.000	.127	.268	.290	.150	.155	.019	.000	.000	.000				
	R_{su}							.000	.000	.000	.047	.073	.113	.087	.133	.069	.019	.000	.000	.000				
	R_{yx}							1.000	1.000	1.000	.953	.709	.423	.348	.200	.224	.096	.026	.000	.000				
17	R_{xy}					.000	.000	.057	.040	.052	.155	.104	.056	.111	.083	.150	.339	.784	.811	.903	.800	1.000		
	R_{si}					.000	.000	.029	.040	.069	.431	.479	.639	.556	.667	.650	.375	.176	.027	.000	.000	.000		
	R_{su}					.000	.000	.057	.080	.190	.190	.188	.278	.333	.194	.175	.232	.039	.081	.065	.200	.000		
	R_{yx}					1.000	1.000	.857	.840	.690	.224	.229	.028	.000	.056	.025	.054	.000	.081	.032	.000	.000		
18	R_{xy}							.000	.000	.070	.133	.180	.051	.100	.062	.196	.547	.732	.972	1.000	1.000			
	R_{si}							.036	.000	.158	.267	.580	.821	.900	.812	.667	.344	.161	.000	.000	.000			
	R_{su}							.000	.000	.000	.000	.000	.000	.000	.000	.000	.000	.000	.000	.000	.000			
	R_{yx}							.964	1.000	.772	.600	.240	.128	.000	.125	.137	.109	.107	.028	.000	.000			
19	R_{xy}						.000	.000	.083	.103	.000	.032	.027	.000	.000	.038	.114	.294	.556	.820	.950	.750	1.000	1.000
	R_{si}						.000	.075	.146	.362	.755	.903	.919	1.000	1.000	.885	.829	.549	.190	.060	.000	.000	.000	.000
	R_{su}						.000	.000	.083	.034	.061	.032	.054	.000	.000	.038	.000	.098	.032	.020	.000	.000	.000	.000
	R_{yx}						1.000	.925	.688	.500	.184	.032	.000	.000	.000	.038	.057	.059	.222	.100	.050	.250	.000	.000

Note. We calculated the values based on the response frequencies in Table S19 (rounded to three decimal places). x: right; y: left.

Table S10

Response proportions in the study by Lahkar et al. (2023)

Response	Stimulus-onset difference d [ms]																										
	−65	−60	−55	−50	−45	−40	−35	−30	−25	−20	−15	−10	−5	0	5	10	15	20	25	30	35	40	45	50	55	60	65
R_{xy}	.004	.017	.013	.017	.017	.033	.033	.017	.017	.033	.008	.004	.008	.019	.038	.067	.150	.300	.475	.637	.671	.787	.900	.925	.938	.967	.983
R_{si}	.000	.013	.017	.079	.092	.158	.208	.392	.571	.679	.854	.917	.954	.952	.954	.921	.825	.667	.500	.342	.300	.192	.088	.067	.050	.029	.004
R_{yx}	.996	.971	.971	.904	.892	.808	.758	.592	.412	.288	.138	.079	.038	.029	.008	.013	.025	.033	.025	.021	.029	.021	.013	.008	.013	.004	.013

Note. We calculated the values based on the response frequencies in Table S20 (rounded to three decimal places). x: right; y: left.

Representation by response frequency

Tables S11–20 include the response frequencies observed in the eight studies considered in the model comparison.

Table S11

Response frequencies in the study by Benussi (1913)

Experiment	Response	Stimulus-onset difference d [ms]										
		−150	−120	−90	−60	−30	0	30	60	90	120	150
1	R_{xy}	2	4	4	15	4	13	40	83	111	120	123
	R_{si}	2	3	7	33	78	143	77	31	11	1	0
	R_{yx}	126	121	118	82	45	50	12	11	7	8	5
2	R_{xy}			18	20	26	47	95	144	178		
	R_{si}			13	36	103	150	66	26	9		
	R_{yx}			181	154	87	55	44	28	26		
3	R_{xy}			18	40	47	66	100	159	184		
	R_{si}			3	21	85	148	77	28	13		
	R_{yx}			190	144	71	40	27	20	4		

Note. We calculated the values based on the response proportions in Table S1 (rounded to whole numbers). x: left; y: right.

Table S12

Response frequencies in the study by Allan (1975a)

Subject	Response	Stimulus-offset difference d [ms]								
		−100	−75	−50	−25	0	25	50	75	100
AJ	$R_{su\cap xy}$	6	14	11	20	81	25	37	64	80
	$R_{si\cap xy}$	1	6	12	20	111	30	29	10	1
	$R_{si\cap yx}$	1	2	12	17	100	19	12	6	2
	$R_{su\cap yx}$	87	73	60	36	92	20	15	14	12
TM	$R_{su\cap xy}$	0	7	12	12	31	12	30	63	85
	$R_{si\cap xy}$	4	6	16	27	146	45	39	16	1
	$R_{si\cap yx}$	4	7	9	20	154	29	19	6	4
	$R_{su\cap yx}$	87	76	56	35	54	8	6	9	5
BP	$R_{su\cap xy}$	12	20	19	20	101	38	38	70	88
	$R_{si\cap xy}$	5	14	19	30	173	31	32	28	7
	$R_{si\cap yx}$	2	5	10	24	86	22	24	11	11
	$R_{su\cap yx}$	101	79	72	46	120	29	25	12	14

Note. We calculated the values based on the response proportions in Table S2 (rounded to whole numbers). x: visual; y: auditory.

Table S13

Response frequencies in the study by Allan (1975b)

Subject	Response	Stimulus-offset difference d [ms]								
		−100	−75	−50	−25	0	25	50	75	100
NC	$R_{xy \cap certain}$	0	0	0	2	45	18	49	82	96
	$R_{xy \cap uncertain}$	0	0	0	9	144	50	32	12	0
	$R_{yx \cap uncertain}$	2	1	8	32	172	25	12	2	0
	$R_{yx \cap certain}$	94	95	88	53	24	3	3	0	0
BM	$R_{xy \cap certain}$	18	19	17	23	81	19	23	39	64
	$R_{xy \cap uncertain}$	16	16	38	53	251	61	68	62	36
	$R_{yx \cap uncertain}$	12	22	34	47	186	53	45	33	30
	$R_{yx \cap certain}$	98	86	55	21	58	11	8	10	14
LM	$R_{xy \cap certain}$	3	11	14	34	143	37	51	74	111
	$R_{xy \cap uncertain}$	2	7	20	31	209	67	63	42	23
	$R_{yx \cap uncertain}$	6	12	29	42	153	24	18	21	6
	$R_{yx \cap certain}$	133	114	81	36	71	16	12	7	3
VR	$R_{xy \cap certain}$	7	4	16	22	122	35	64	99	131
	$R_{xy \cap uncertain}$	5	7	17	40	220	72	59	32	9
	$R_{yx \cap uncertain}$	12	27	34	45	171	29	13	8	0
	$R_{yx \cap certain}$	120	106	77	37	63	7	8	5	4
AT	$R_{xy \cap certain}$	8	22	29	32	184	46	47	67	73
	$R_{xy \cap uncertain}$	26	45	53	55	228	64	57	57	45
	$R_{yx \cap uncertain}$	23	32	48	40	150	30	43	24	25
	$R_{yx \cap certain}$	111	69	38	41	110	28	21	20	25
VV	$R_{xy \cap certain}$	18	25	42	50	218	66	62	82	115
	$R_{xy \cap uncertain}$	3	4	15	14	111	24	27	17	5
	$R_{yx \cap uncertain}$	0	5	11	30	66	22	20	15	8
	$R_{yx \cap certain}$	123	110	76	50	181	32	35	30	16

Note. We calculated the values based on the response proportions in Table S3 (rounded to whole numbers). x: visual; y: auditory.

Table S14

Response frequencies in the study by Ulrich (1987)

Subject	Intensity	Response	Stimulus-onset difference d [ms]								
			−100	−75	−50	−25	0	25	50	75	100
UD	High	R_{xy}	1	10	31	27	180	55	85	92	97
		R_{si}	0	1	8	23	108	20	4	2	0
		R_{yx}	99	89	61	50	112	24	11	6	3
	Low	R_{xy}	2	11	9	9	64	20	26	40	56
		R_{si}	6	10	27	53	200	47	36	25	18
		R_{yx}	92	79	64	38	136	32	37	35	27
MP	High	R_{xy}	2	8	12	15	56	22	52	74	89
		R_{si}	0	1	26	42	256	51	22	5	1
		R_{yx}	98	91	62	43	88	27	26	21	10
	Low	R_{xy}	5	14	30	26	104	30	55	56	86
		R_{si}	2	10	22	43	228	47	29	25	5
		R_{yx}	93	75	48	31	68	22	16	19	9
GU	High	R_{xy}	1	2	8	5	16	7	56	84	97
		R_{si}	0	0	19	70	352	82	29	1	0
		R_{yx}	99	98	73	25	32	11	15	15	3
	Low	R_{xy}	1	3	11	6	20	12	43	81	94
		R_{si}	1	3	28	67	360	82	51	10	2
		R_{yx}	98	94	61	27	20	6	6	9	4

Note. We calculated the values based on the response proportions in Table S4 (rounded to whole numbers). x: top; y: bottom.

Table S15

Response frequencies in the study by Jaśkowski (1991a, Experiment 3)

Subject	Duration	Response	Stimulus-onset difference d [ms]										
			−75	−60	−45	−30	−15	0	15	30	45	60	75
PJ	Shorter	R_{xy}	0	0	3	12	14	14	29	67	77	90	90
		R_{si}	3	0	3	21	70	63	47	6	4	0	0
		R_{yx}	87	89	85	58	5	12	14	17	10	0	0
	Equal	R_{xy}	3	3	5	21	6	3	9	32	65	87	90
		R_{si}	0	3	0	14	78	87	78	59	6	0	0
		R_{yx}	87	85	85	55	6	0	3	0	19	3	0
	Longer	R_{xy}	0	0	0	6	20	5	21	35	73	90	87
		R_{si}	0	0	0	0	21	61	69	52	12	0	0
		R_{yx}	90	90	90	83	49	23	0	2	5	0	2
PT	Shorter	R_{xy}	11	10	11	2	4	4	25	27	54	68	74
		R_{si}	6	14	61	84	69	72	52	15	0	3	3
		R_{yx}	72	65	17	3	15	12	12	47	35	17	12
	Equal	R_{xy}	4	16	8	3	14	1	8	9	42	60	74
		R_{si}	0	9	52	82	69	87	78	73	31	3	0
		R_{yx}	84	64	29	4	5	0	3	6	14	26	16
	Longer	R_{xy}	2	2	1	31	22	14	2	5	51	59	74
		R_{si}	0	2	2	10	38	66	77	67	17	7	0
		R_{yx}	86	84	84	48	29	9	12	16	21	23	14
WW	Shorter	R_{xy}	0	0	2	3	2	25	39	65	86	79	89
		R_{si}	0	6	21	53	49	38	30	5	2	0	0
		R_{yx}	90	83	67	33	38	26	19	17	0	10	0
	Equal	R_{xy}	0	2	4	21	2	0	8	46	74	86	89
		R_{si}	0	0	5	27	72	84	72	40	11	0	0
		R_{yx}	90	87	78	40	14	5	9	3	3	2	0
	Longer	R_{xy}	0	0	2	22	25	16	18	39	71	77	89
		R_{si}	2	0	0	2	14	41	58	46	17	5	0
		R_{yx}	87	89	87	64	49	31	12	3	0	5	0

Note. We calculated the values based on the response proportions in Table S5 (rounded to whole numbers). x: bottom; y: top.

Table S16

Response frequencies in the study by van Eijk et al. (2008) with simple stimuli

Subject	Response	Stimulus-onset difference d [ms]														
		−350	−300	−250	−200	−150	−100	−50	0	50	100	150	200	250	300	350
1	R_{xy}	0	0	0	0	0	0	0	0	2	9	33	40	51	53	57
	R_{si}	0	0	2	0	3	10	39	58	56	48	25	17	7	4	2
	R_{yx}	60	60	58	60	57	50	21	2	2	3	2	3	2	3	1
2	R_{xy}	1	2	1	1	1	1	1	2	6	18	31	52	53	51	54
	R_{si}	0	5	4	4	19	38	54	56	54	38	25	4	4	4	4
	R_{yx}	59	53	55	55	40	21	5	2	0	4	4	4	3	5	2
3	R_{xy}	0	0	0	0	3	2	0	0	1	7	23	34	54	49	58
	R_{si}	0	0	0	0	1	4	44	60	59	53	36	26	6	11	2
	R_{yx}	60	60	60	60	56	54	16	0	0	0	1	0	0	0	0
4	R_{xy}	1	1	0	1	2	0	2	3	4	12	32	47	55	58	59
	R_{si}	0	0	0	2	10	14	29	48	53	42	25	4	3	2	0
	R_{yx}	59	59	60	57	48	46	29	9	3	6	3	9	2	0	1
6	R_{xy}	1	1	3	1	1	2	7	33	33	57	58	60	60	60	59
	R_{si}	0	0	0	0	0	0	14	15	20	1	0	0	0	0	0
	R_{yx}	59	59	57	59	59	58	39	12	7	2	2	0	0	0	1
7	R_{xy}	1	0	0	1	0	0	0	0	1	10	42	57	58	60	60
	R_{si}	0	1	2	1	9	24	45	54	57	50	17	2	1	0	0
	R_{yx}	59	59	58	58	51	36	15	6	2	0	1	1	1	0	0
8	R_{xy}	2	1	1	6	5	1	0	0	0	13	41	50	56	57	57
	R_{si}	0	1	2	2	11	38	55	60	60	47	18	9	0	0	1
	R_{yx}	58	58	57	52	44	21	5	0	0	0	1	1	4	3	2
9	R_{xy}	0	0	0	1	3	3	2	4	7	12	43	52	55	58	59
	R_{si}	0	0	0	1	6	14	38	39	47	43	15	7	2	0	0
	R_{yx}	60	60	60	58	51	43	20	17	6	5	2	1	3	2	1
10	R_{xy}	0	1	1	2	5	9	3	4	3	6	15	31	55	58	59
	R_{si}	0	0	0	2	10	26	52	55	56	53	43	21	4	0	0
	R_{yx}	60	59	59	56	45	25	5	1	1	1	2	8	1	2	1
11	R_{xy}	9	8	7	7	4	2	1	4	11	34	42	54	55	59	58
	R_{si}	4	9	21	40	48	53	59	56	49	26	18	3	4	0	0
	R_{yx}	47	43	32	13	8	5	0	0	0	0	0	3	1	1	2
12	R_{xy}	1	0	1	3	0	0	2	0	1	5	19	43	51	58	58
	R_{si}	4	4	6	16	39	55	58	59	59	54	40	15	9	2	2
	R_{yx}	55	56	53	41	21	5	0	1	0	1	1	2	0	0	0

Note. The values were provided to us by van Eijk et al. (2008). We used these values to calculate the response proportions in Table S6. x: visual; y: auditory.

Table S17

Response frequencies in the study by van Eijk et al. (2008) with complex stimuli

Subject	Response	Stimulus-onset difference d [ms]														
		−350	−300	−250	−200	−150	−100	−50	0	50	100	150	200	250	300	350
1	R_{xy}	1	0	2	1	0	2	6	6	5	12	34	47	57	60	60
	R_{si}	0	0	0	0	0	2	12	53	55	48	26	13	3	0	0
	R_{yx}	59	60	58	59	60	56	42	1	0	0	0	0	0	0	0
2	R_{xy}	0	1	0	0	0	1	1	1	0	7	40	51	58	56	58
	R_{si}	0	0	1	2	3	6	35	54	57	51	17	8	2	3	1
	R_{yx}	60	59	59	58	57	53	24	5	3	2	3	1	0	1	1
3	R_{xy}	0	0	1	0	3	2	0	1	0	2	14	39	49	59	60
	R_{si}	0	0	0	0	1	4	51	58	60	58	46	21	11	1	0
	R_{yx}	60	60	59	60	56	54	9	1	0	0	0	0	0	0	0
4	R_{xy}	0	0	1	1	2	4	7	6	3	6	25	52	55	56	57
	R_{si}	1	1	0	2	0	6	24	53	56	54	33	8	5	3	3
	R_{yx}	59	59	59	57	58	50	29	1	1	0	2	0	0	1	0
5	R_{xy}	3	2	0	2	4	5	4	2	3	13	41	50	56	54	58
	R_{si}	0	1	2	2	4	26	50	56	55	45	17	9	1	4	1
	R_{yx}	57	57	58	56	52	29	6	2	2	2	2	1	3	2	1
6	R_{xy}	0	0	0	0	0	0	4	8	2	32	59	60	59	60	60
	R_{si}	0	0	0	0	0	0	6	50	58	28	1	0	0	0	0
	R_{yx}	60	60	60	60	60	60	50	2	0	0	0	0	1	0	0
7	R_{xy}	0	0	0	0	0	0	0	0	2	12	38	56	59	60	60
	R_{si}	1	0	0	0	4	14	47	60	58	48	22	3	1	0	0
	R_{yx}	59	60	60	60	56	46	13	0	0	0	0	1	0	0	0
8	R_{xy}	3	2	2	7	5	11	3	1	1	15	51	55	58	59	58
	R_{si}	0	0	0	1	1	19	56	59	59	45	7	1	1	0	0
	R_{yx}	57	58	58	52	54	30	1	0	0	0	2	4	1	1	2
9	R_{xy}	1	0	2	2	0	2	2	4	12	13	42	49	57	57	57
	R_{si}	0	1	1	1	4	13	43	49	45	42	13	5	1	1	2
	R_{yx}	59	59	57	57	56	45	15	7	3	5	5	6	2	2	1
10	R_{xy}	0	0	0	0	0	0	1	1	0	4	33	58	58	60	59
	R_{si}	0	0	1	0	0	0	14	58	59	55	24	2	2	0	1
	R_{yx}	60	60	59	60	60	60	45	1	1	1	3	0	0	0	0
11	R_{xy}	2	3	2	1	0	0	0	0	0	1	15	41	49	56	58
	R_{si}	5	11	15	30	40	55	60	59	60	58	43	18	8	3	0
	R_{yx}	53	46	43	29	20	5	0	1	0	1	2	1	3	1	2
12	R_{xy}	1	0	0	3	3	4	6	6	10	25	42	54	57	60	60
	R_{si}	0	0	2	2	8	29	44	51	49	34	18	3	2	0	0
	R_{yx}	59	60	58	55	49	27	10	3	1	1	0	3	1	0	0

Note. The values were provided to us by van Eijk et al. (2008). We used these values to calculate the response proportions in Table S7. x: visual; y: auditory.

Table S18

Response frequencies from the subjects 1–10 in the study by García-Pérez and Alcalá-Quintana (2018)

Subject	Response	Stimulus-onset difference d [ms]																						
		−200	−183	−167	−150	−133	−117	−100	−83	−67	−50	−33	−17	0	17	33	50	67	83	100	117	133	150	167
1	R_{xy}							0	0	0	1	1	2	2	5	2	16	30	33	30	1			
	R_{si}							0	6	19	29	47	43	25	36	37	27	9	7	1	0			
	R_{su}							0	0	5	8	8	5	4	5	6	11	9	0	0	0			
	R_{yx}							25	29	23	23	6	0	0	0	0	0	0	0	0	0			
2	R_{xy}			0	0	1	0	2	3	7	4	4	2	0	1	3	20	36	43	41	12	6		
	R_{si}			0	0	0	0	0	0	0	16	38	22	21	34	35	25	8	1	0	0	0		
	R_{su}			0	0	0	0	1	0	0	0	0	1	0	0	1	0	0	0	0	0	0		
	R_{yx}			1	1	2	5	31	33	34	37	11	0	0	3	1	6	11	6	6	0	0		
3	R_{xy}							0	0	0	0	0	0	0	0	4	20	32	31	27				
	R_{si}							0	0	3	25	55	41	33	44	35	34	13	0	0				
	R_{su}							0	0	4	5	2	2	0	1	6	5	8	6	0				
	R_{yx}							24	27	34	38	7	5	1	0	1	3	0	0	0				
4	R_{xy}							0	0	0	0	3	0	0	0	6	23	35	30	24				
	R_{si}							0	0	2	21	43	51	39	52	44	24	3	0	0				
	R_{su}							0	0	0	7	3	1	0	0	3	11	8	2	0				
	R_{yx}							24	26	40	31	14	1	0	1	1	3	0	0	0				
5	R_{xy}						0	0	0	0	0	2	0	0	0	4	25	29	29	26	2	1		
	R_{si}						0	0	0	0	6	23	33	23	45	31	14	0	0	0	0	0		
	R_{su}						0	1	4	6	16	23	17	10	15	25	16	12	3	1	0	0		
	R_{yx}						1	27	26	34	27	9	4	3	0	0	0	1	1	1	0	0		
6	R_{xy}	0	0	1	0	2	3	10	11	17	6	2	1	1	4	4	13	28	35	26	2			
	R_{si}	0	0	0	0	0	0	2	5	13	24	16	20	22	32	39	42	25	2	0	0			
	R_{su}	0	0	0	0	0	0	0	0	0	0	0	0	0	0	0	2	0	1	1	0			
	R_{yx}	1	1	2	5	12	22	50	41	23	3	2	0	0	0	0	1	0	1	0	0			
7	R_{xy}					0	0	1	1	5	3	2	5	4	5	6	15	29	30	32	8	5	2	1
	R_{si}					0	0	0	0	6	20	28	32	29	34	38	30	10	2	0	0	0	0	0
	R_{su}					0	0	0	0	0	0	0	0	0	0	0	0	0	0	0	0	0	0	0
	R_{yx}					1	2	30	35	38	30	18	9	3	3	4	5	5	4	4	1	1	0	0
8	R_{xy}			0	0	1	0	4	3	7	5	1	2	1	1	13	24	27	33	30	3	1		
	R_{si}			0	0	0	0	0	6	19	28	33	25	31	42	39	26	13	1	0	0	0		
	R_{su}			0	0	0	0	0	0	0	0	0	0	0	0	0	0	0	0	0	0	0		
	R_{yx}			1	1	4	7	35	42	33	18	2	4	1	0	0	2	4	2	1	0	0		
9	R_{xy}					0	0	1	4	7	1	0	0	0	0	2	15	36	44	41	5	2		
	R_{si}					0	0	1	3	14	31	34	31	24	24	33	30	8	2	0	0	0		
	R_{su}					0	0	0	5	4	3	2	0	0	1	0	4	3	4	0	0	0		
	R_{yx}					1	4	36	35	30	19	4	1	0	0	2	8	12	3	2	0	0		
10	R_{xy}					0	0	1	1	4	5	2	3	2	4	15	38	33	29	25	1			
	R_{si}					0	0	1	0	2	17	29	28	23	38	36	13	3	1	0	0			
	R_{su}					0	1	0	1	3	10	14	8	2	6	12	9	5	1	1	0			
	R_{yx}					1	2	30	35	43	30	5	1	0	0	1	1	0	0	0	0			

Note. The values were reported by García-Pérez and Alcalá-Quintana (2018) in their supplementary material. We used these values to calculate the response proportions in Table S8. x: right; y: left.

Table S19

Response frequencies from the subjects 11–19 in the study by García-Pérez and Alcalá-Quintana (2018)

Subject	Response	Stimulus-onset difference d [ms]																						
		−200	−183	−167	−150	−133	−117	−100	−83	−67	−50	−33	−17	0	17	33	50	67	83	100	117	133	150	167
11	R_{xy}						0	0	1	3	19	6	7	9	10	18	32	57	53	41	5	1		
	R_{si}						0	0	0	0	0	0	0	0	0	0	0	0	0	0	0	0		
	R_{su}						0	0	0	0	2	6	9	2	7	3	1	1	0	0	0	0		
	R_{yx}						1	28	44	46	30	31	17	16	9	21	19	16	4	1	0	0		
12	R_{xy}					0	0	2	11	10	7	3	1	0	2	4	20	29	38	44	18	11	3	1
	R_{si}					0	0	1	1	9	26	24	13	14	17	22	13	4	3	0	0	1	0	0
	R_{su}					0	0	0	0	4	4	3	2	0	2	4	3	2	0	0	0	0	0	0
	R_{yx}					1	13	43	42	39	21	2	0	0	0	1	4	13	8	10	2	1	0	0
13	R_{xy}						0	0	2	7	7	8	3	5	2	7	8	31	38	40	13	4	1	1
	R_{si}						0	1	1	6	21	25	20	16	19	22	34	17	9	3	0	0	0	0
	R_{su}						0	0	0	2	0	0	0	0	0	1	0	0	0	0	0	0	0	0
	R_{yx}						1	30	39	42	32	11	5	4	5	8	7	7	6	4	0	1	0	0
14	R_{xy}			0	0	1	1	12	4	7	1	1	0	0	0	0	5	23	32	26	6	3		
	R_{si}			0	0	1	5	11	24	28	26	24	11	8	15	31	49	39	17	7	2	1		
	R_{su}			0	0	0	0	4	2	4	4	1	0	0	0	1	3	5	4	2	1	0		
	R_{yx}			1	3	13	15	35	30	12	4	0	1	0	0	2	0	2	2	4	0	0		
15	R_{xy}					0	0	4	5	9	8	2	0	0	0	4	35	40	30	26	2			
	R_{si}					0	0	0	0	5	24	35	24	17	31	42	23	3	0	0	0			
	R_{su}					0	0	2	0	5	11	3	1	1	1	7	8	5	0	1	0			
	R_{yx}					3	10	37	42	39	21	4	0	0	0	1	3	1	1	0	0			
16	R_{xy}							0	0	0	0	5	14	19	31	32	45	38	28	24				
	R_{si}							0	0	0	0	7	19	20	9	9	1	0	0	0				
	R_{su}							0	0	0	2	4	8	6	8	4	1	0	0	0				
	R_{yx}							24	24	29	41	39	30	24	12	13	5	1	0	0				
17	R_{xy}					0	0	2	2	3	9	5	2	3	3	6	19	40	30	28	4	1		
	R_{si}					0	0	1	2	4	25	23	23	15	24	26	21	9	1	0	0	0		
	R_{su}					0	0	2	4	11	11	9	10	9	7	7	13	2	3	2	1	0		
	R_{yx}					2	5	30	42	40	13	11	1	0	2	1	3	0	3	1	0	0		
18	R_{xy}							0	0	4	8	9	2	3	2	10	35	41	35	30	1			
	R_{si}							1	0	9	16	29	32	27	26	34	22	9	0	0	0			
	R_{su}							0	0	0	0	0	0	0	0	0	0	0	0	0	0			
	R_{yx}							27	42	44	36	12	5	0	4	7	7	6	1	0	0			
19	R_{xy}						0	0	4	6	0	1	1	0	0	1	4	15	35	41	19	6	3	2
	R_{si}						0	3	7	21	37	28	34	28	22	23	29	28	12	3	0	0	0	0
	R_{su}						0	0	4	2	3	1	2	0	0	1	0	5	2	1	0	0	0	0
	R_{yx}						5	37	33	29	9	1	0	0	0	1	2	3	14	5	1	2	0	0

Note. The values were reported by García-Pérez and Alcalá-Quintana (2018) in their supplementary material. We used these values to calculate the response proportions in Table S9. x: right; y: left.

Table S20

Response frequencies in the study by Lahkar et al. (2023)

Response	Stimulus-onset difference d [ms]																										
	−65	−60	−55	−50	−45	−40	−35	−30	−25	−20	−15	−10	−5	0	5	10	15	20	25	30	35	40	45	50	55	60	65
R_{xy}	1	4	3	4	4	8	8	4	4	8	2	1	2	9	9	16	36	72	114	153	161	189	216	222	225	232	236
R_{si}	0	3	4	19	22	38	50	94	137	163	205	220	229	457	229	221	198	160	120	82	72	46	21	16	12	7	1
R_{yx}	239	233	233	217	214	194	182	142	99	69	33	19	9	14	2	3	6	8	6	5	7	5	3	2	3	1	3

Note. We calculated the values based on the data provided to us by Lahkar et al. (2023). We used these values to calculate the response proportions in Table S10. x: right; y: left.

Graphical representation of data and model fits

Models assuming normally distributed arrival-latency differences

Representation by three psychometric functions

Figures S1–13 depict the observed and fitted psychometric functions $P(R_{xy} \mid d)$, $P(R_{si} \mid d)$, and $P(R_{yx} \mid d)$ for all studies except Ulrich (1987), which are shown in Figure 3.

Figure S1

Observed (points) and fitted (lines) psychometric functions $P(R_{xy} \mid d)$, $P(R_{si} \mid d)$, and $P(R_{yx} \mid d)$ for the study by Benussi (1913)

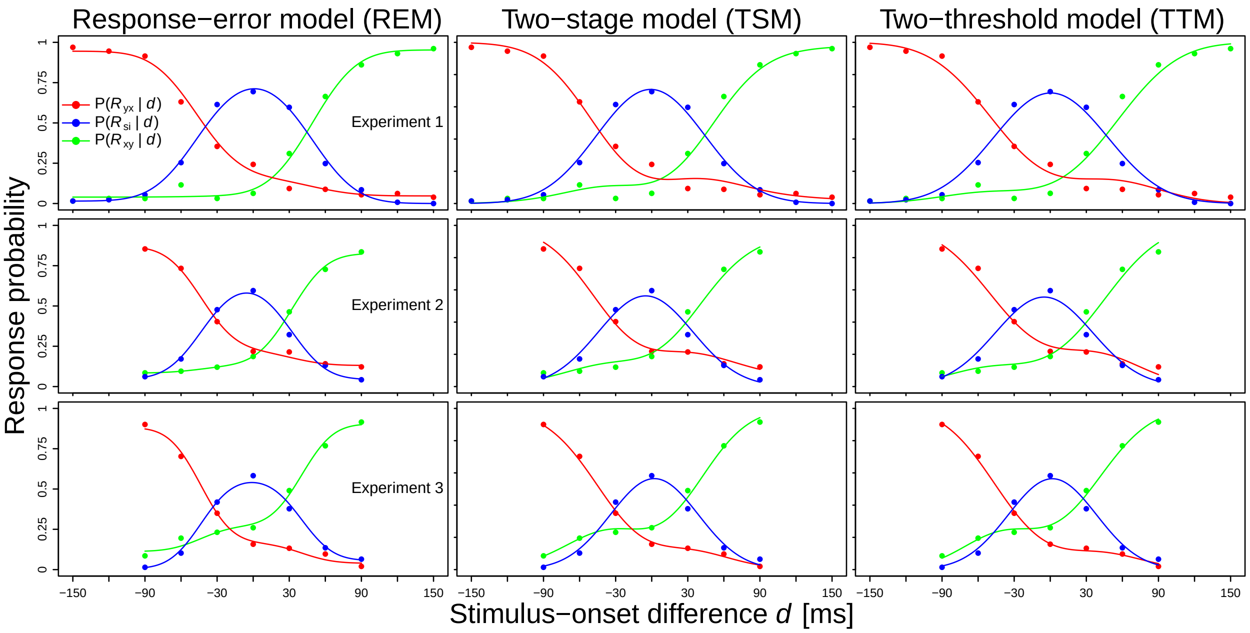

Note. x: left; y: right.

Figure S2

Observed (points) and fitted (lines) psychometric functions $P(R_{xy} \mid d)$, $P(R_{si} \mid d)$, and $P(R_{yx} \mid d)$ for the study by Allan (1975a)

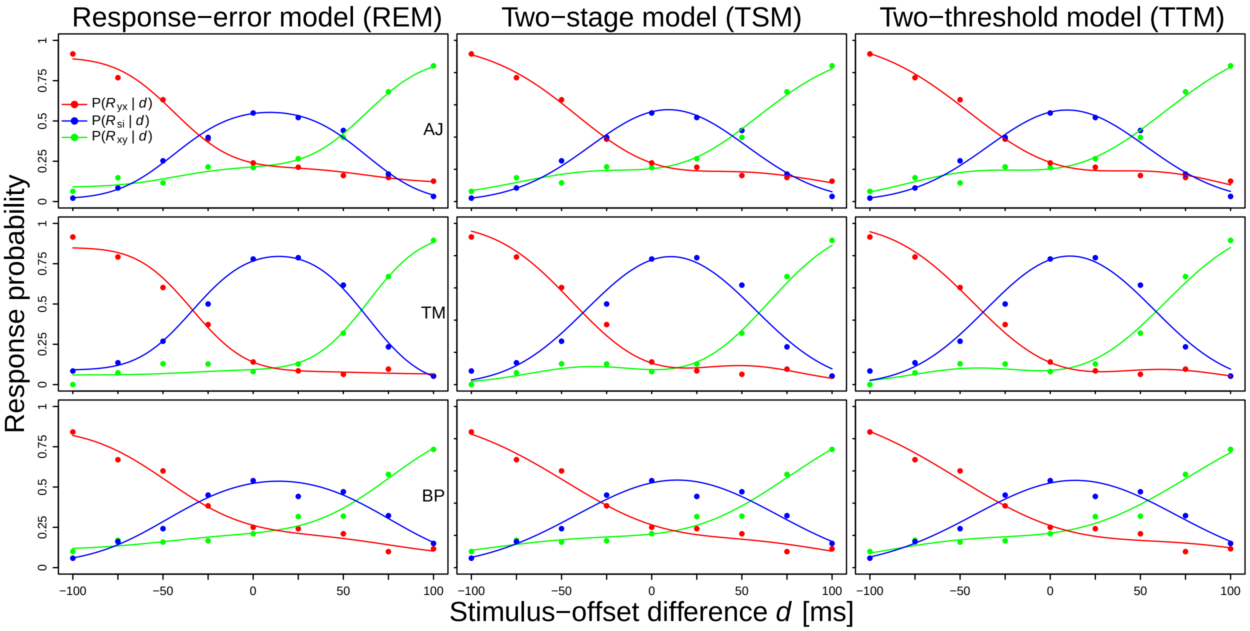

Note. x: visual; y: auditory.

Figure S3

Observed (points) and fitted (lines) psychometric functions $P(R_{xy} \mid d)$, $P(R_{si} \mid d)$, and $P(R_{yx} \mid d)$ for the study by Allan (1975b)

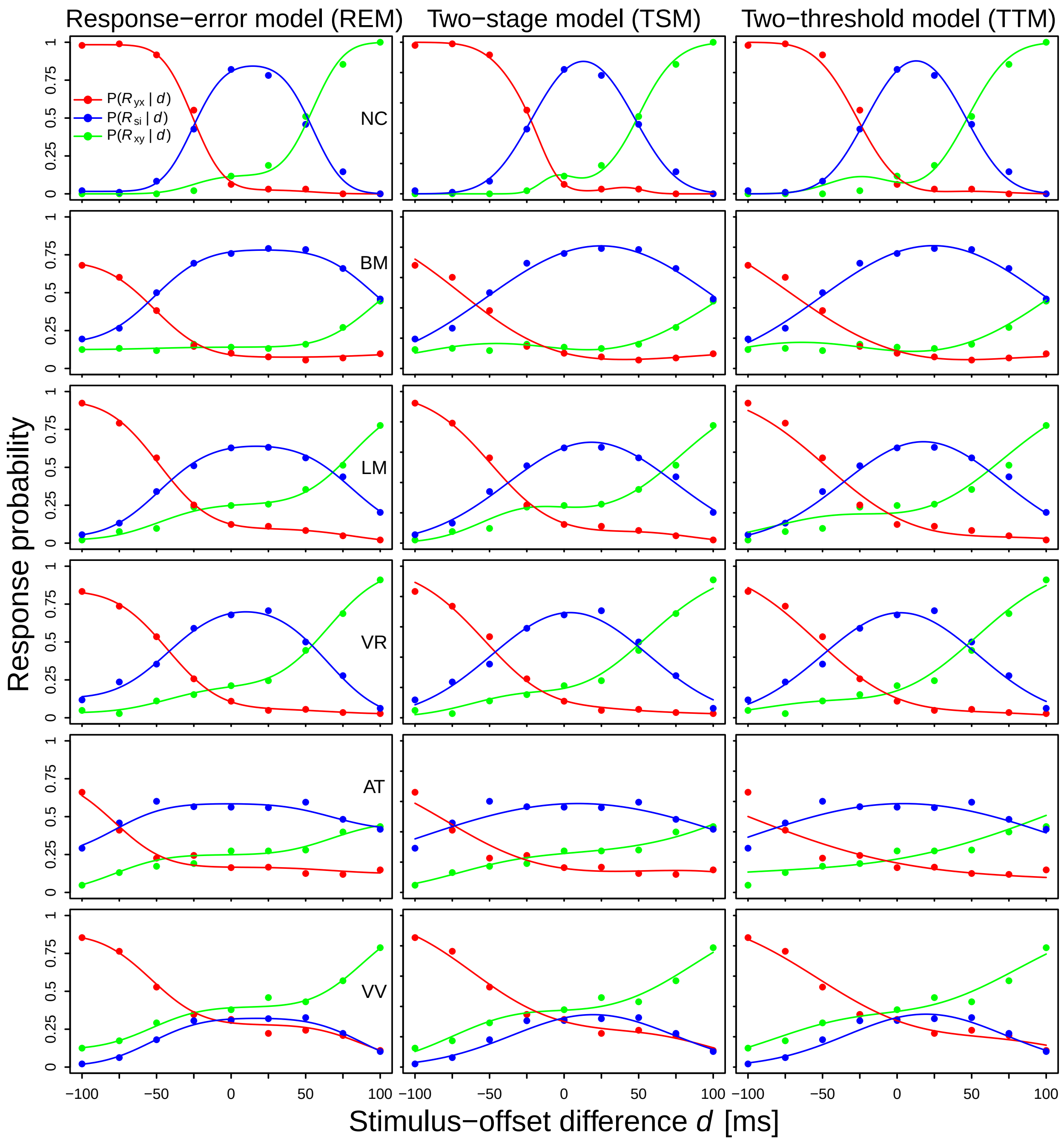

Note. x: visual; y: auditory.

Figure S4

Observed (points) and fitted (lines) psychometric functions $P(R_{xy} \mid d)$, $P(R_{si} \mid d)$, and $P(R_{yx} \mid d)$ for the subjects PJ and PT in the study by Jaśkowski (1991a, Experiment 3)

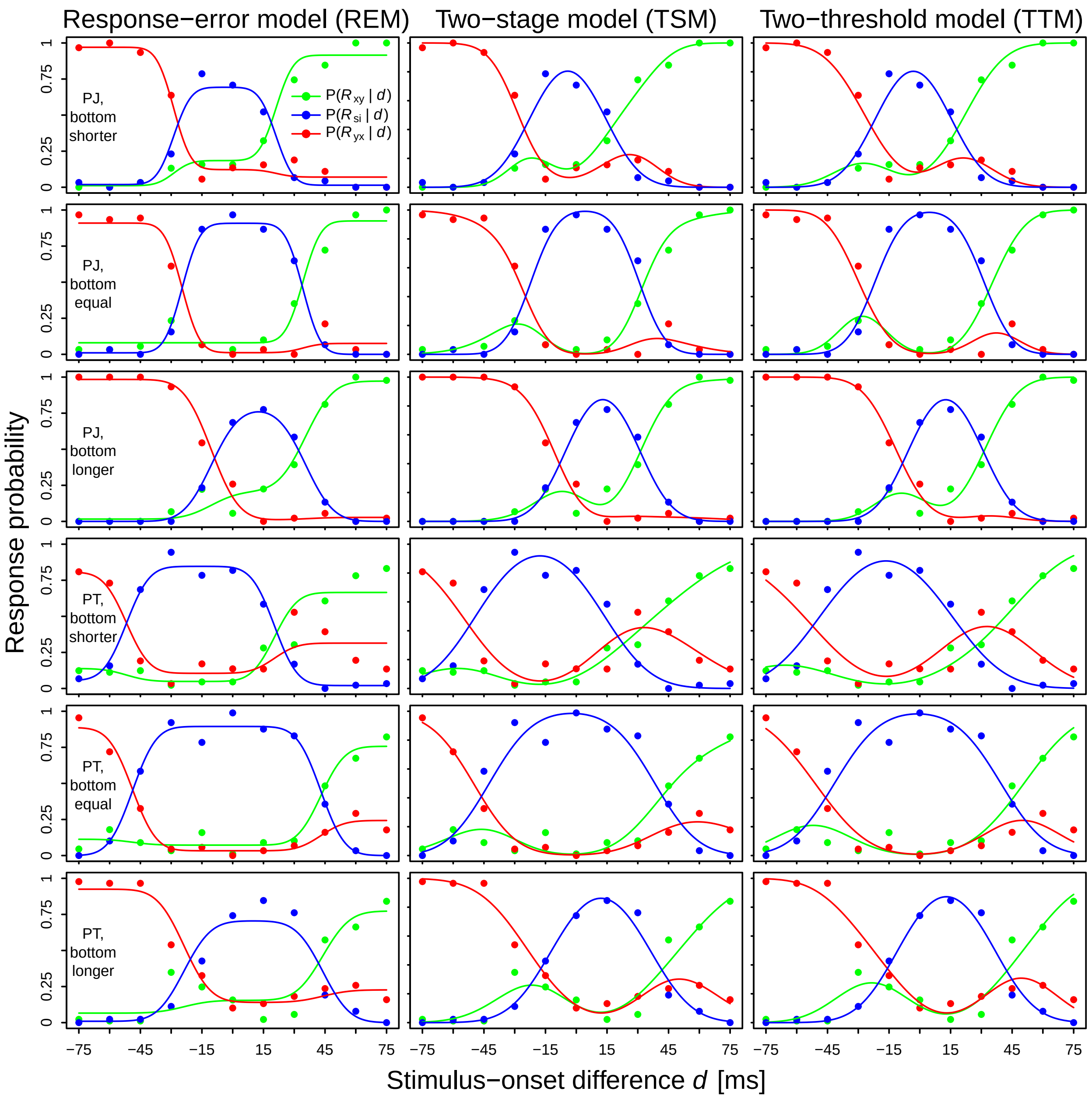

Note. x: bottom; y: top.

Figure S5

Observed (points) and fitted (lines) psychometric functions $P(R_{xy}\,|\,d)$, $P(R_{si}\,|\,d)$, and $P(R_{yx}\,|\,d)$ for the subject WW in the study by Jaśkowski (1991a, Experiment 3)

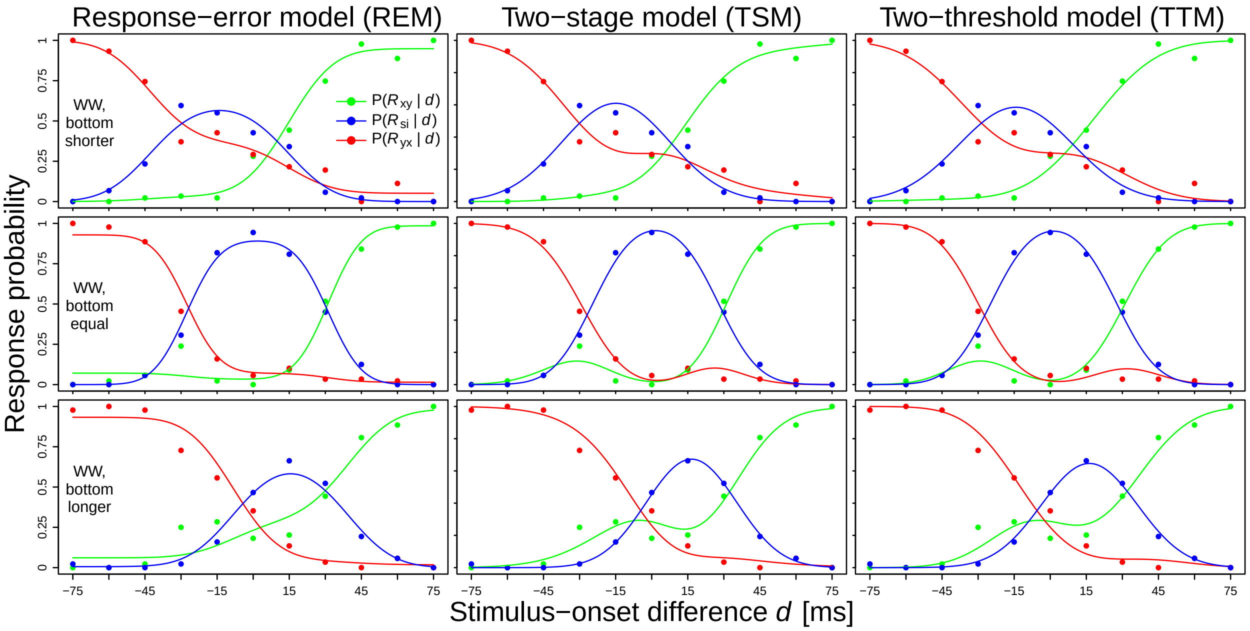

Note. x: bottom; y: top.

Figure S6

Observed (points) and fitted (lines) psychometric functions $P(R_{xy} \mid d)$, $P(R_{si} \mid d)$, and $P(R_{yx} \mid d)$ for subjects 1–4 and 6 with simple stimuli in the study by van Eijk et al. (2008)

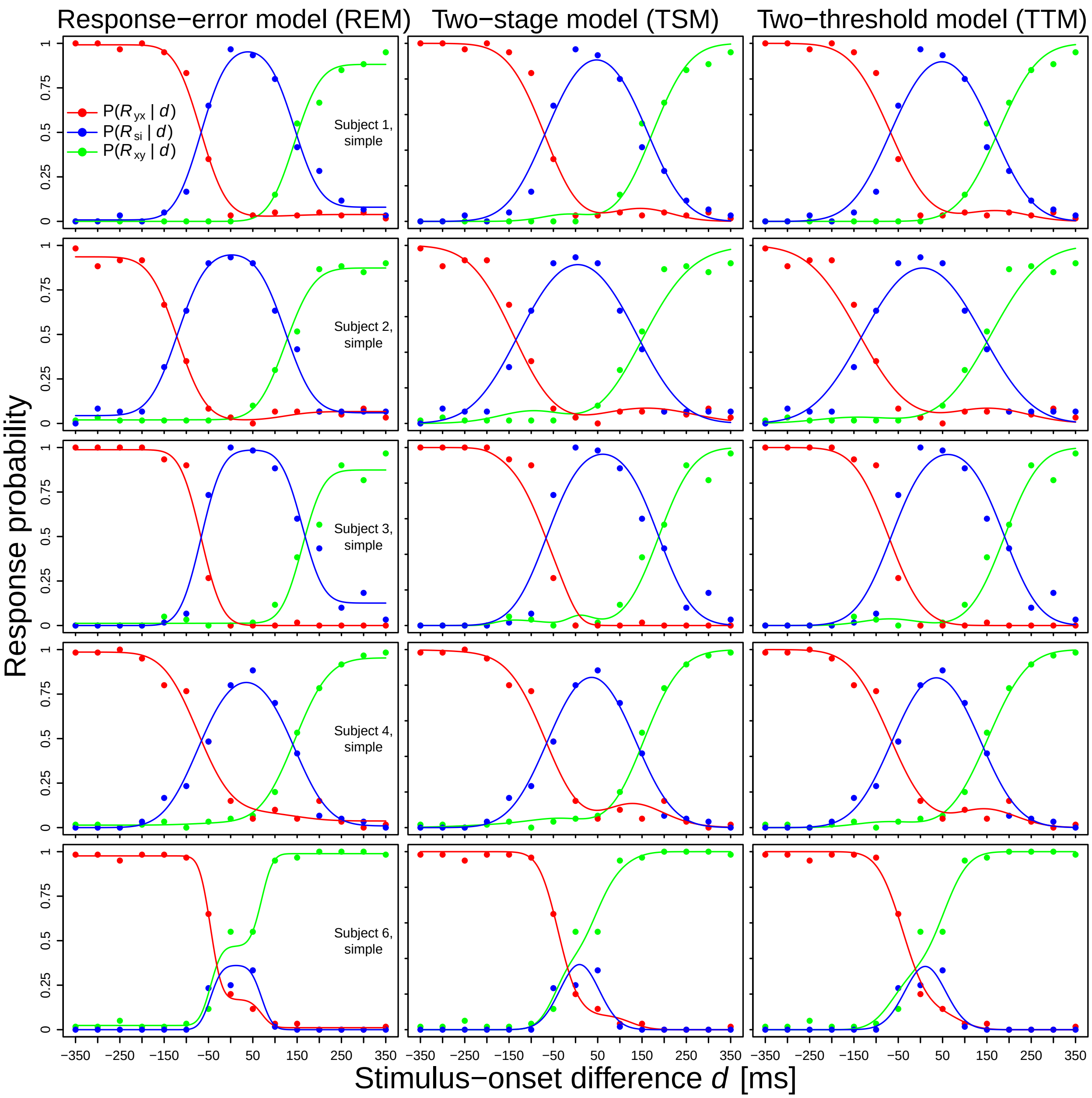

Note. x: visual; y: auditory.

Figure S7

Observed (points) and fitted (lines) psychometric functions $P(R_{xy} \mid d)$, $P(R_{si} \mid d)$, and $P(R_{yx} \mid d)$ for subjects 7–12 with simple stimuli in the study by van Eijk et al. (2008)

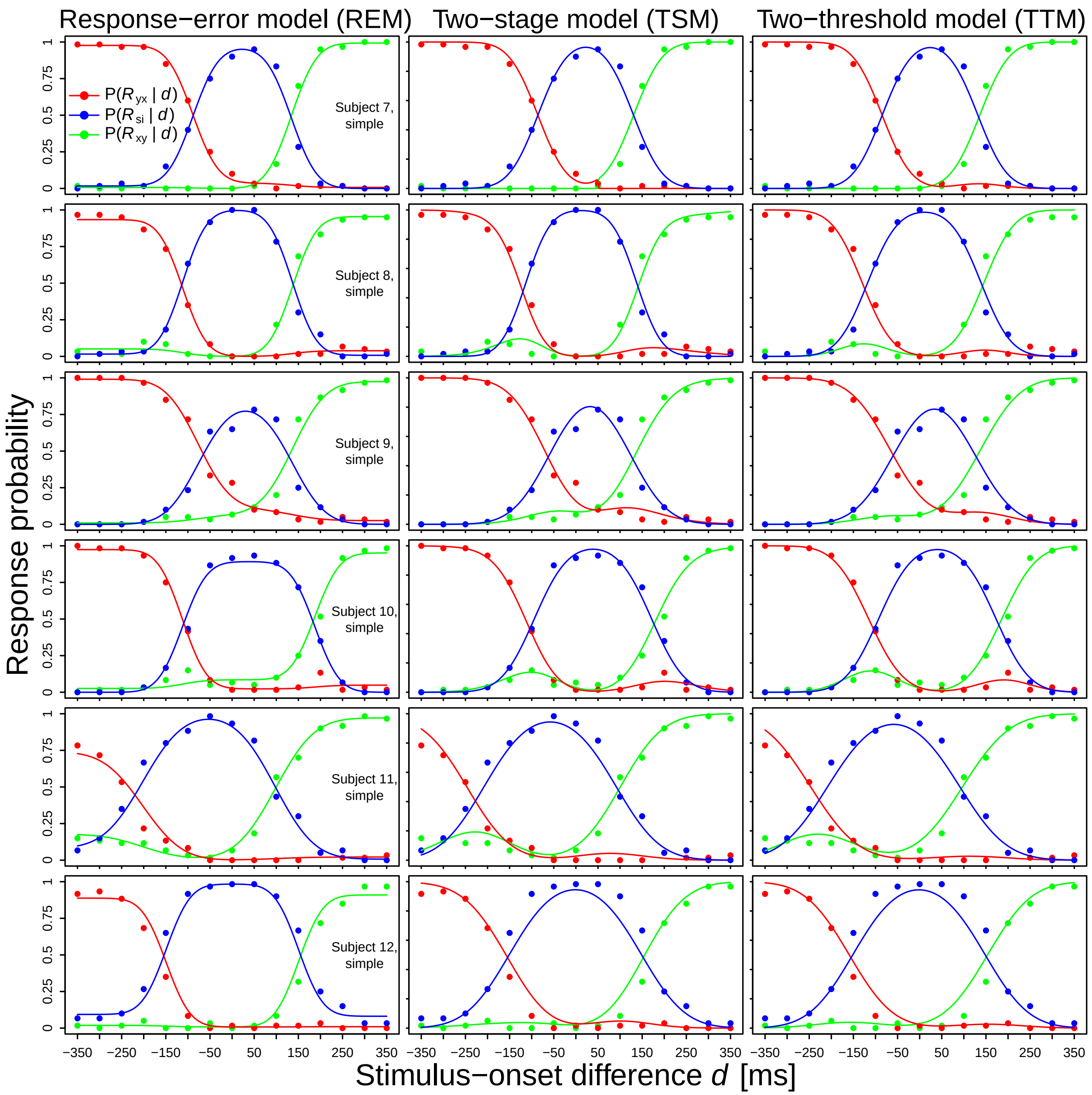

Note. x: visual; y: auditory.

Figure S8

Observed (points) and fitted (lines) psychometric functions $P(R_{xy} \mid d)$, $P(R_{si} \mid d)$, and $P(R_{yx} \mid d)$ for subjects 1–6 with complex stimuli in the study by van Eijk et al. (2008)

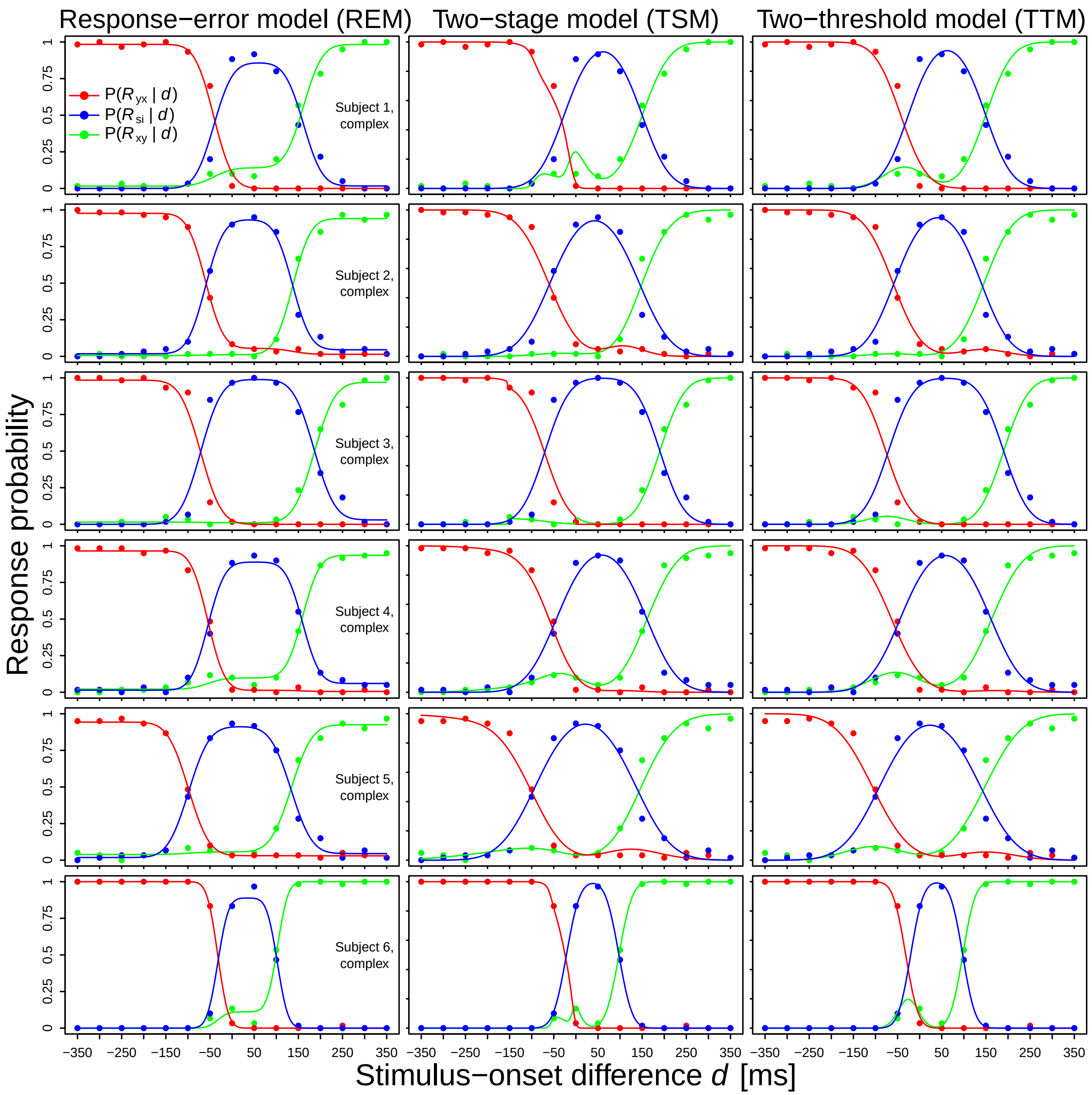

Note. x: visual; y: auditory.

Figure S9

Observed (points) and fitted (lines) psychometric functions $P(R_{xy} \mid d)$, $P(R_{si} \mid d)$, and $P(R_{yx} \mid d)$ for subjects 7–12 with complex stimuli in the study by van Eijk et al. (2008)

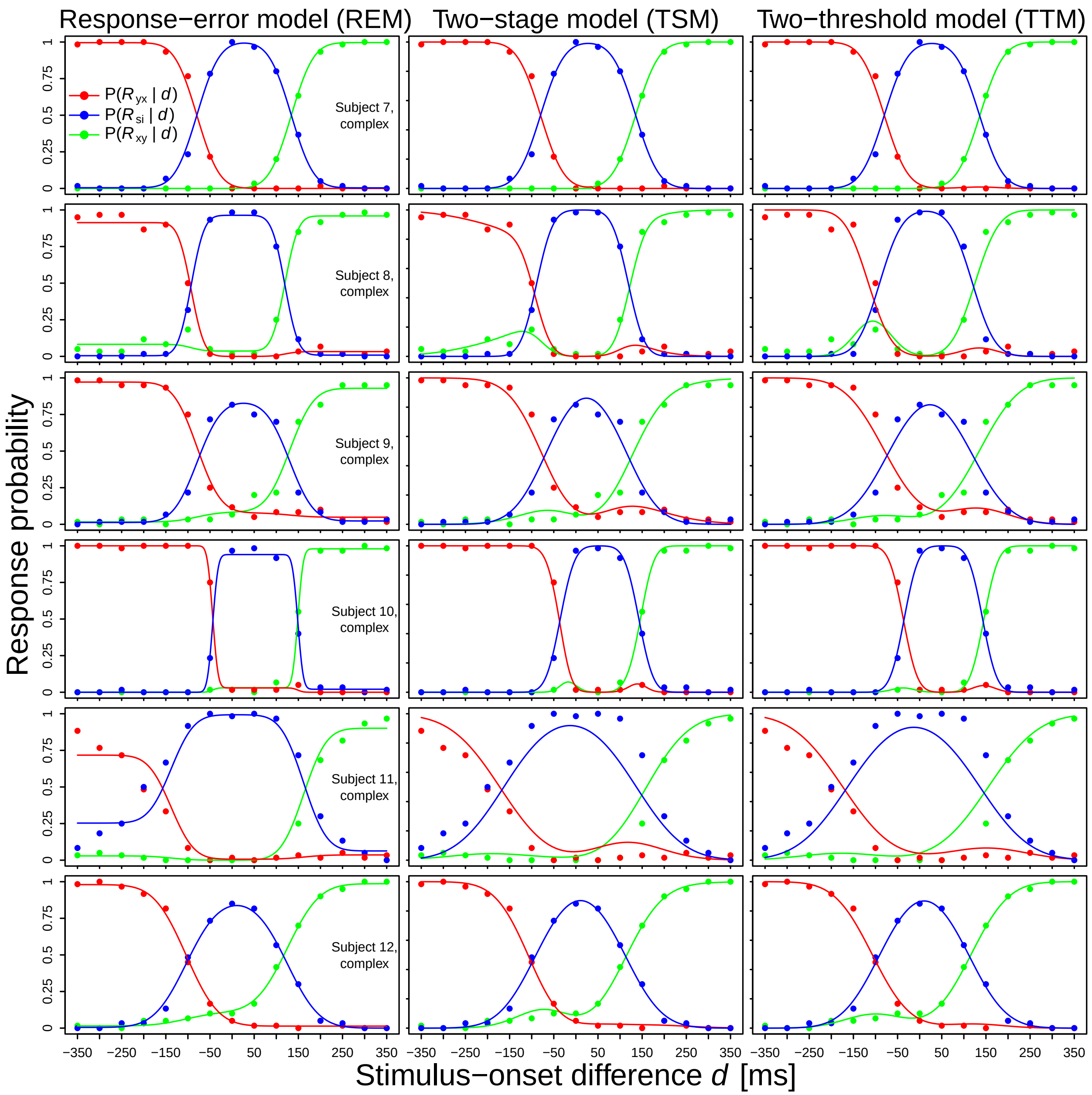

Note. x: visual; y: auditory.

Figure S10

Observed (points) and fitted (lines) psychometric functions $P(R_{xy} \mid d)$, $P(R_{si} \mid d)$, and $P(R_{yx} \mid d)$ for subjects 1–6 in the study by García-Pérez and Alcalá-Quintana (2018)

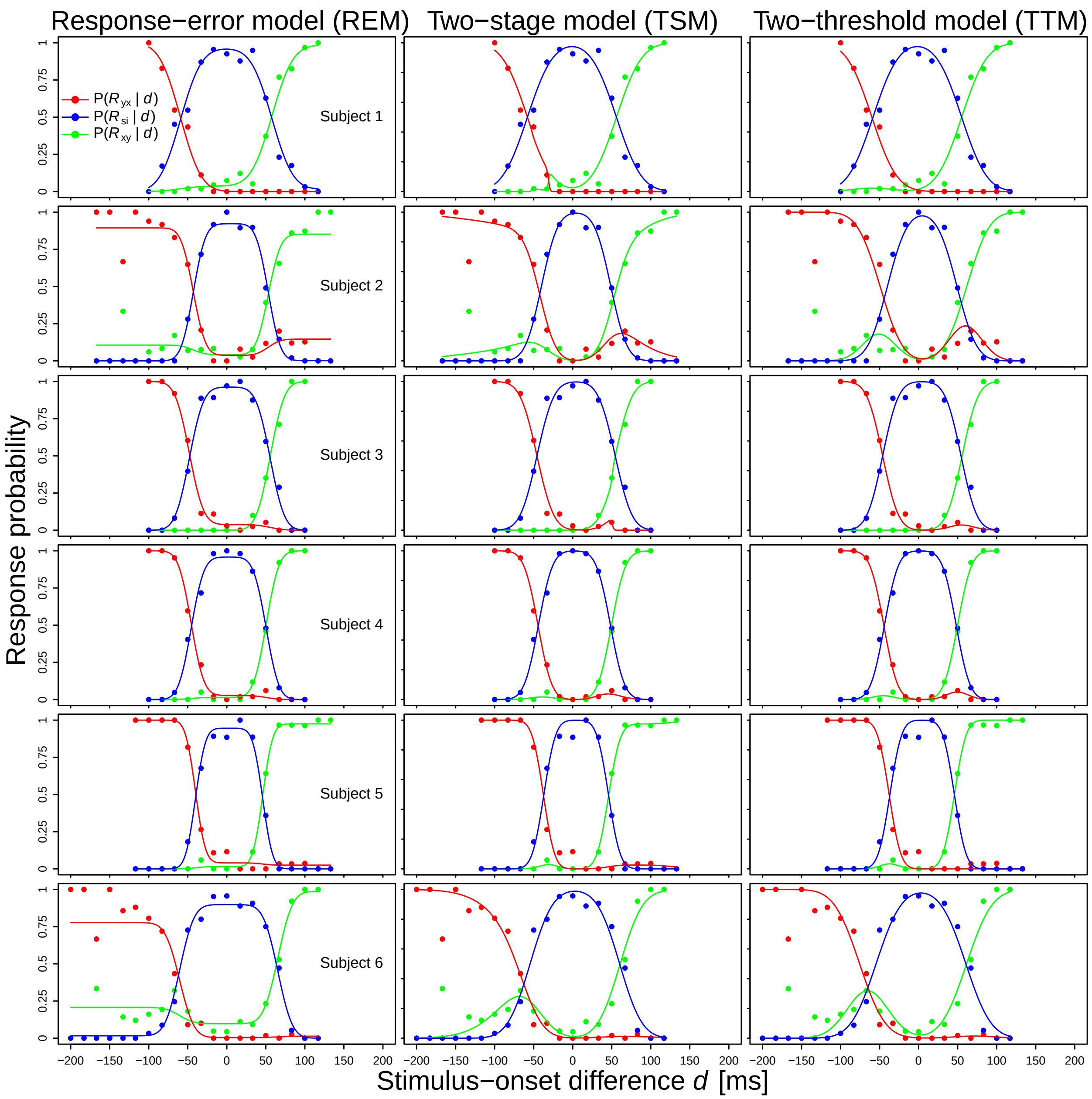

Note. x: right; y: left.

Figure S11

Observed (points) and fitted (lines) psychometric functions $P(R_{xy} \,|\, d)$, $P(R_{si} \,|\, d)$, and $P(R_{yx} \,|\, d)$ for subjects 7–12 in the study by García-Pérez and Alcalá-Quintana (2018)

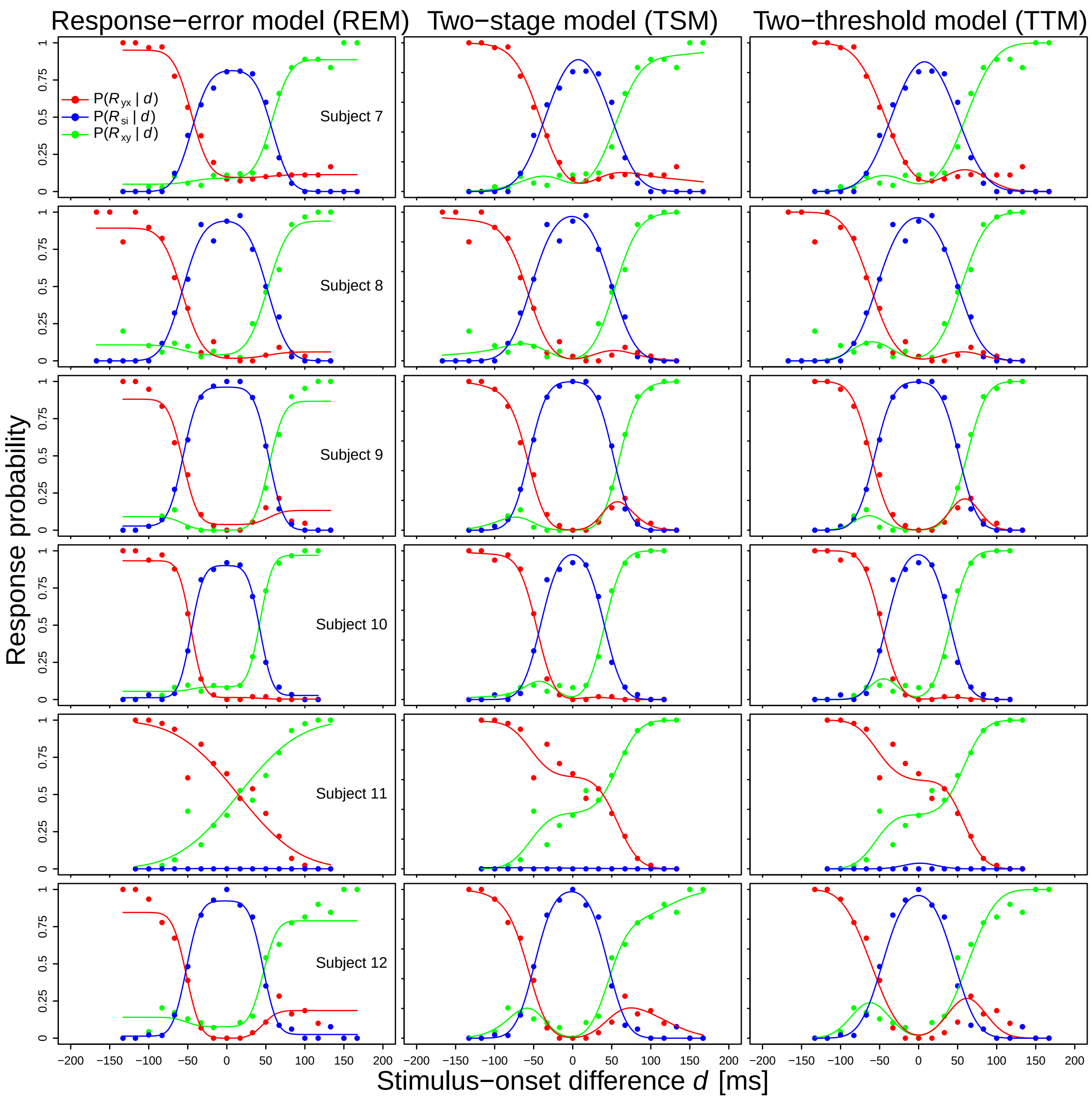

Note. x: right; y: left.

Figure S12

Observed (points) and fitted (lines) psychometric functions $P(R_{xy} \,|\, d)$, $P(R_{si} \,|\, d)$, and $P(R_{yx} \,|\, d)$ for subjects 13–19 in the study by García-Pérez and Alcalá-Quintana (2018)

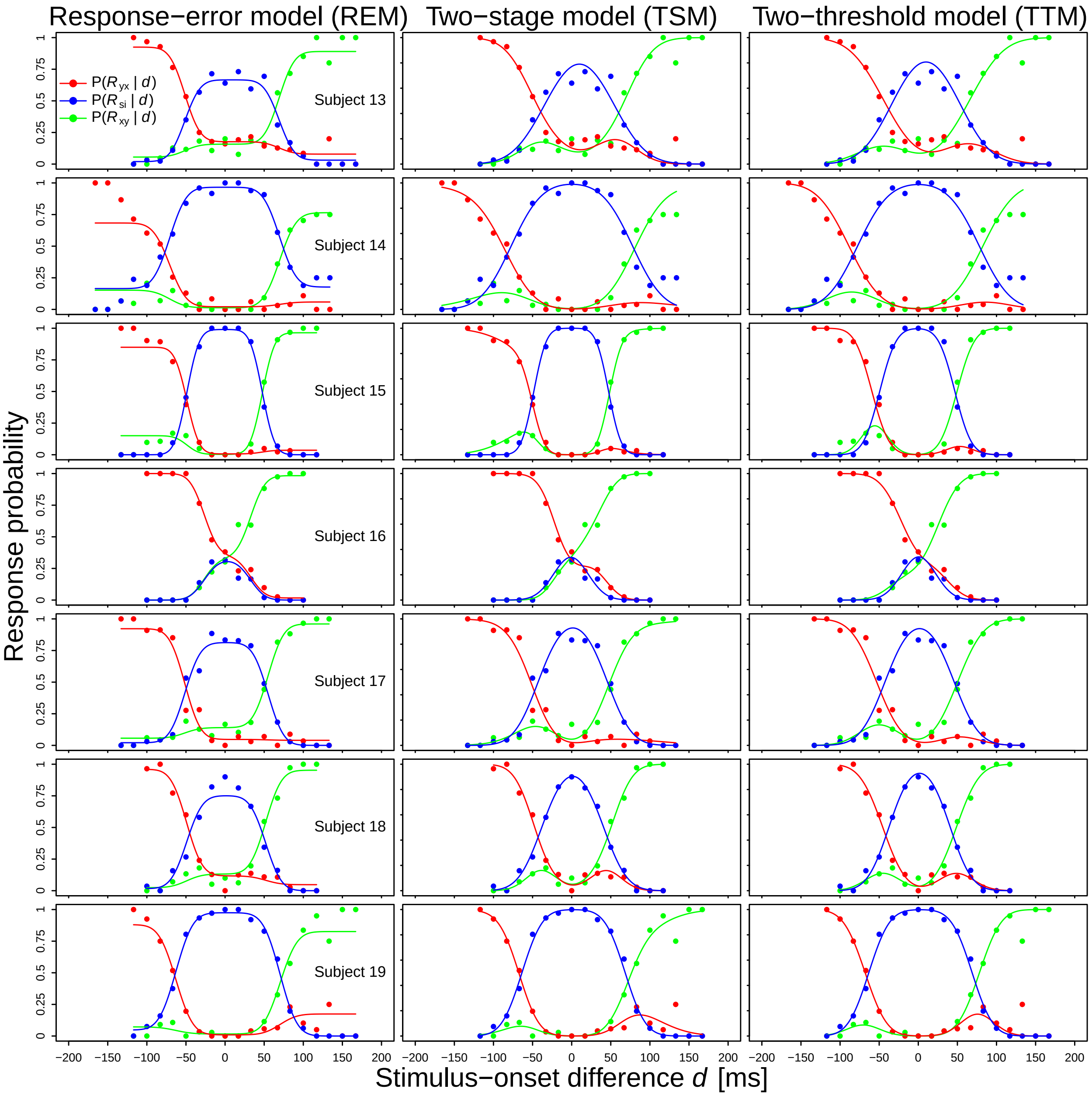

Note. x: right; y: left.

Figure S13

Observed (points) and fitted (lines) psychometric functions $P(R_{xy} \mid d)$, $P(R_{si} \mid d)$, and $P(R_{yx} \mid d)$ for the study by Lahkar et al. (2023)

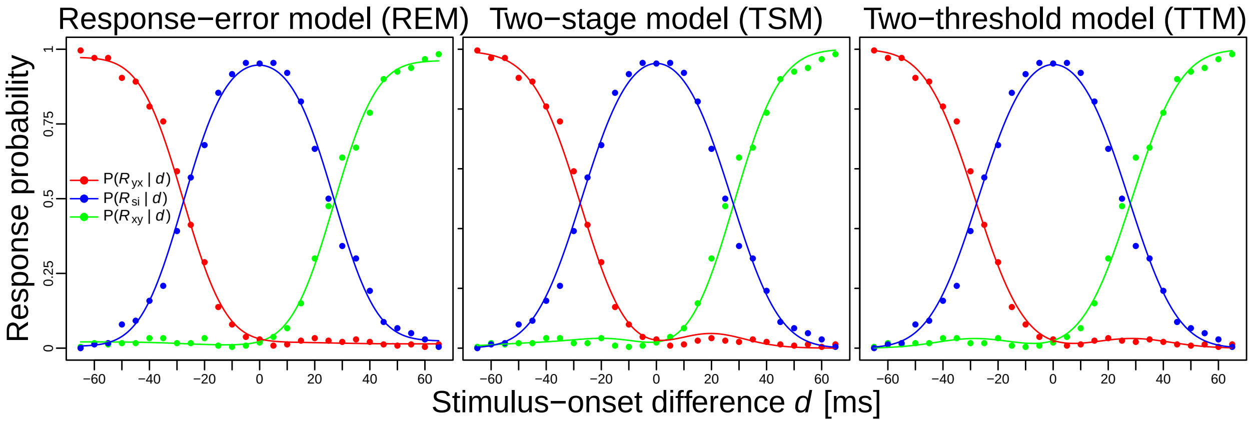

Note. x: right; y: left.

Representation by two psychometric functions

Figures S14–26 depict the observed and fitted psychometric functions $P(R_{xy} \mid d)$ and $1 - P(R_{yx} \mid d)$ for all studies except Ulrich (1987), which are shown in Figure 4.

Figure S14

Observed (points) and fitted (lines) psychometric functions $P(R_{xy} \mid d)$ and $1 - P(R_{yx} \mid d)$ for the study by Benussi (1913)

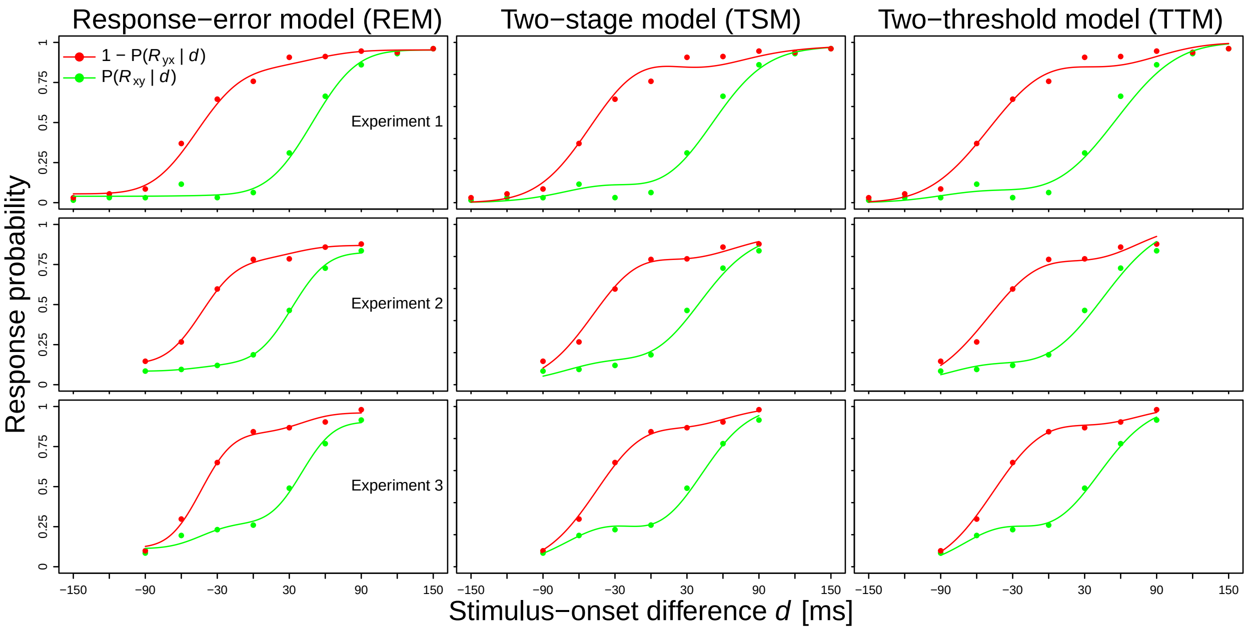

Note. x: left; y: right.

Figure S15

Observed (points) and fitted (lines) psychometric functions $P(R_{xy} \mid d)$ and $1 - P(R_{yx} \mid d)$ for the study by Allan (1975a)

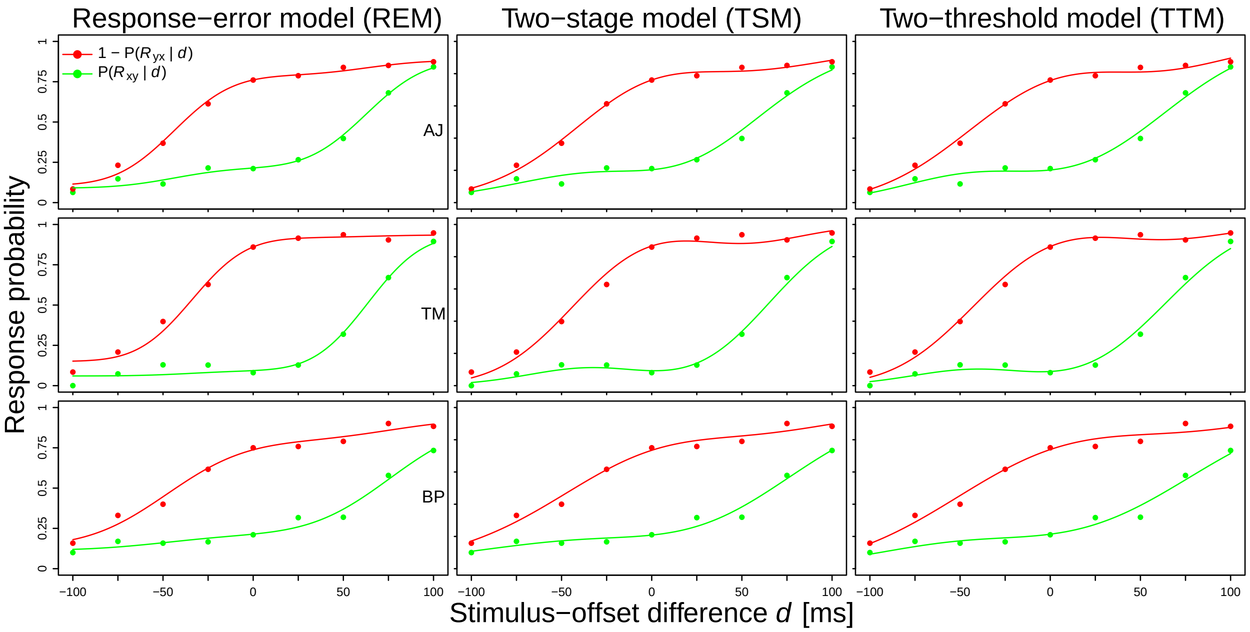

Note. x: visual; y: auditory.

Figure S16

Observed (points) and fitted (lines) psychometric functions $P(R_{xy} \mid d)$ *and* $1 - P(R_{yx} \mid d)$ *for the study by Allan (1975b)*

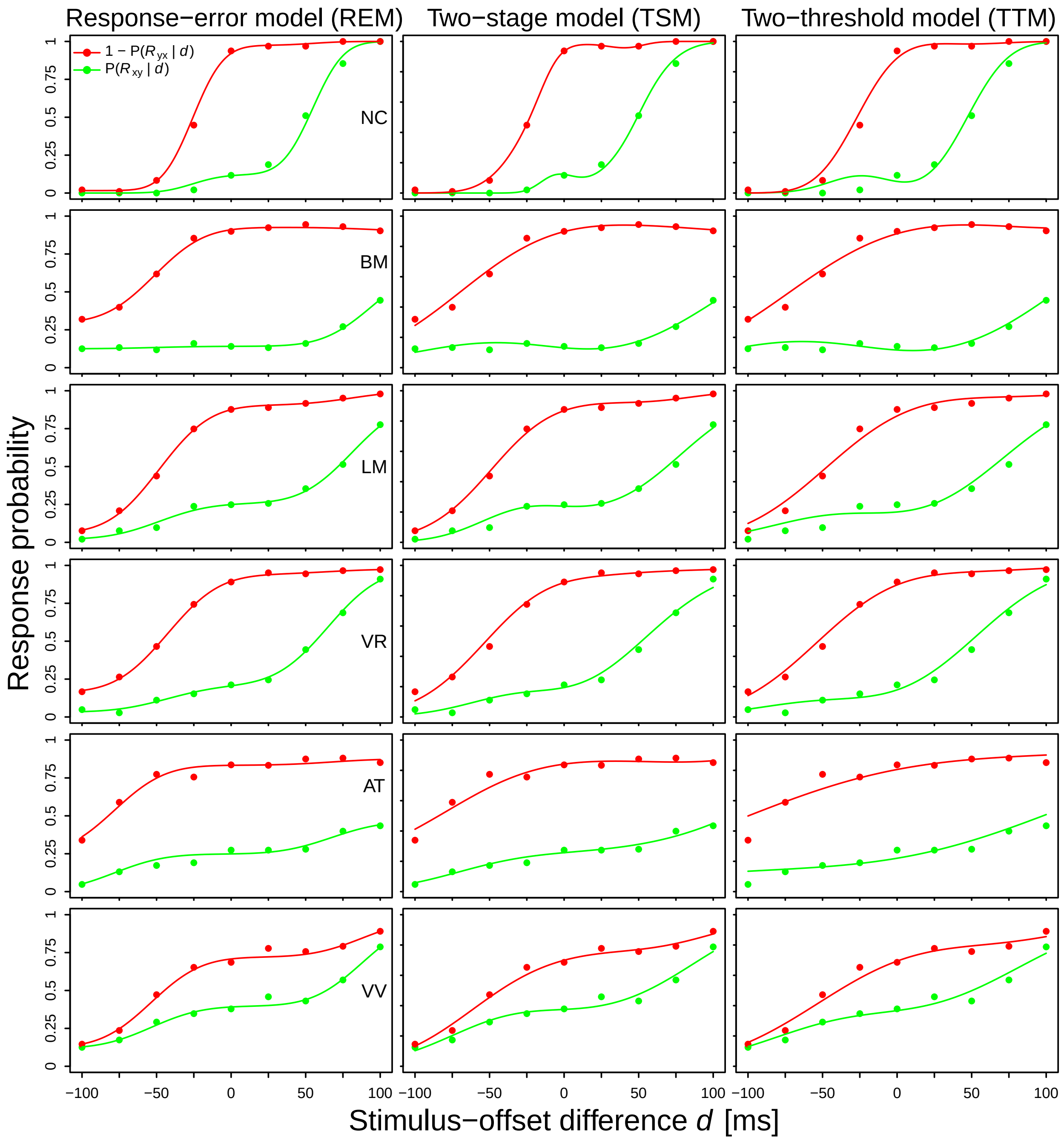

Note. x: visual; y: auditory.

Figure S17

Observed (points) and fitted (lines) psychometric functions $P(R_{xy} \mid d)$ and $1 - P(R_{yx} \mid d)$ for the subjects PJ and PT in the study by Jaśkowski (1991a, Experiment 3)

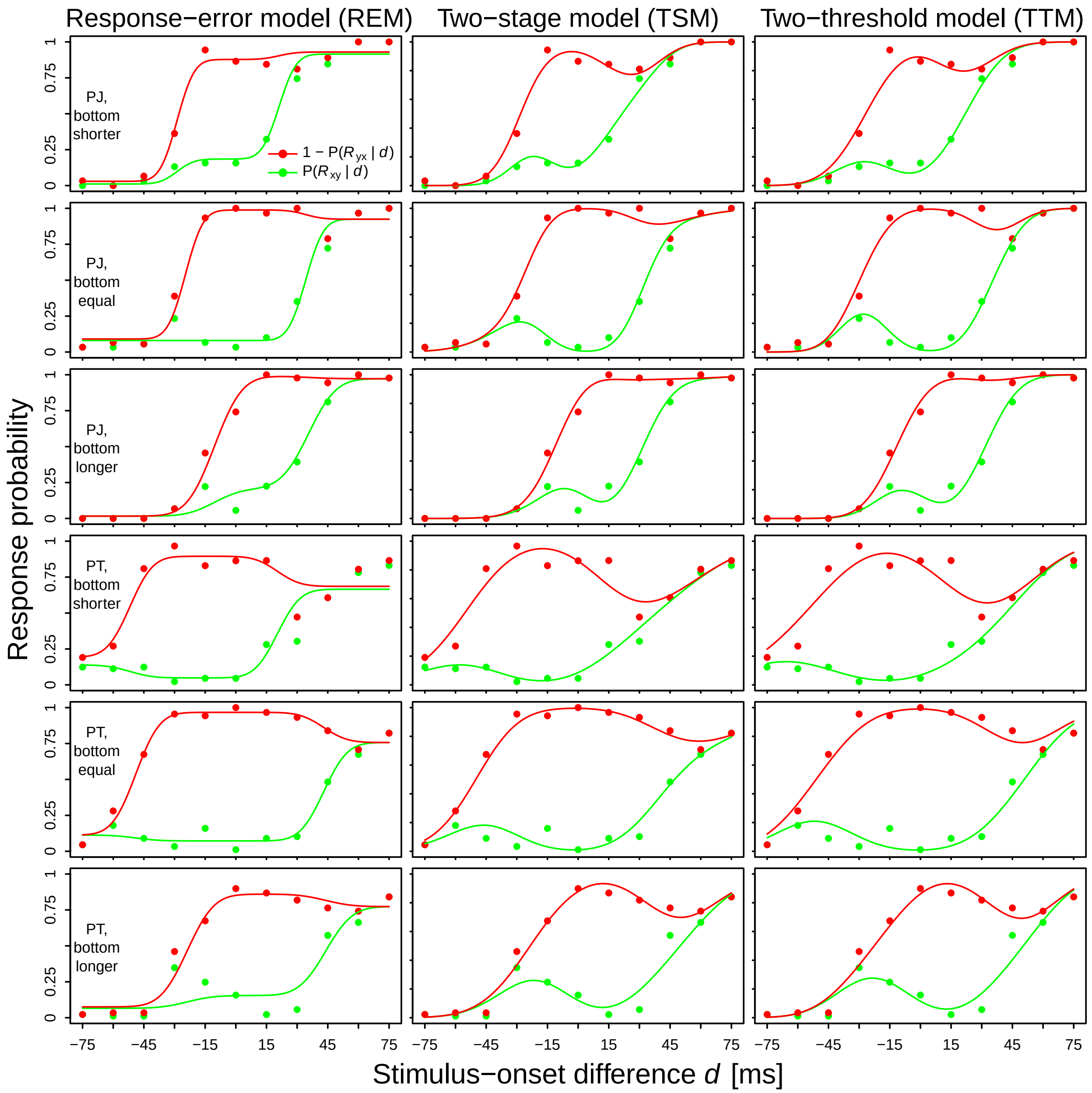

Note. x: bottom; y: top.

Figure S18

Observed (points) and fitted (lines) psychometric functions $P(R_{xy} \mid d)$ and $1 - P(R_{yx} \mid d)$ for the subject WW in the study by Jaśkowski (1991a, Experiment 3)

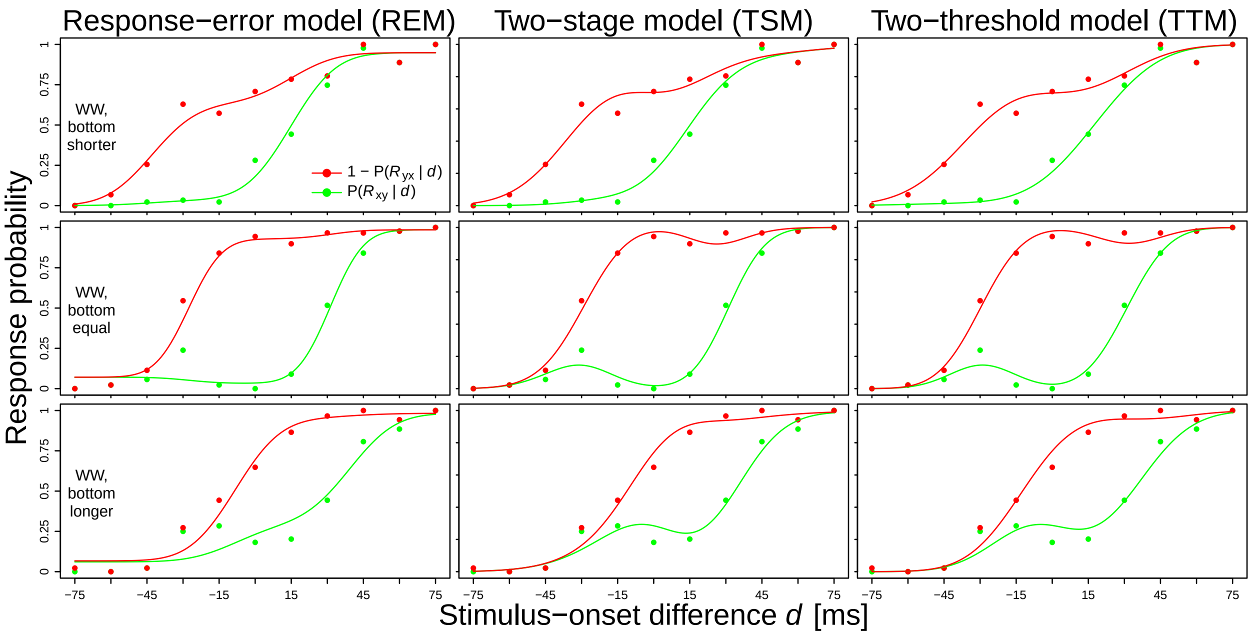

Note. x: bottom; y: top.

Figure S19

Observed (points) and fitted (lines) psychometric functions $P(R_{xy} \mid d)$ and $1 - P(R_{yx} \mid d)$ for subjects 1–4 and 6 with simple stimuli in the study by van Eijk et al. (2008)

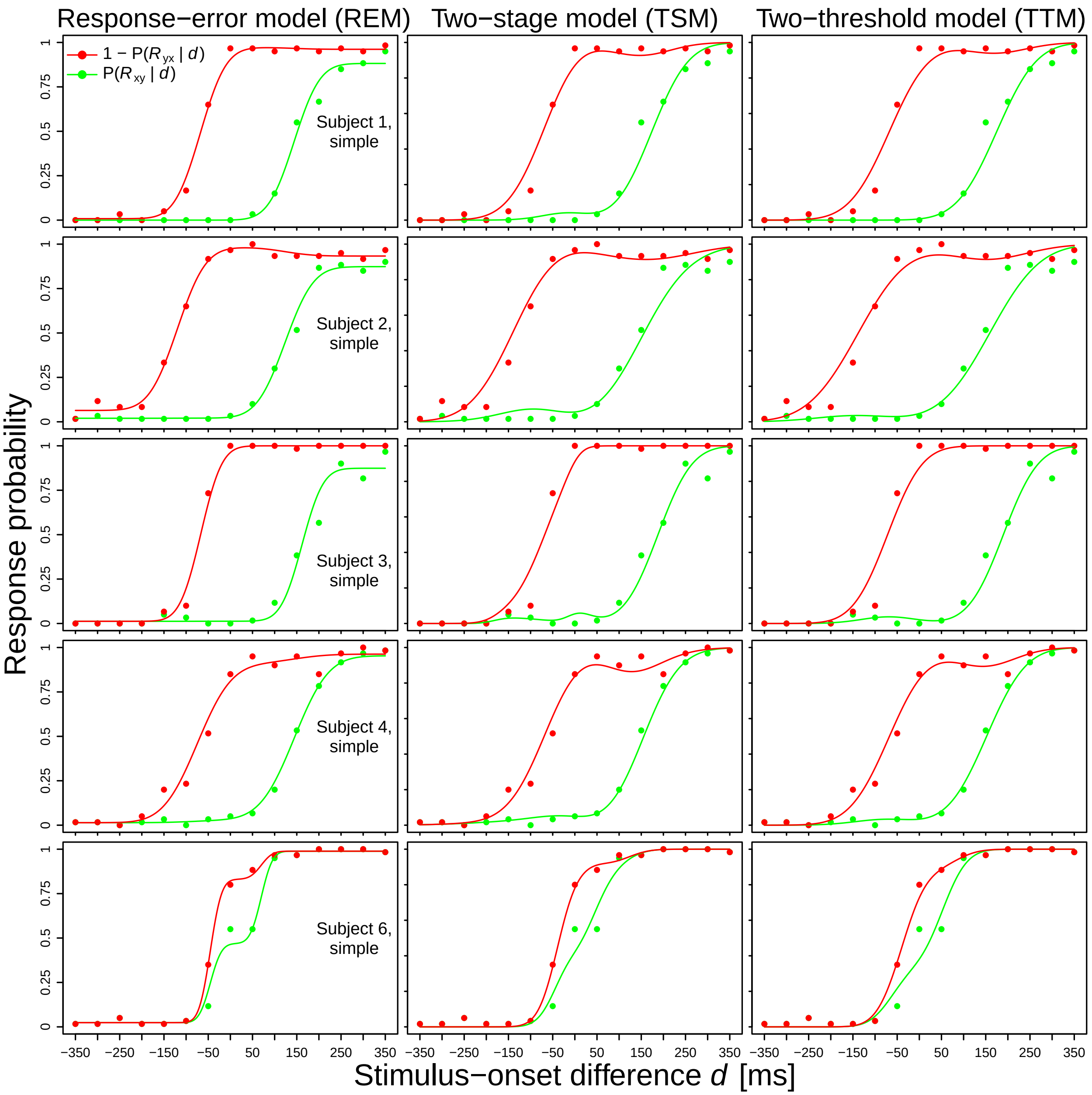

Note. x: visual; y: auditory.

Figure S20

Observed (points) and fitted (lines) psychometric functions $P(R_{xy}\,|\,d)$ and $1 - P(R_{yx}\,|\,d)$ for subjects 7–12 with simple stimuli in the study by van Eijk et al. (2008)

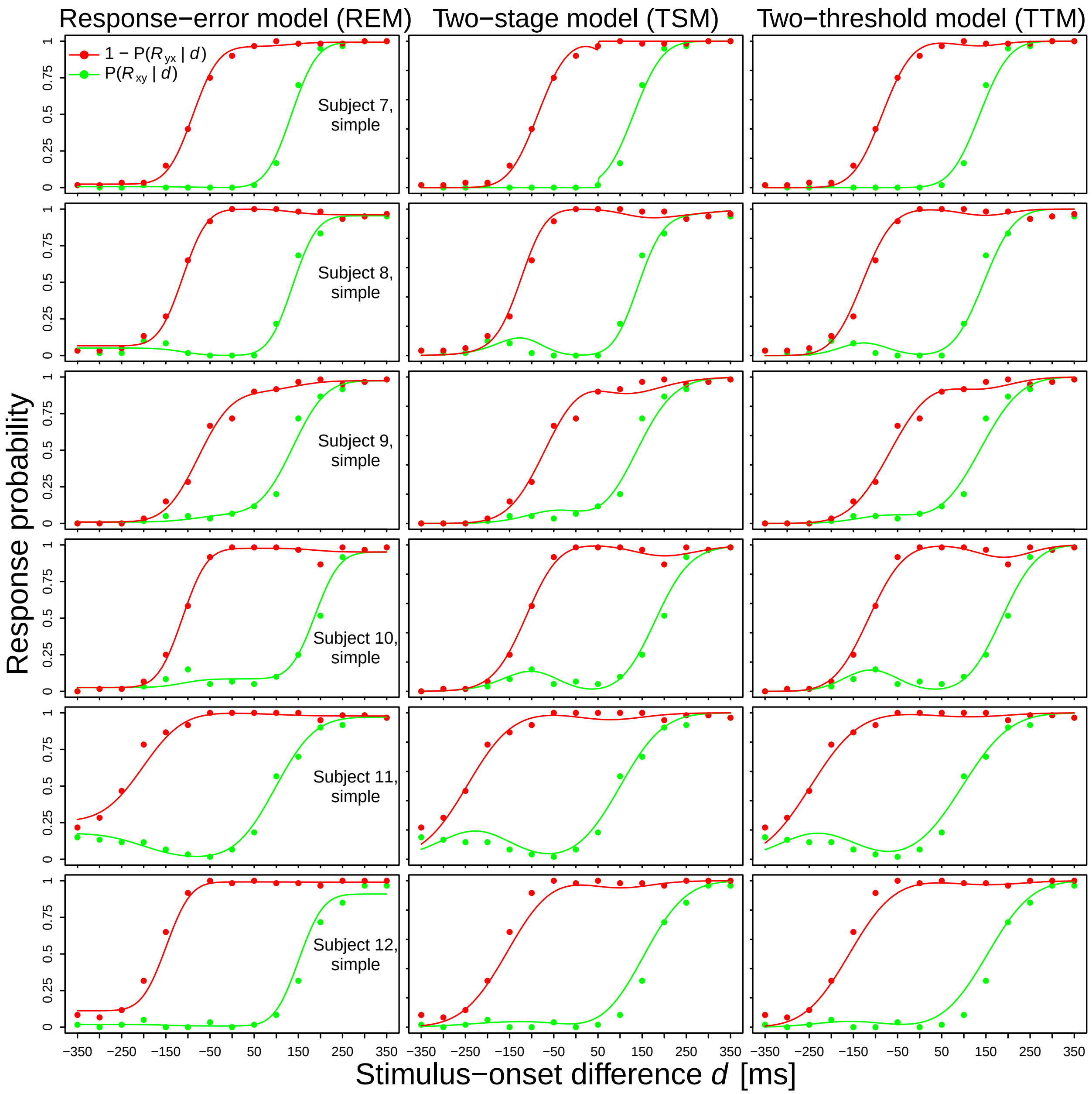

Note. x: visual; y: auditory.

Figure S21

Observed (points) and fitted (lines) psychometric functions $P(R_{xy} \mid d)$ and $1 - P(R_{yx} \mid d)$ for subjects 1–6 with complex stimuli in the study by van Eijk et al. (2008)

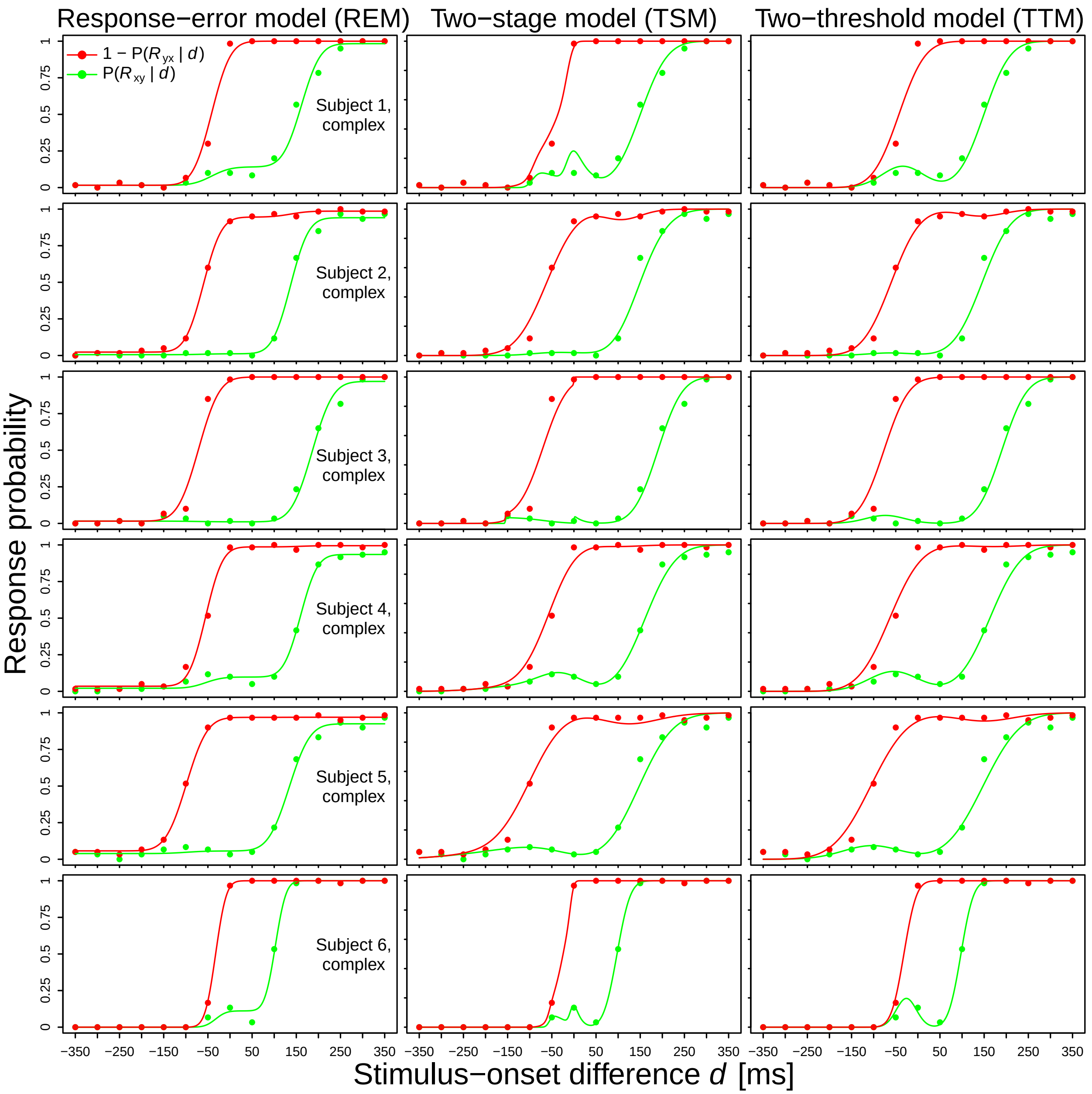

Note. x: visual; y: auditory.

Figure S22

Observed (points) and fitted (lines) psychometric functions $P(R_{xy} \mid d)$ and $1 - P(R_{yx} \mid d)$ for subjects 7–12 with complex stimuli in the study by van Eijk et al. (2008)

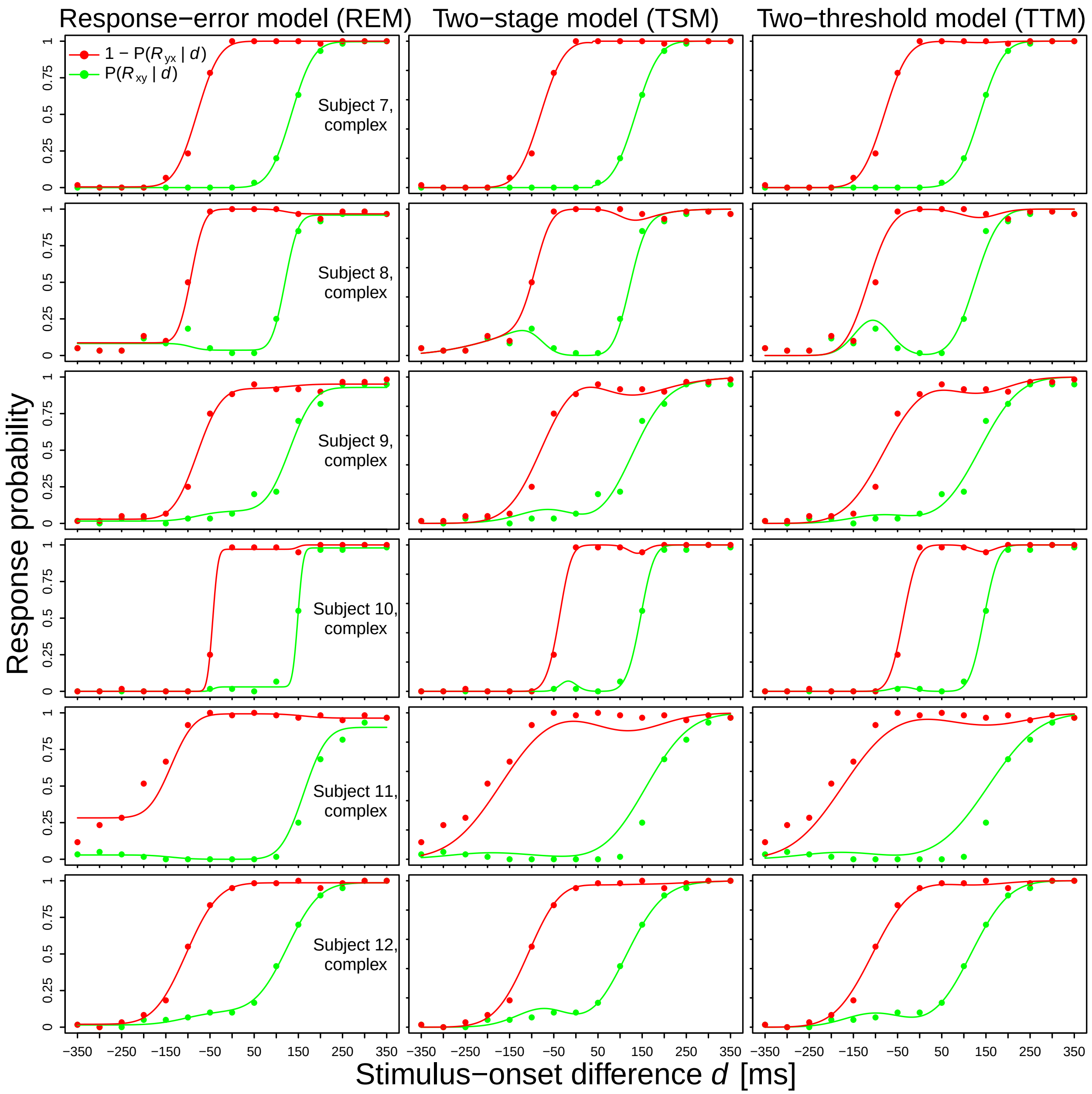

Note. x: visual; y: auditory.

Figure S23

Observed (points) and fitted (lines) psychometric functions $P(R_{xy} \mid d)$ and $1 - P(R_{yx} \mid d)$ for subjects 1–6 in the study by García-Pérez and Alcalá-Quintana (2018)

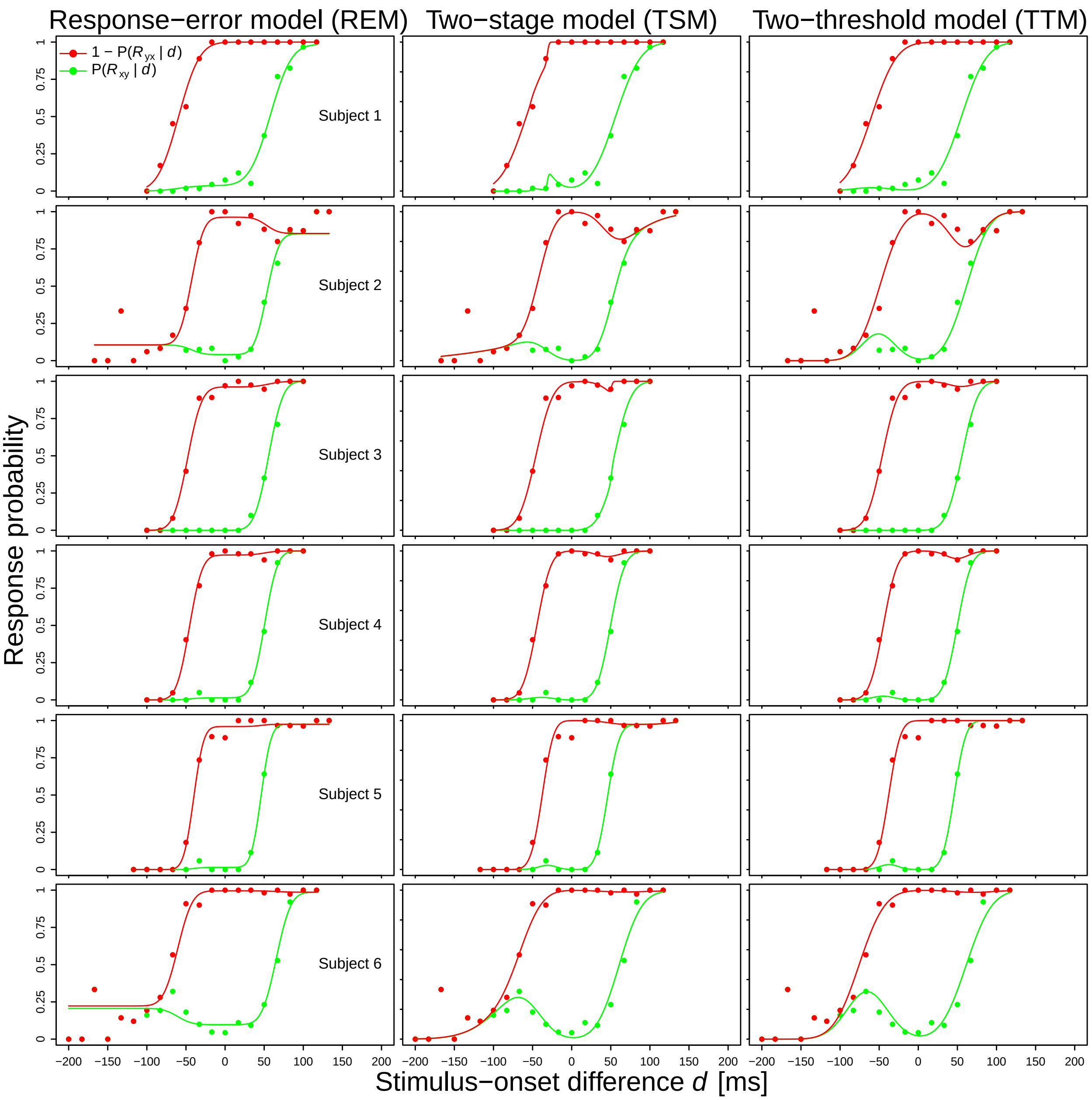

Note. x: right; y: left.

Figure S24

Observed (points) and fitted (lines) psychometric functions $P(R_{xy} \,|\, d)$ and $1 - P(R_{yx} \,|\, d)$ for subjects 7–12 in the study by García-Pérez and Alcalá-Quintana (2018)

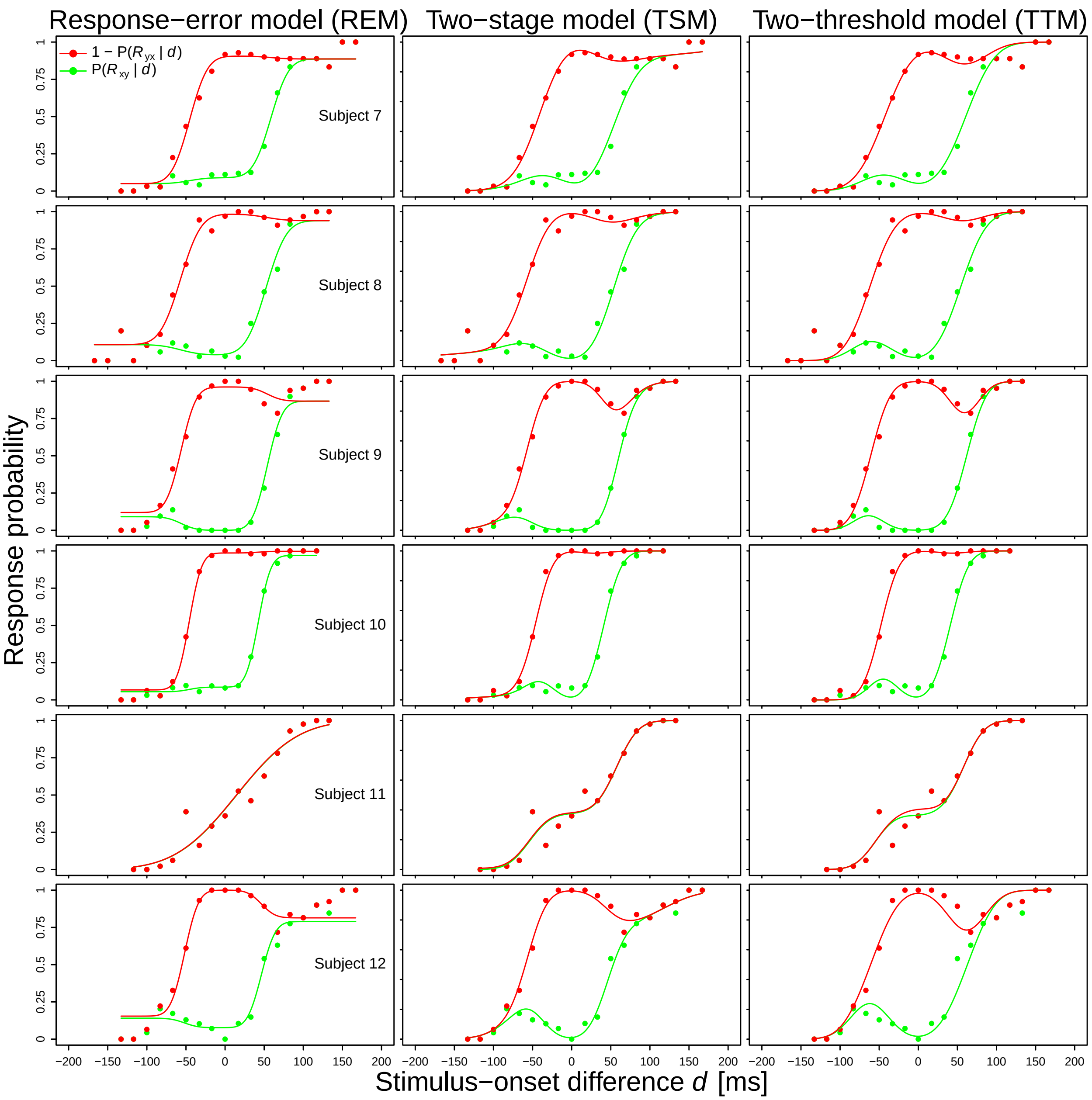

Note. x: right; y: left.

Figure S25

Observed (points) and fitted (lines) psychometric functions $P(R_{xy}\,|\,d)$ and $1 - P(R_{yx}\,|\,d)$ for subjects 13–19 in the study by García-Pérez and Alcalá-Quintana (2018)

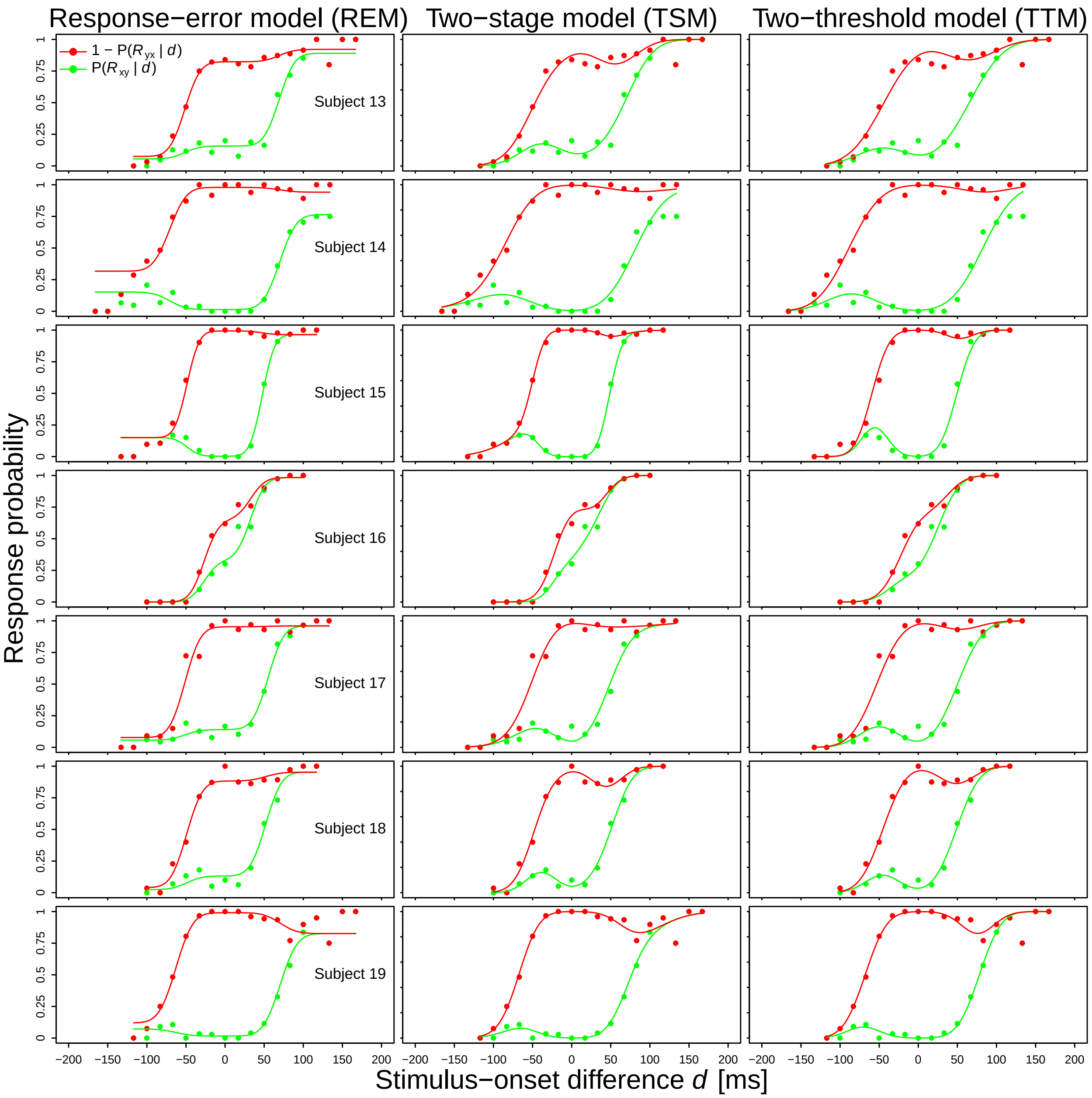

Note. x: right; y: left.

Figure S26

Observed (points) and fitted (lines) psychometric functions $P(R_{xy} \mid d)$ and $1 - P(R_{yx} \mid d)$ for the study by Lahkar et al. (2023)

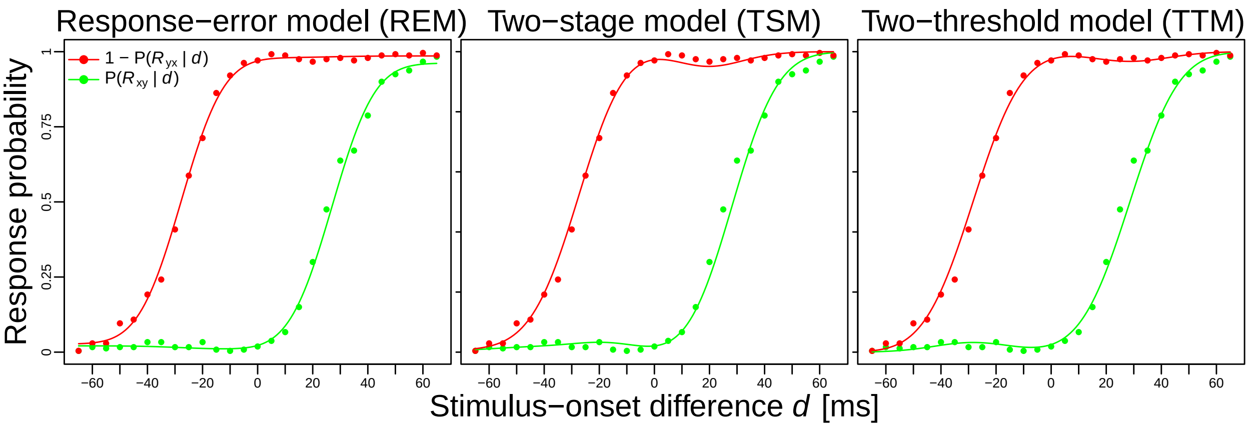

Note. x: right; y: left.

Models assuming Laplace-distributed arrival-latency differences

Representation by three psychometric functions

Figures S27–40 depict the observed and fitted psychometric functions $P(R_{xy} \mid d)$, $P(R_{si} \mid d)$, and $P(R_{yx} \mid d)$ for the models with the alternative assumption of Laplace-distributed arrival-latency differences and all studies considered in the model comparison.

Figure S27

Observed (points) and fitted (lines) psychometric functions $P(R_{xy} \mid d)$, $P(R_{si} \mid d)$, and $P(R_{yx} \mid d)$ for the study by Benussi (1913)

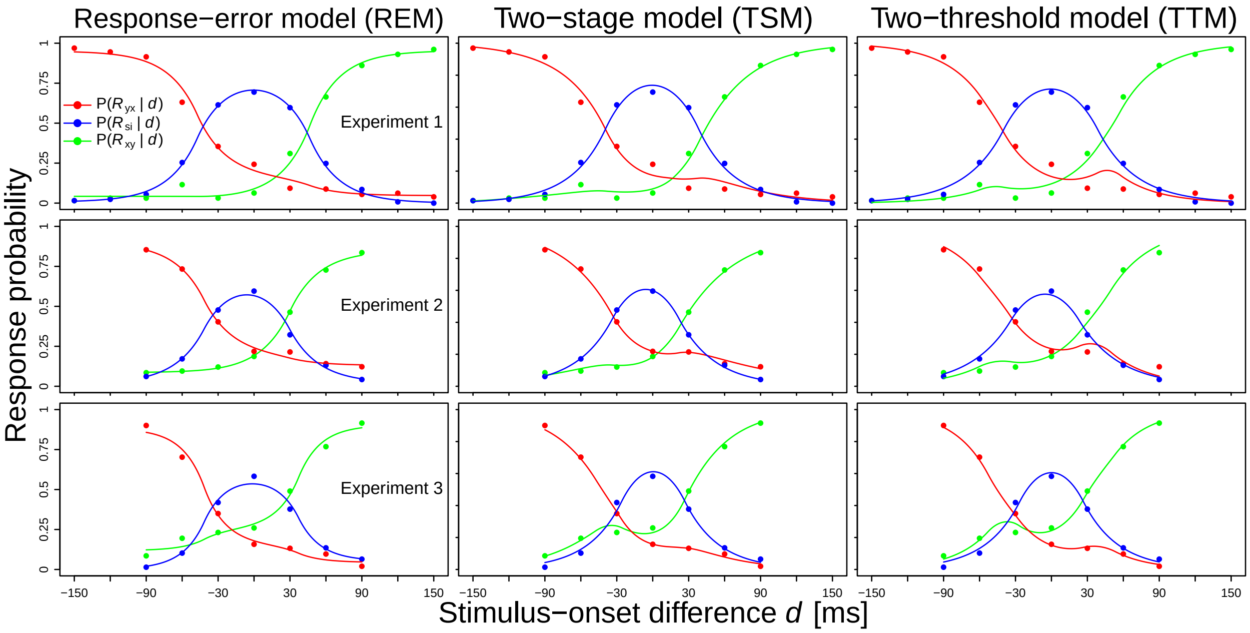

Note. x: left; y: right.

Figure S28

Observed (points) and fitted (lines) psychometric functions $P(R_{xy} \mid d)$, $P(R_{si} \mid d)$, and $P(R_{yx} \mid d)$ for the study by Allan (1975a)

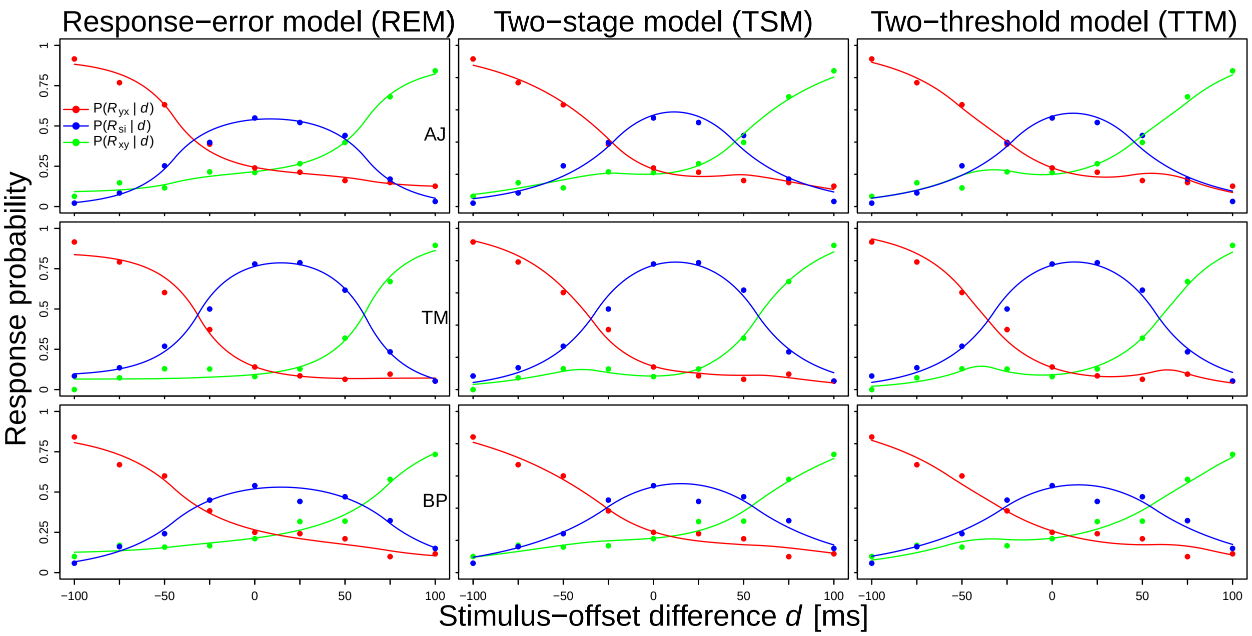

Note. x: visual; y: auditory.

Figure S29

Observed (points) and fitted (lines) psychometric functions $P(R_{xy} \mid d)$, $P(R_{si} \mid d)$, and $P(R_{yx} \mid d)$ for the study by Allan (1975b)

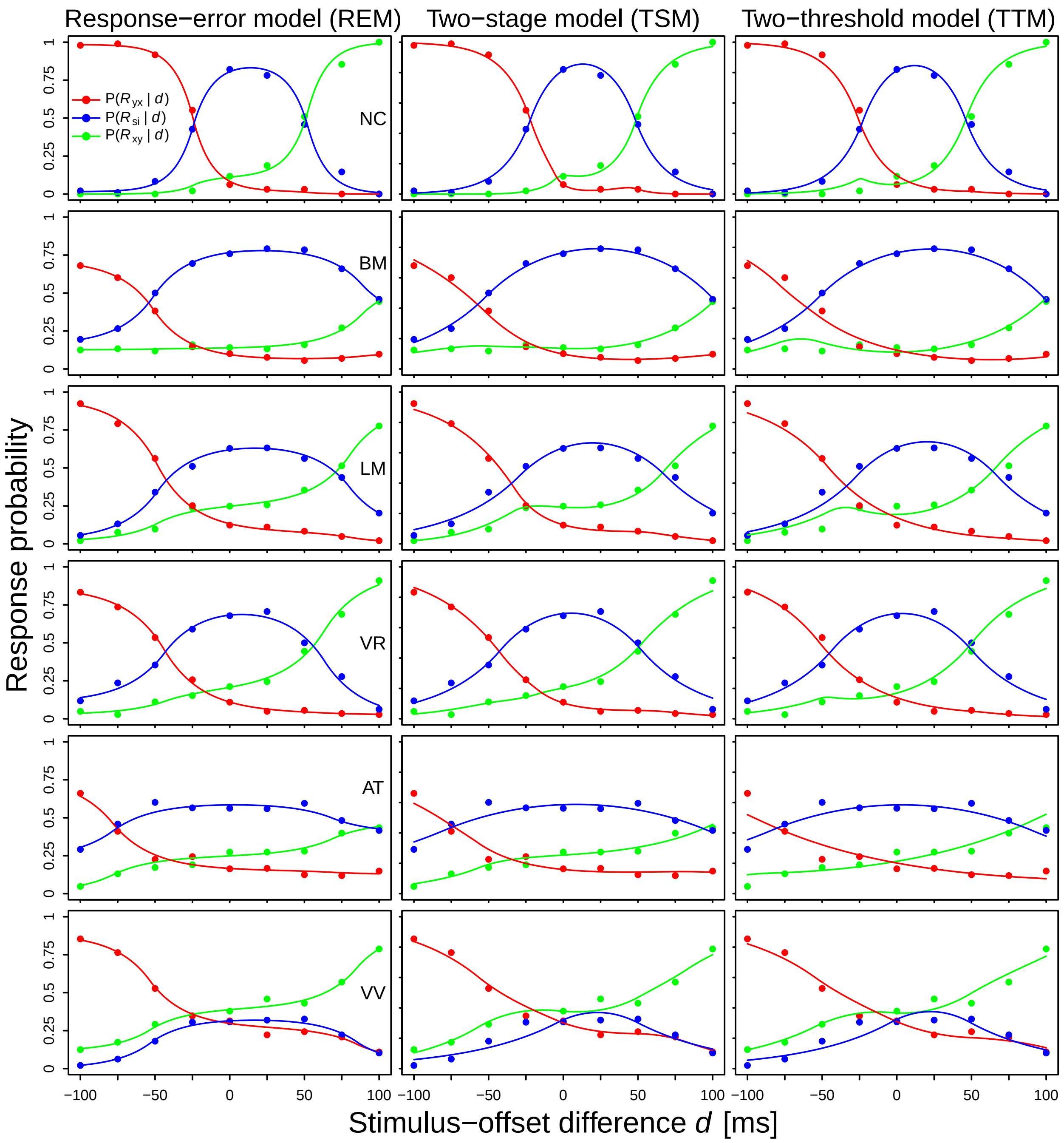

Note. x: visual; y: auditory.

Figure S30

Observed (points) and fitted (lines) psychometric functions $P(R_{xy} \mid d)$, $P(R_{si} \mid d)$, and $P(R_{yx} \mid d)$ for the study by Ulrich (1987)

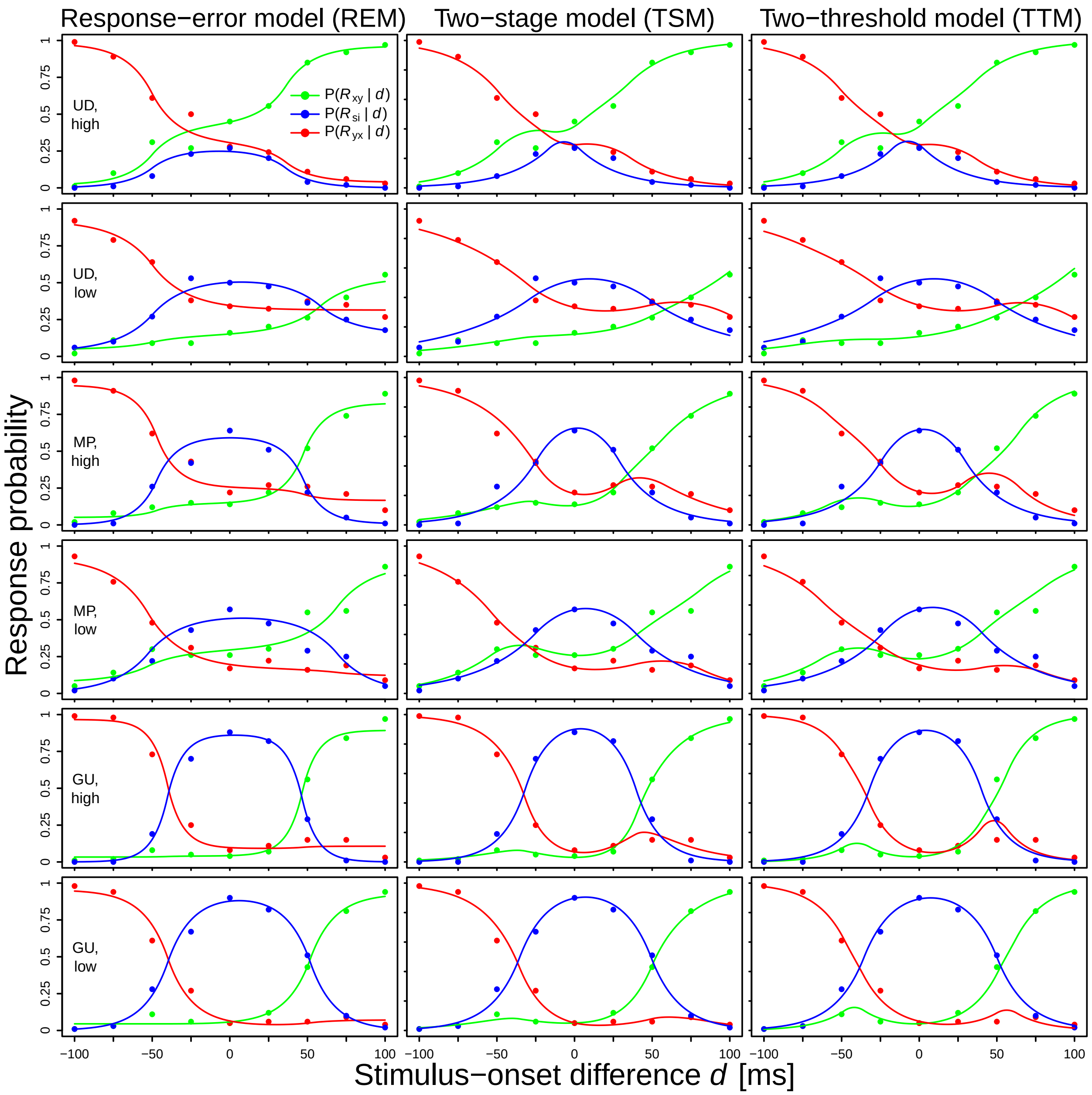

Note. x: top; y: bottom.

Figure S31

Observed (points) and fitted (lines) psychometric functions $P(R_{xy} \mid d)$, $P(R_{si} \mid d)$, and $P(R_{yx} \mid d)$ for the subjects PJ and PT in the study by Jaśkowski (1991a, Experiment 3)

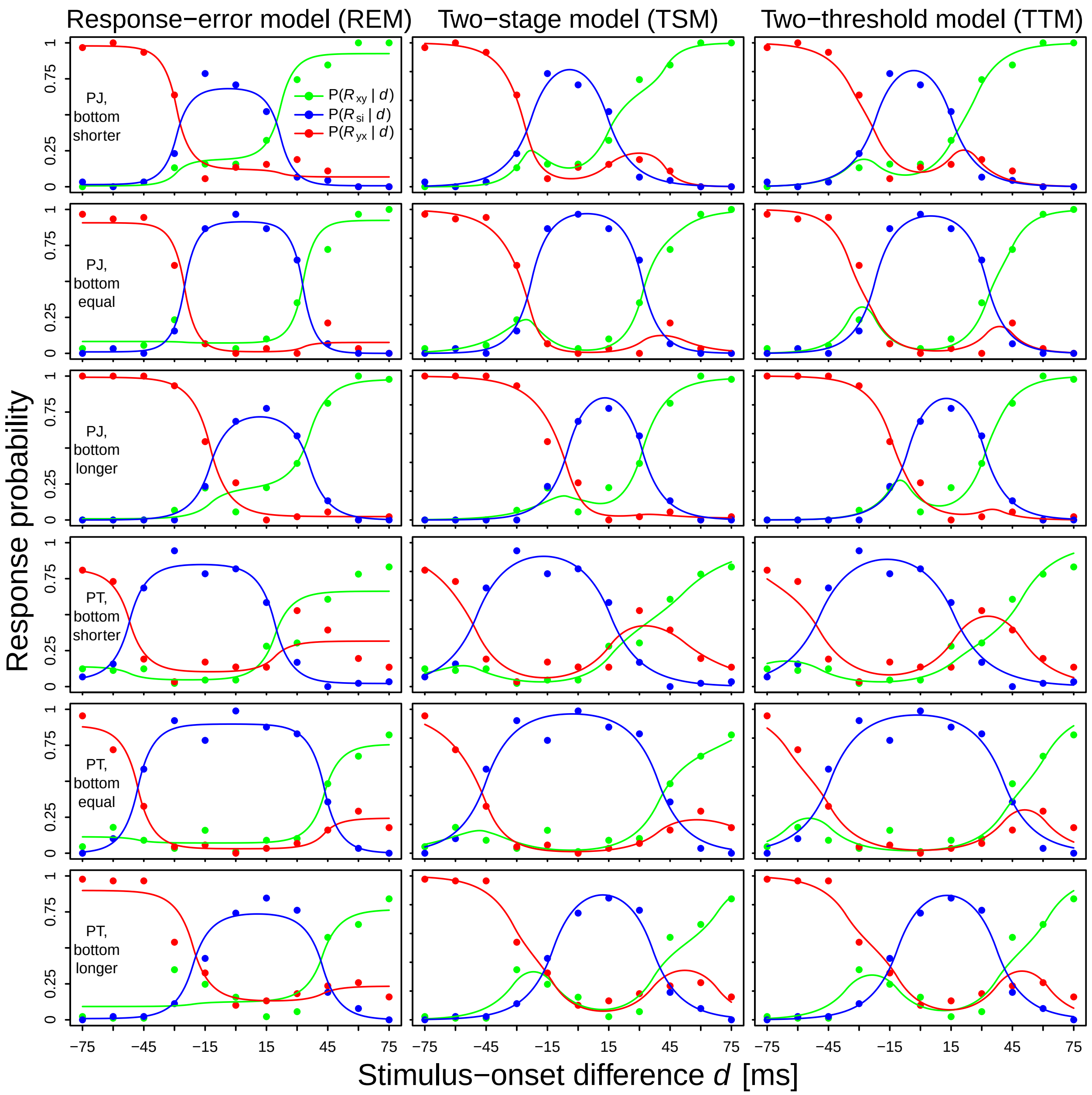

Note. x: bottom; y: top.

Figure S32

Observed (points) and fitted (lines) psychometric functions $P(R_{xy} \,|\, d)$, $P(R_{si} \,|\, d)$, and $P(R_{yx} \,|\, d)$ for the subject WW in the study by Jaśkowski (1991a, Experiment 3)

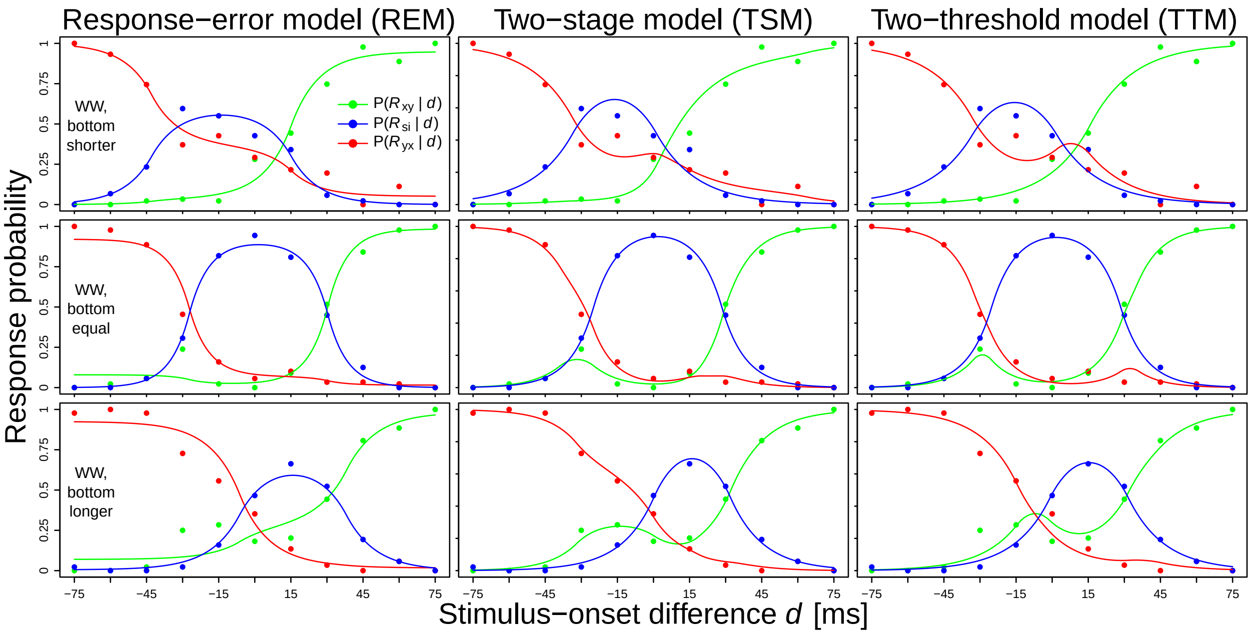

Note. x: bottom; y: top.

Figure S33

Observed (points) and fitted (lines) psychometric functions $P(R_{xy} \mid d)$, $P(R_{si} \mid d)$, and $P(R_{yx} \mid d)$ for subjects 1–4 and 6 with simple stimuli in the study by van Eijk et al. (2008)

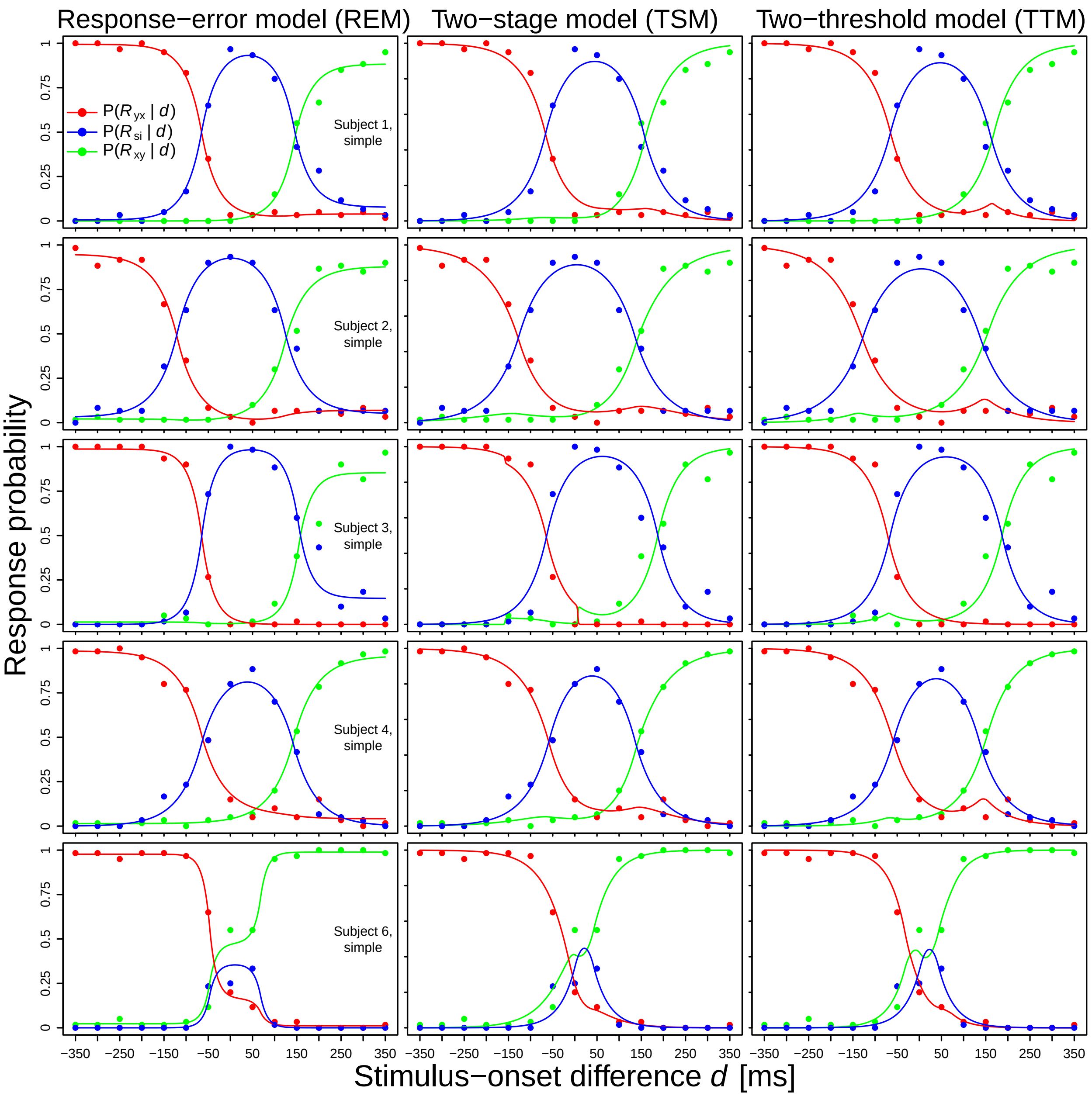

Note. x: visual; y: auditory.

Figure S34

Observed (points) and fitted (lines) psychometric functions $P(R_{xy} \mid d)$, $P(R_{si} \mid d)$, and $P(R_{yx} \mid d)$ for subjects 7–12 with simple stimuli in the study by van Eijk et al. (2008)

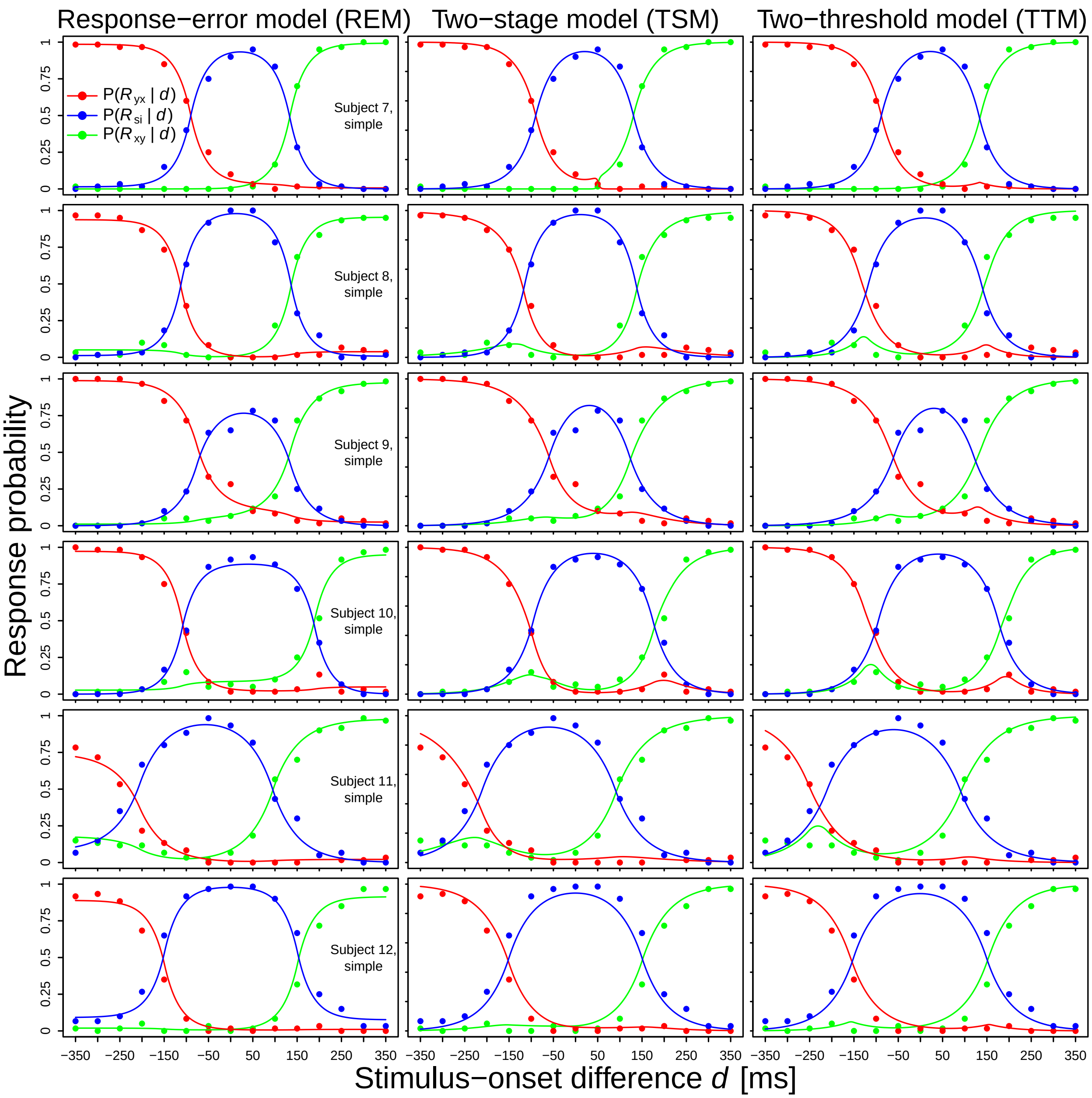

Note. x: visual; y: auditory.

Figure S35

Observed (points) and fitted (lines) psychometric functions $P(R_{xy} \mid d)$, $P(R_{si} \mid d)$, and $P(R_{yx} \mid d)$ for subjects 1–6 with complex stimuli in the study by van Eijk et al. (2008)

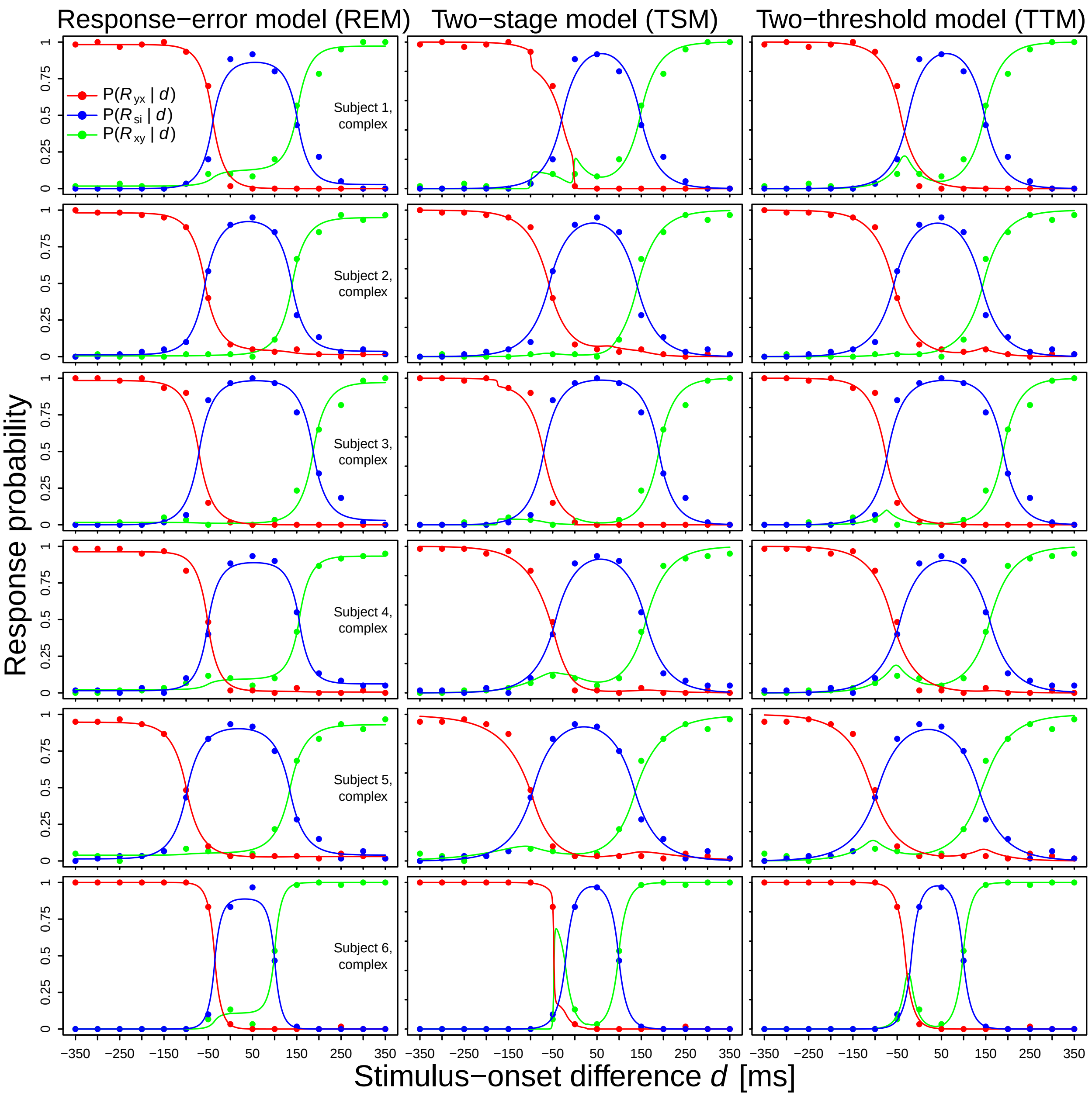

Note. x: visual; y: auditory.

Figure S36

Observed (points) and fitted (lines) psychometric functions $P(R_{xy} \mid d)$, $P(R_{si} \mid d)$, and $P(R_{yx} \mid d)$ for subjects 7–12 with complex stimuli in the study by van Eijk et al. (2008)

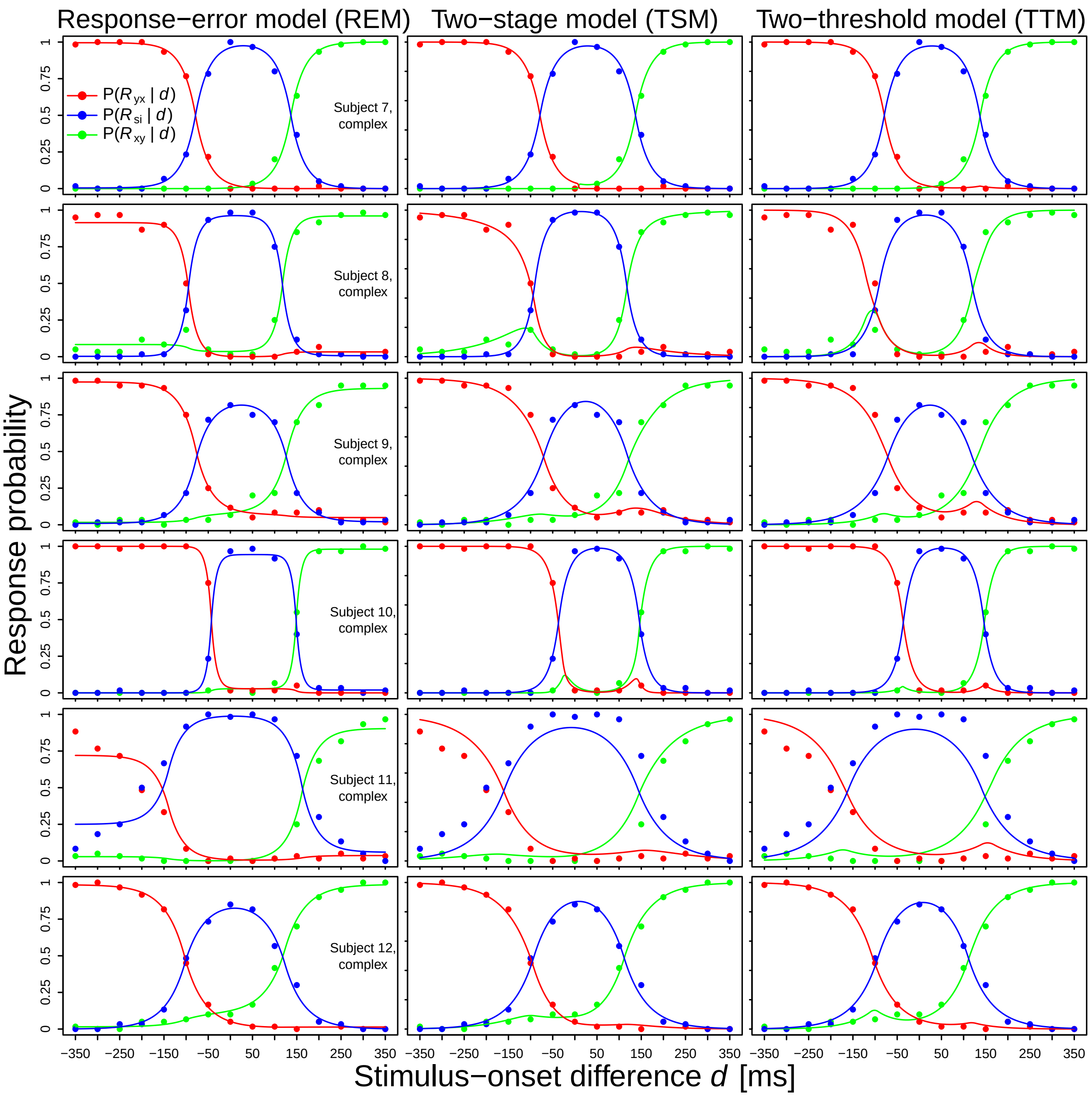

Note. x: visual; y: auditory.

Figure S37

Observed (points) and fitted (lines) psychometric functions $P(R_{xy} \mid d)$, $P(R_{si} \mid d)$, and $P(R_{yx} \mid d)$ for subjects 1–6 in the study by García-Pérez and Alcalá-Quintana (2018)

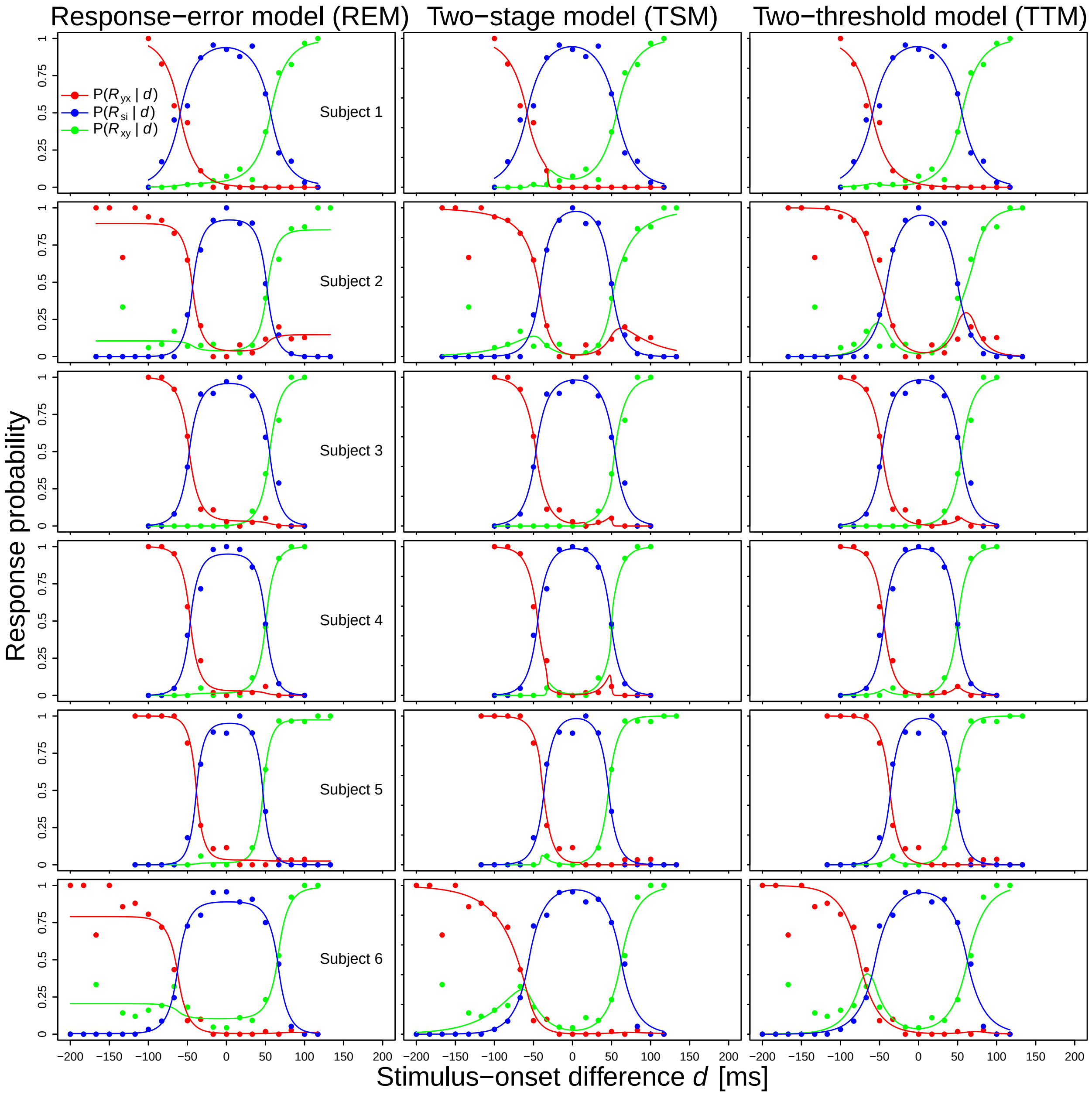

Note. x: right; y: left.

Figure S38

Observed (points) and fitted (lines) psychometric functions $P(R_{xy} \mid d)$, $P(R_{si} \mid d)$, and $P(R_{yx} \mid d)$ for subjects 7–12 in the study by García-Pérez and Alcalá-Quintana (2018)

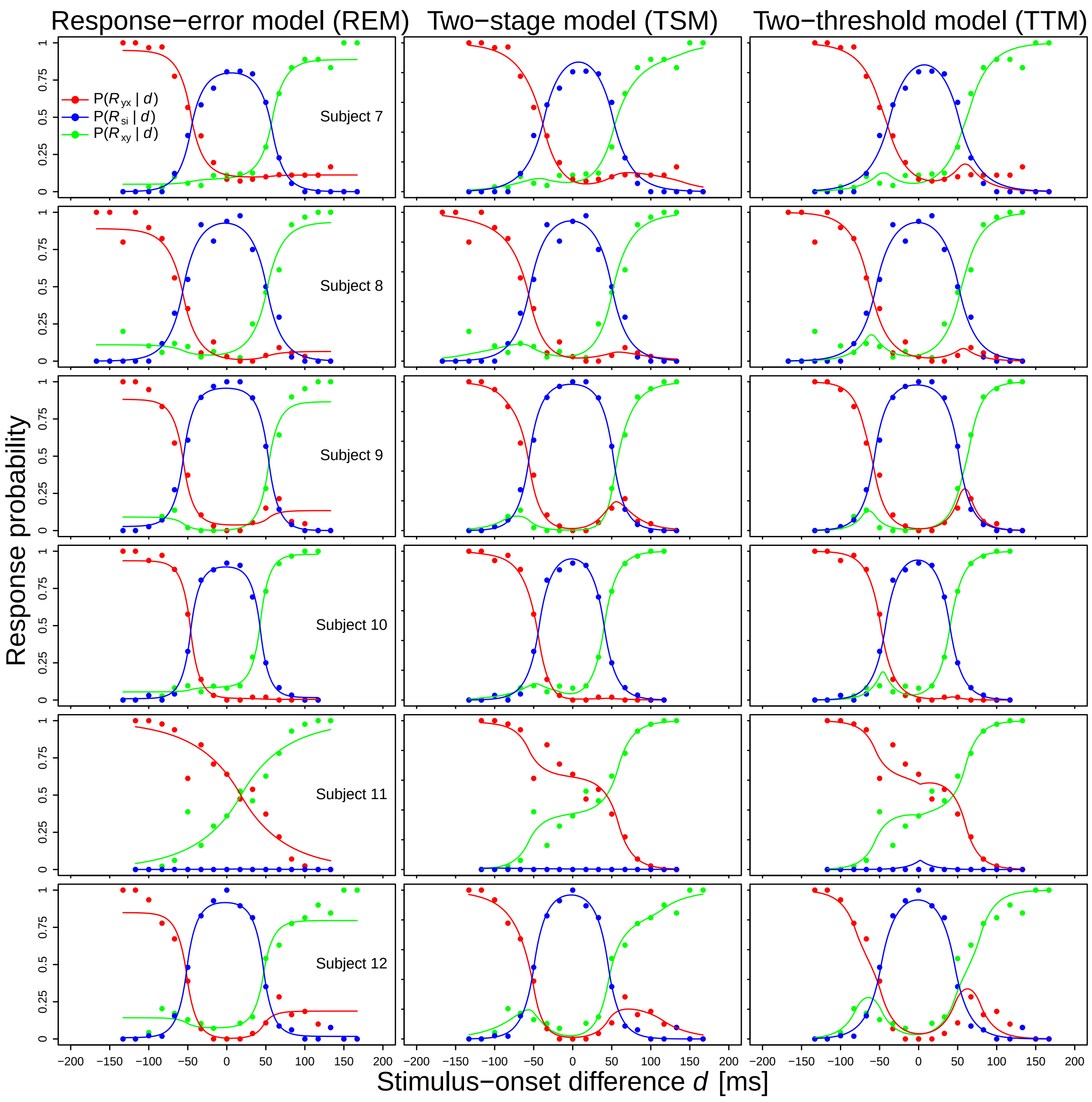

Note. x: right; y: left.

Figure S39

Observed (points) and fitted (lines) psychometric functions $P(R_{xy} \mid d)$, $P(R_{si} \mid d)$, and $P(R_{yx} \mid d)$ for subjects 13–19 in the study by García-Pérez and Alcalá-Quintana (2018)

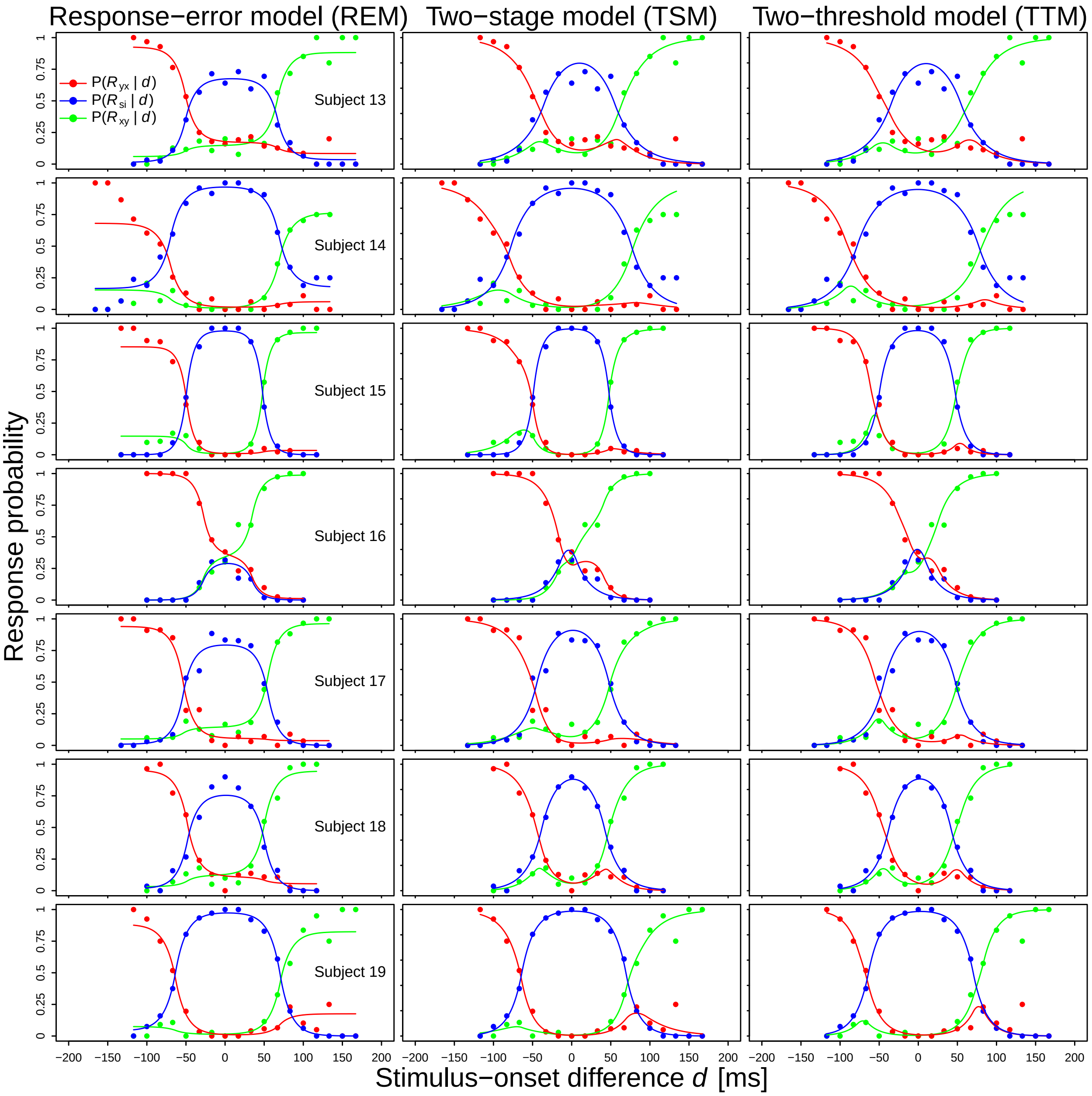

Note. x: right; y: left.

Figure S40

Observed (points) and fitted (lines) psychometric functions $P(R_{xy} \mid d)$, $P(R_{si} \mid d)$, and $P(R_{yx} \mid d)$ for the study by Lahkar et al. (2023)

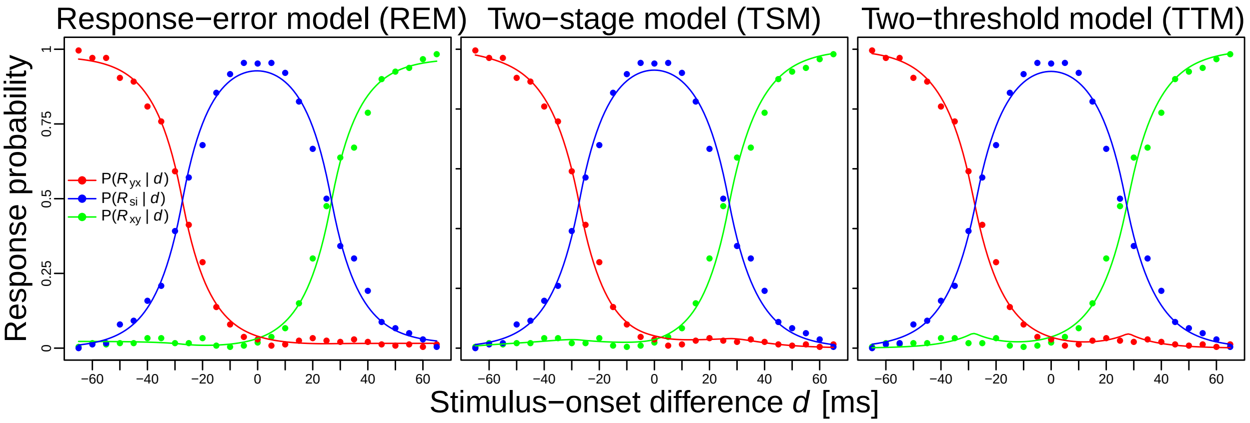

Note. x: right; y: left.

Representation by two psychometric functions

Figures S41–54 depict the observed and fitted psychometric functions $P(R_{xy} \mid d)$ and $1 - P(R_{yx} \mid d)$ for the models with the alternative assumption of Laplace-distributed arrival-latency differences and all studies considered in the model comparison.

Figure S41

Observed (points) and fitted (lines) psychometric functions $P(R_{xy} \mid d)$ and $1 - P(R_{yx} \mid d)$ for the study by Benussi (1913)

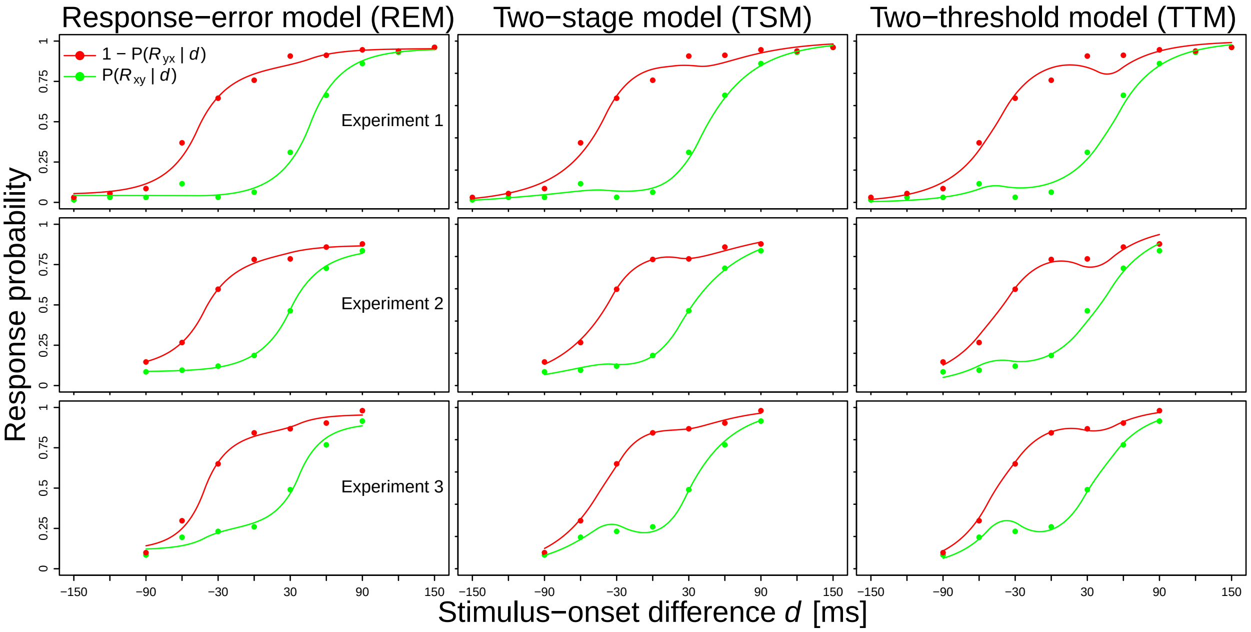

Note. x: left; y: right.

Figure S42

Observed (points) and fitted (lines) psychometric functions $P(R_{xy} \mid d)$ *and* $1 - P(R_{yx} \mid d)$ *for the study by Allan (1975a)*

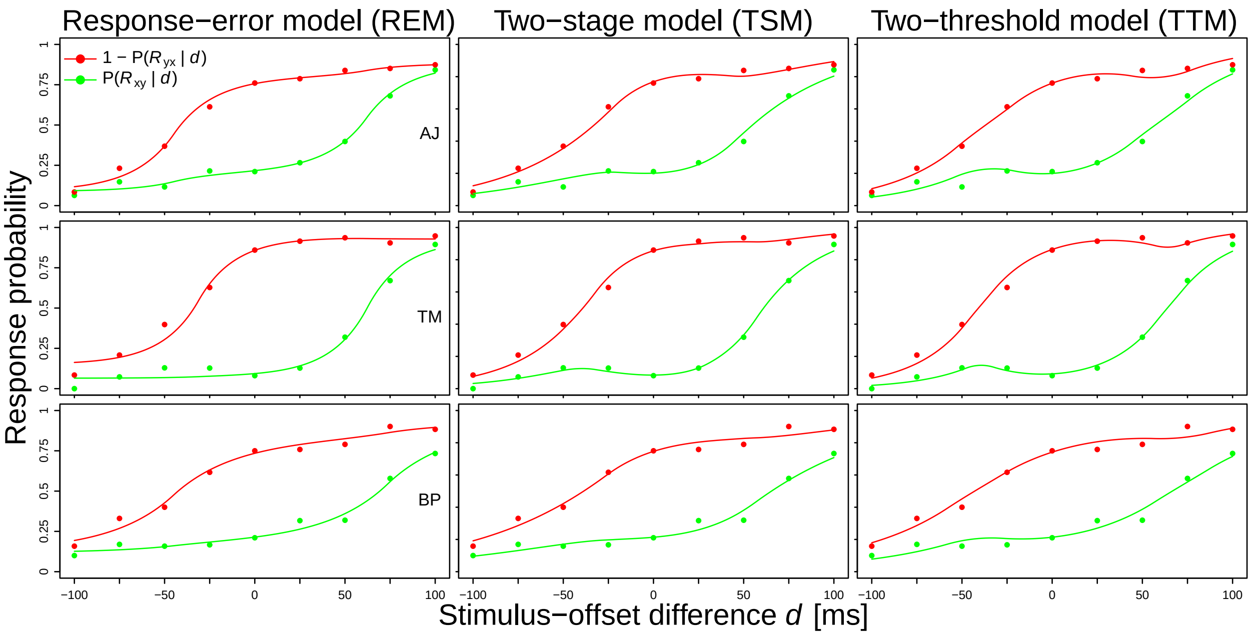

Note. x: visual; y: auditory.

Figure S43

Observed (points) and fitted (lines) psychometric functions $P(R_{xy} \mid d)$ and $1 - P(R_{yx} \mid d)$ for the study by Allan (1975b)

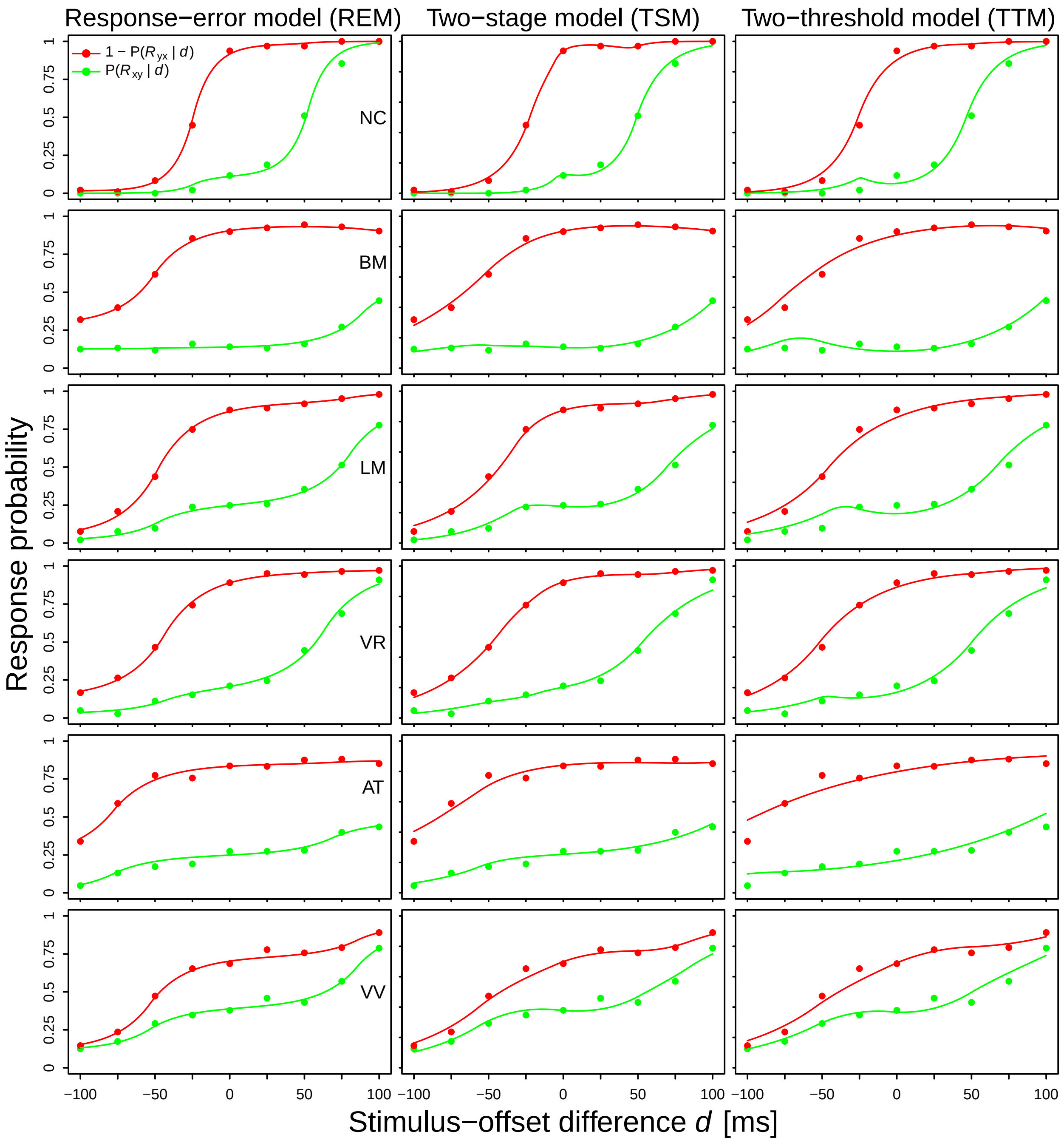

Note. x: right; y: left.

Figure S44

Observed (points) and fitted (lines) psychometric functions $P(R_{xy} \mid d)$ and $1 - P(R_{yx} \mid d)$ for the study by Ulrich (1987)

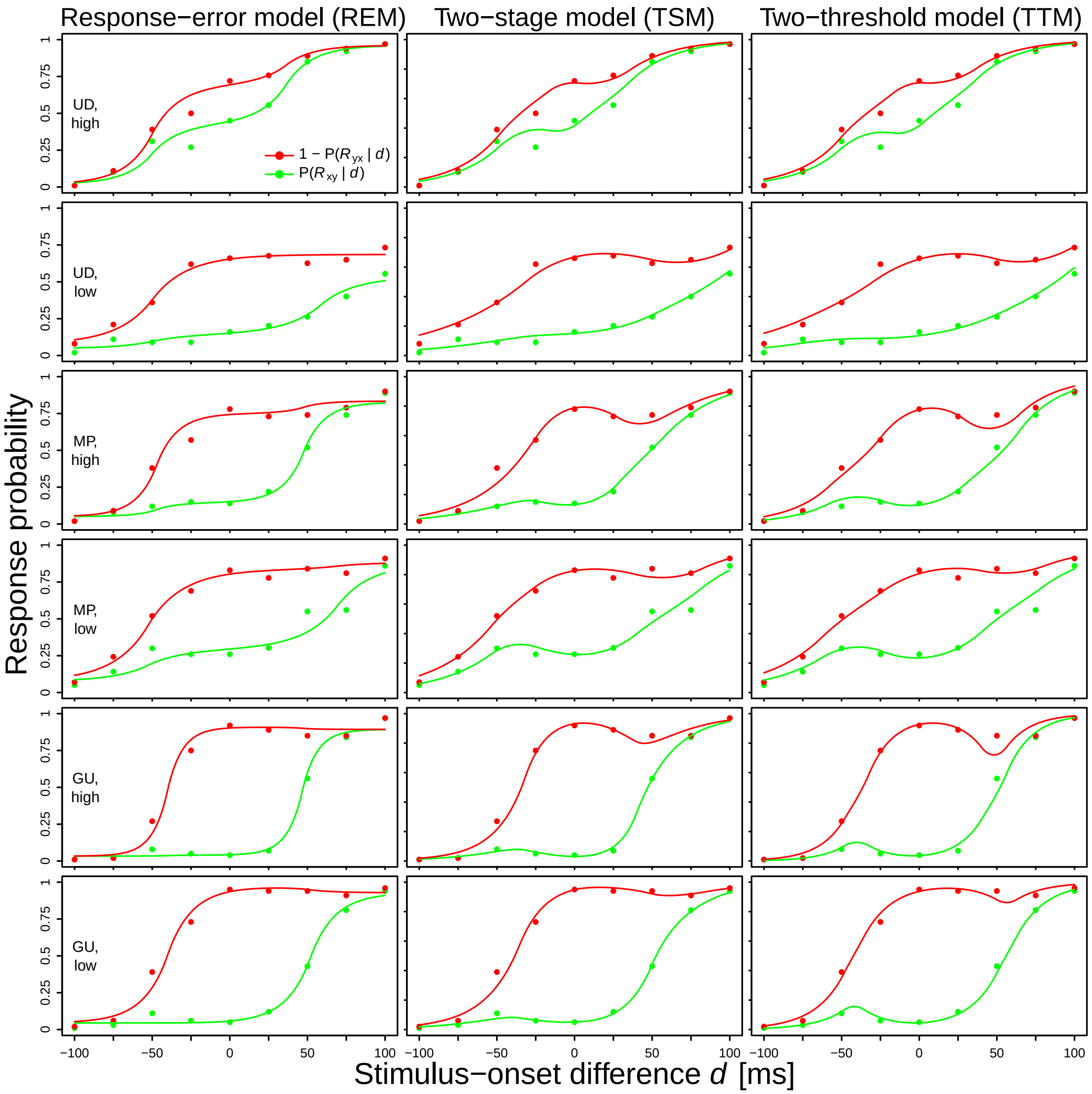

Note. x: top; y: bottom.

Figure S45

Observed (points) and fitted (lines) psychometric functions $P(R_{xy} \mid d)$ and $1 - P(R_{yx} \mid d)$ for the subjects PJ and PT in the study by Jaśkowski (1991a, Experiment 3)

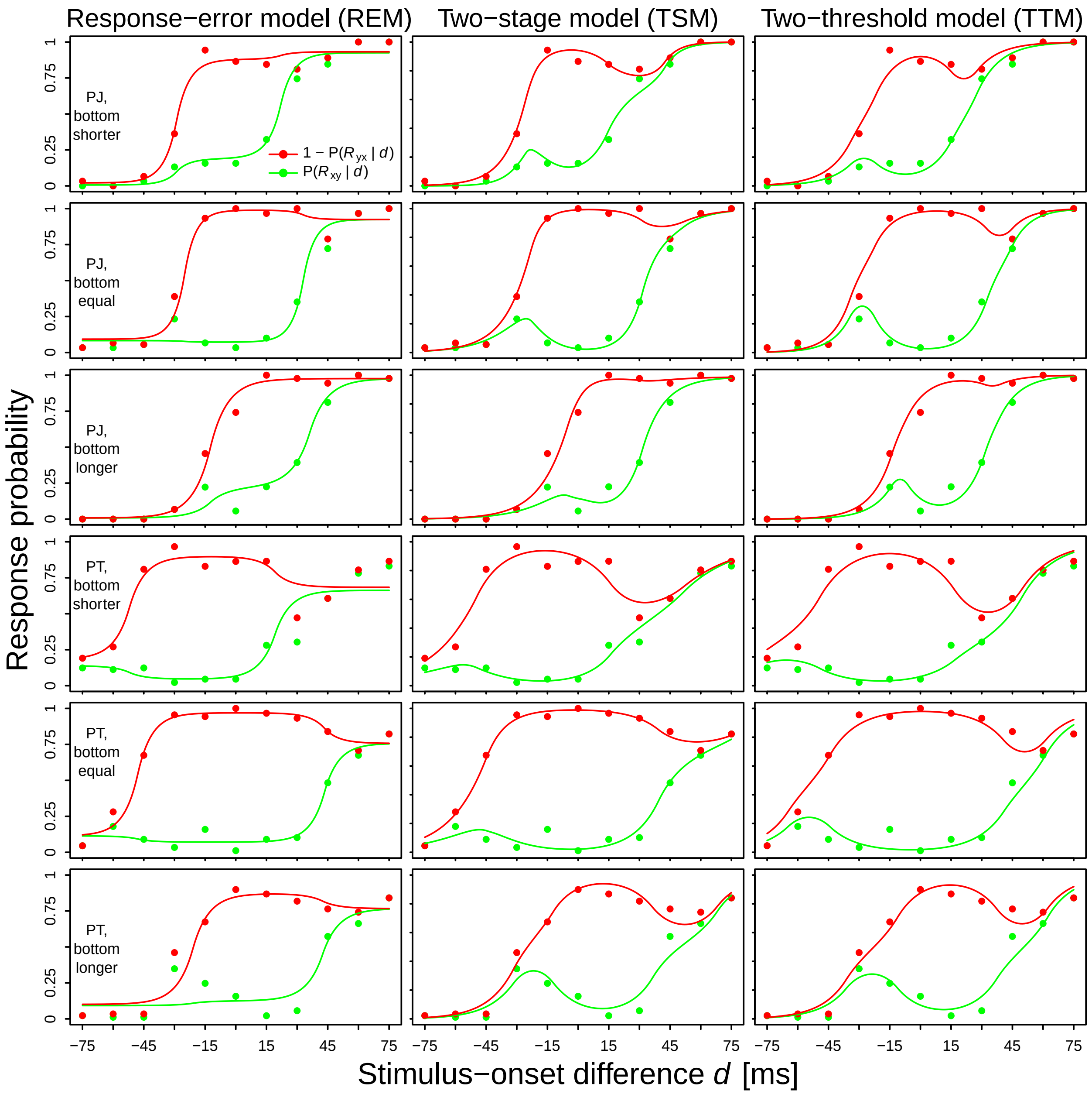

Note. x: bottom; y: top.

Figure S46

Observed (points) and fitted (lines) psychometric functions $P(R_{xy} \mid d)$ and $1 - P(R_{yx} \mid d)$ for the subject WW in the study by Jaśkowski (1991a, Experiment 3)

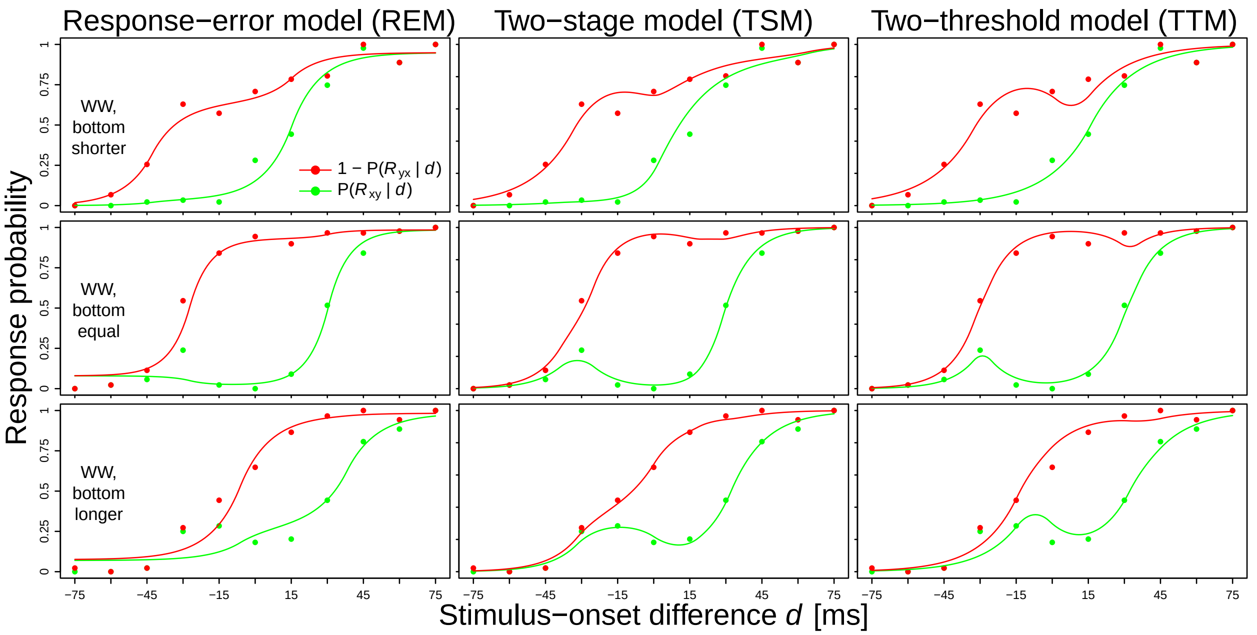

Note. x: bottom; y: top.

Figure S47

Observed (points) and fitted (lines) psychometric functions $P(R_{xy} \mid d)$ and $1 - P(R_{yx} \mid d)$ for subjects 1–4 and 6 with simple stimuli in the study by van Eijk et al. (2008)

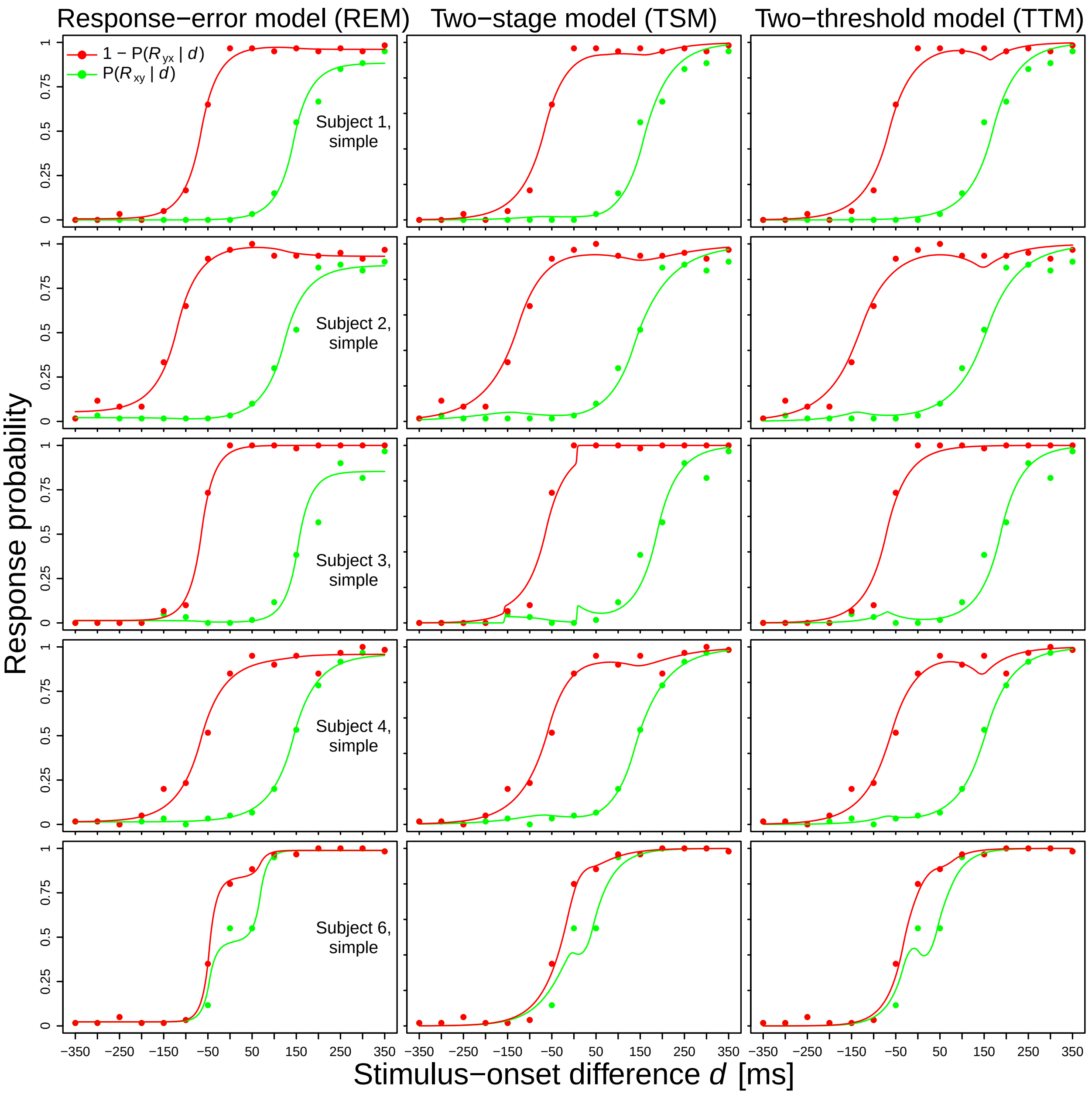

Note. x: visual; y: auditory.

Figure S48

Observed (points) and fitted (lines) psychometric functions $P(R_{xy} \mid d)$ and $1 - P(R_{yx} \mid d)$ for subjects 7–12 with simple stimuli in the study by van Eijk et al. (2008)

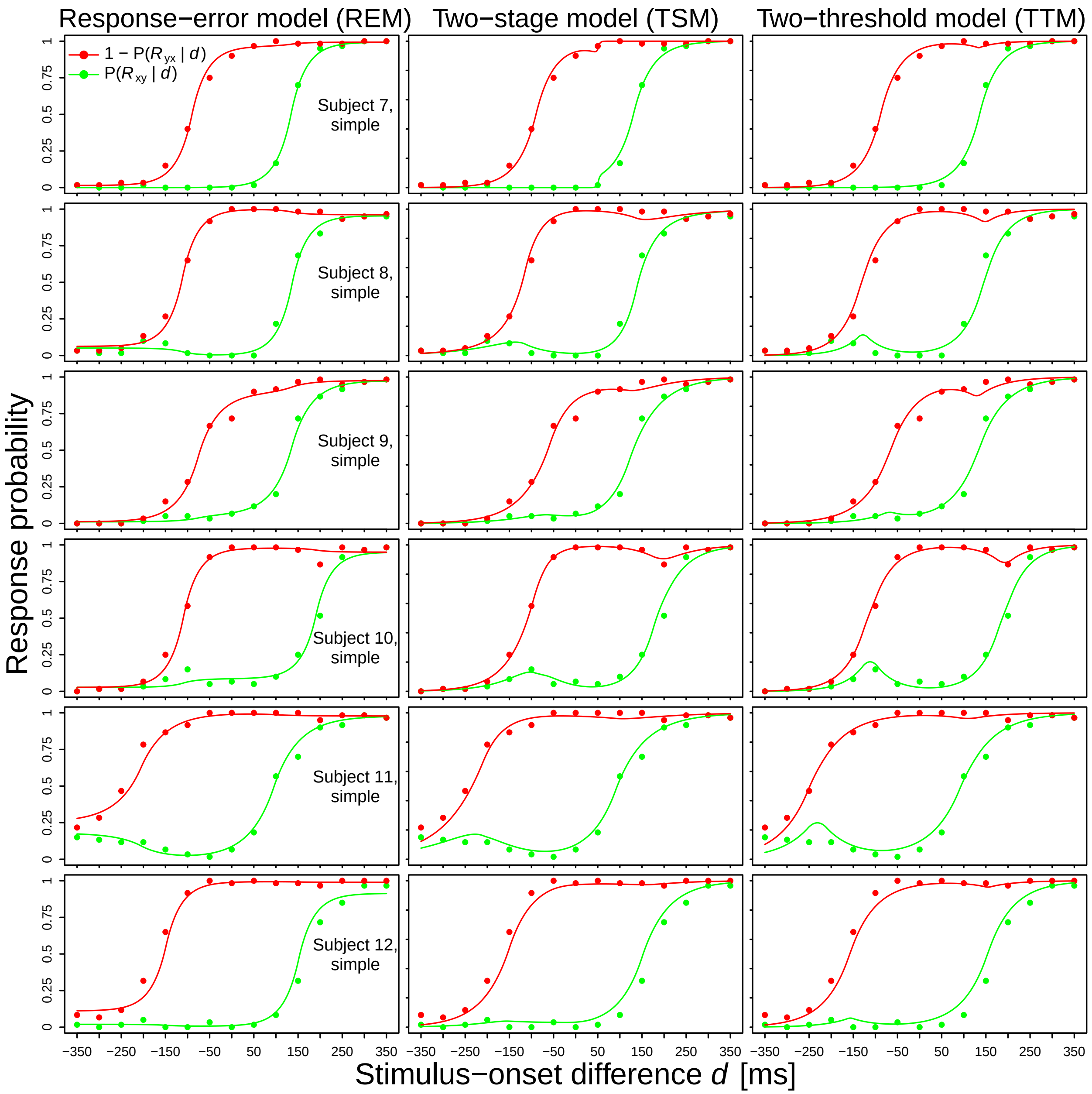

Note. x: visual; y: auditory.

Figure S49

Observed (points) and fitted (lines) psychometric functions $P(R_{xy} \mid d)$ and $1 - P(R_{yx} \mid d)$ for subjects 1–6 with complex stimuli in the study by van Eijk et al. (2008)

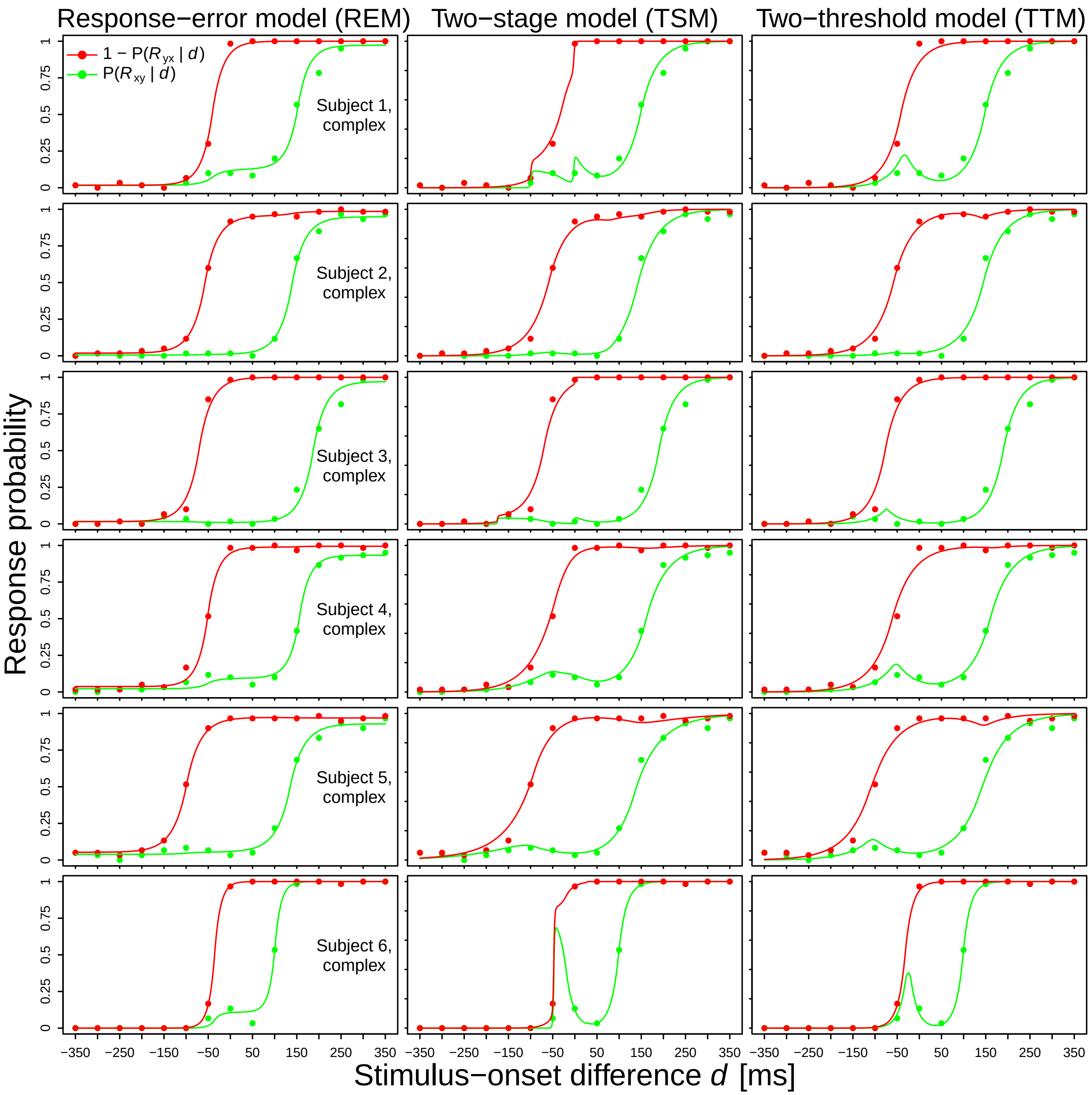

Note. x: visual; y: auditory.

Figure S50

Observed (points) and fitted (lines) psychometric functions $P(R_{xy} \mid d)$ and $1 - P(R_{yx} \mid d)$ for subjects 7–12 with complex stimuli in the study by van Eijk et al. (2008)

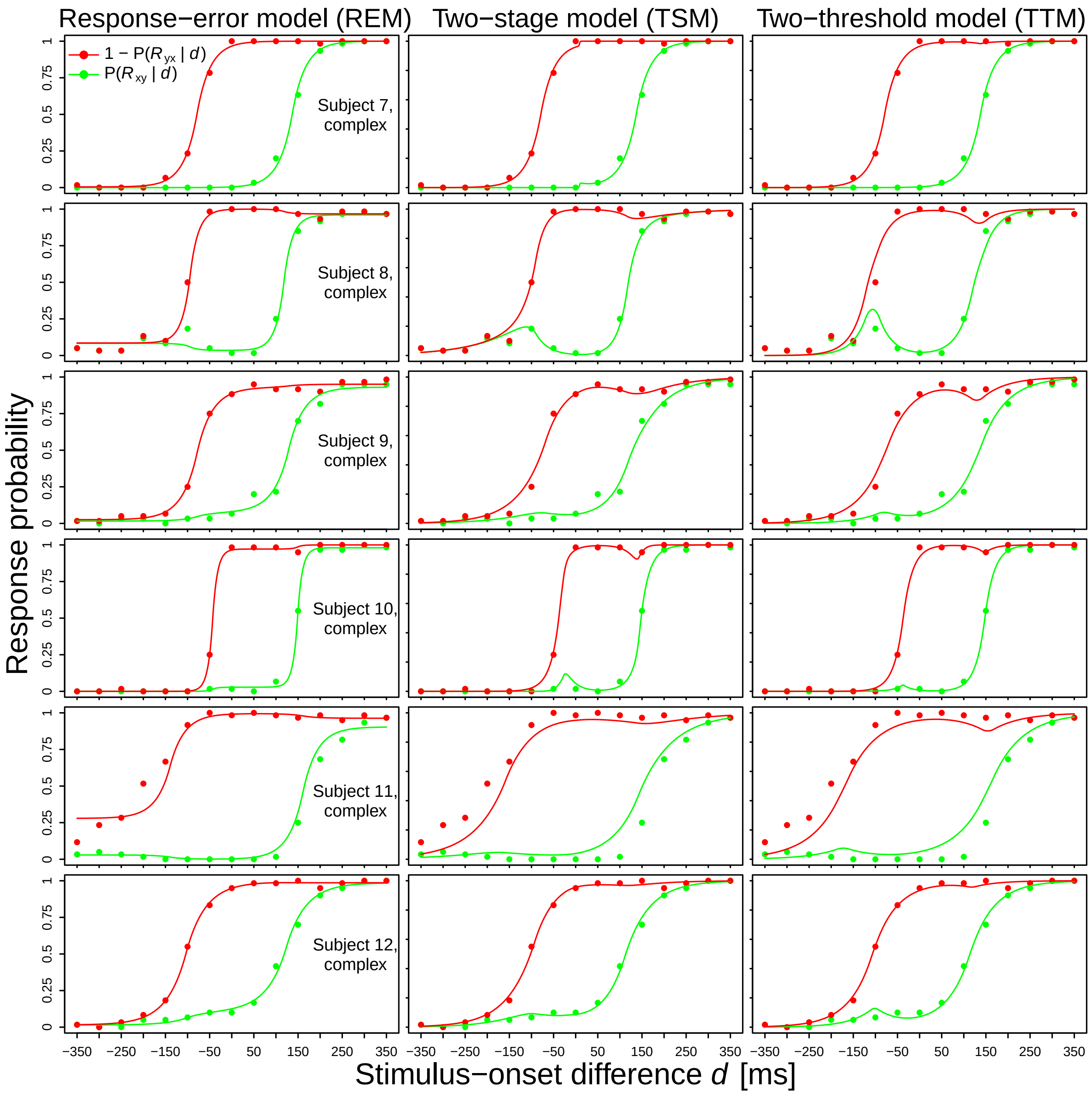

Note. x: visual; y: auditory.

Figure S51

Observed (points) and fitted (lines) psychometric functions $P(R_{xy}\,|\,d)$ and $1 - P(R_{yx}\,|\,d)$ for subjects 1–6 in the study by García-Pérez and Alcalá-Quintana (2018)

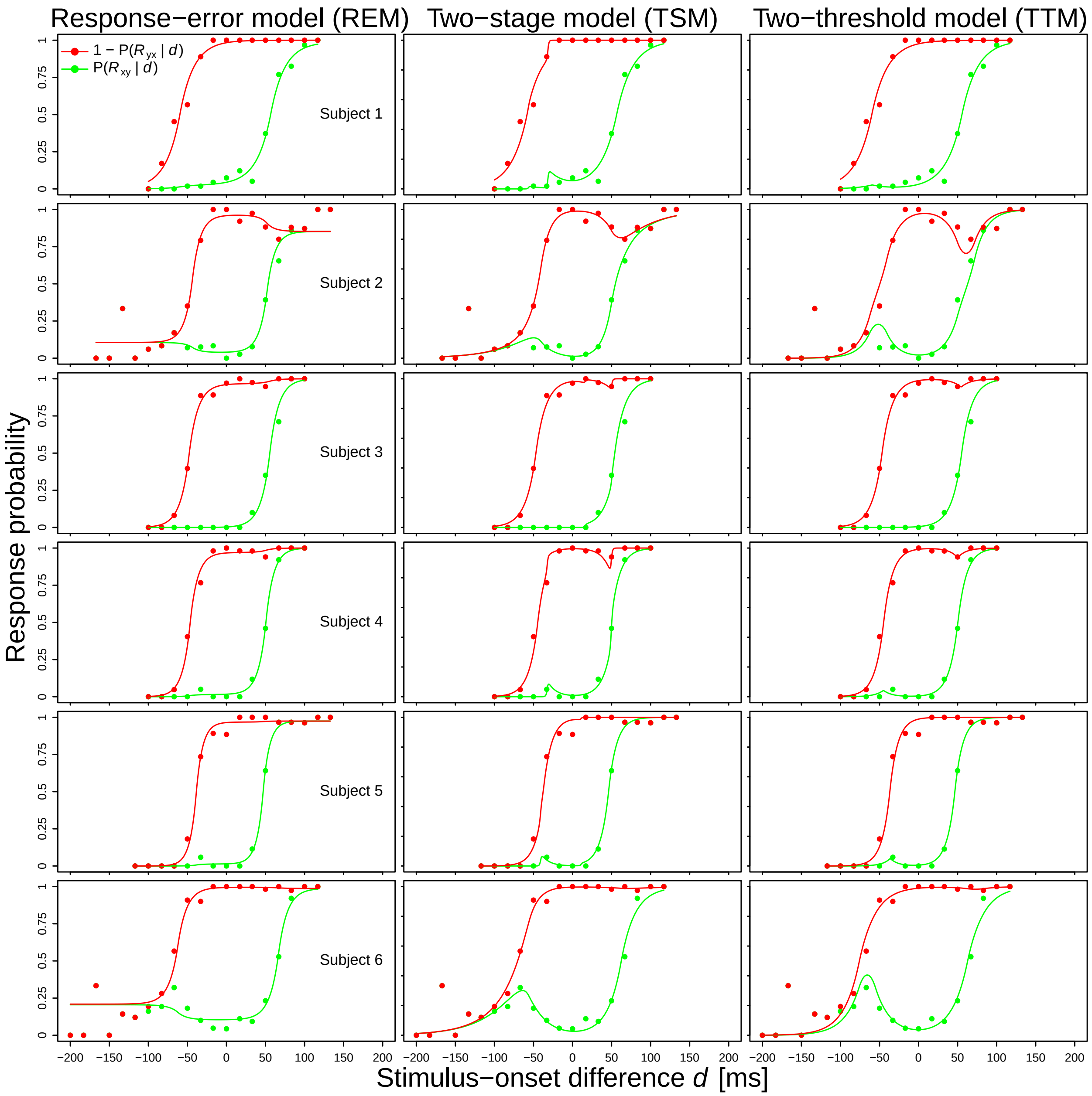

Note. x: right; y: left.

Figure S52

Observed (points) and fitted (lines) psychometric functions $P(R_{xy}\,|\,d)$ and $1 - P(R_{yx}\,|\,d)$ for subjects 7–12 in the study by García-Pérez and Alcalá-Quintana (2018)

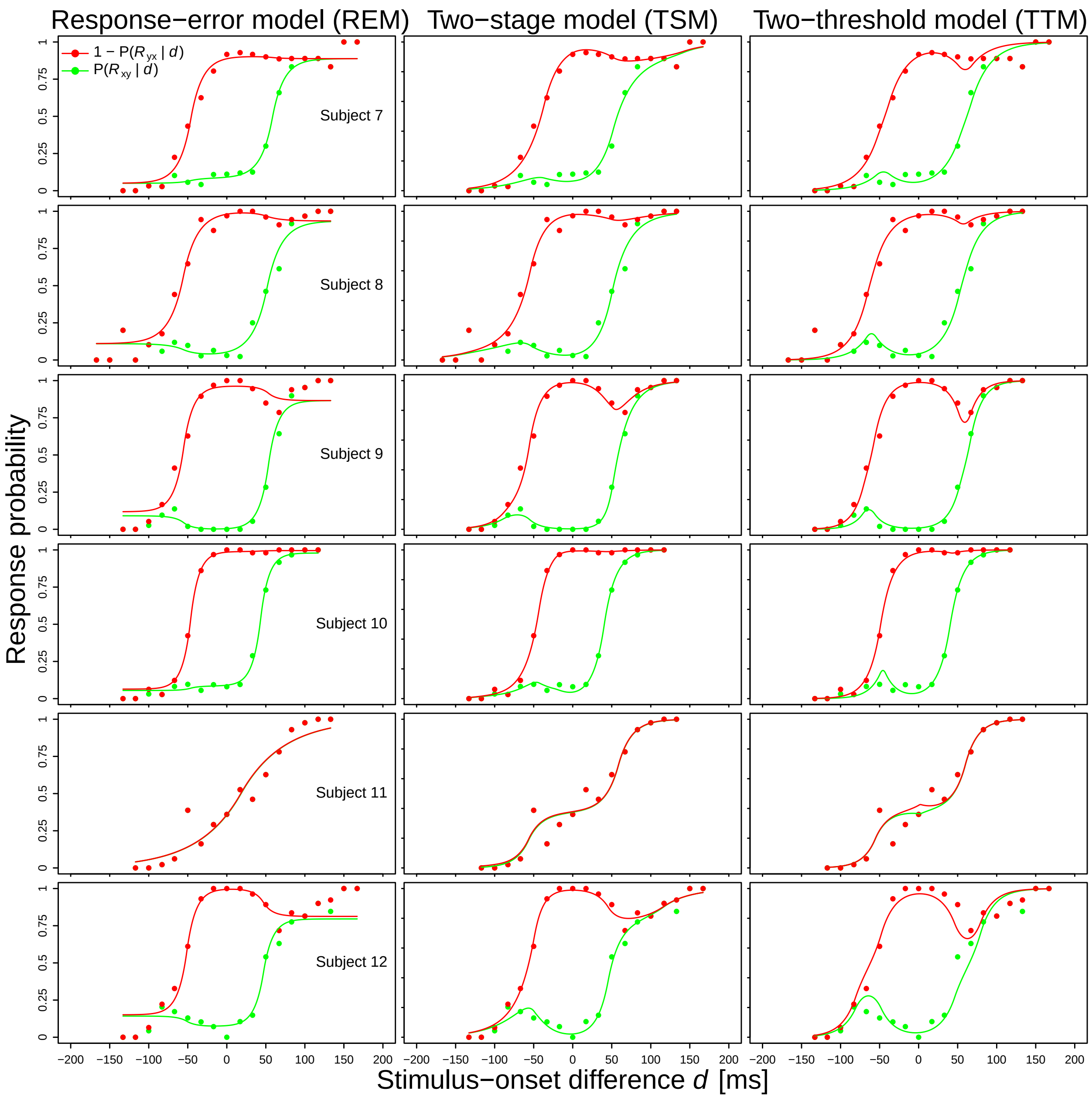

Note. x: right; y: left.

Figure S53

Observed (points) and fitted (lines) psychometric functions $P(R_{xy}\,|\,d)$ and $1 - P(R_{yx}\,|\,d)$ for subjects 13–19 in the study by García-Pérez and Alcalá-Quintana (2018)

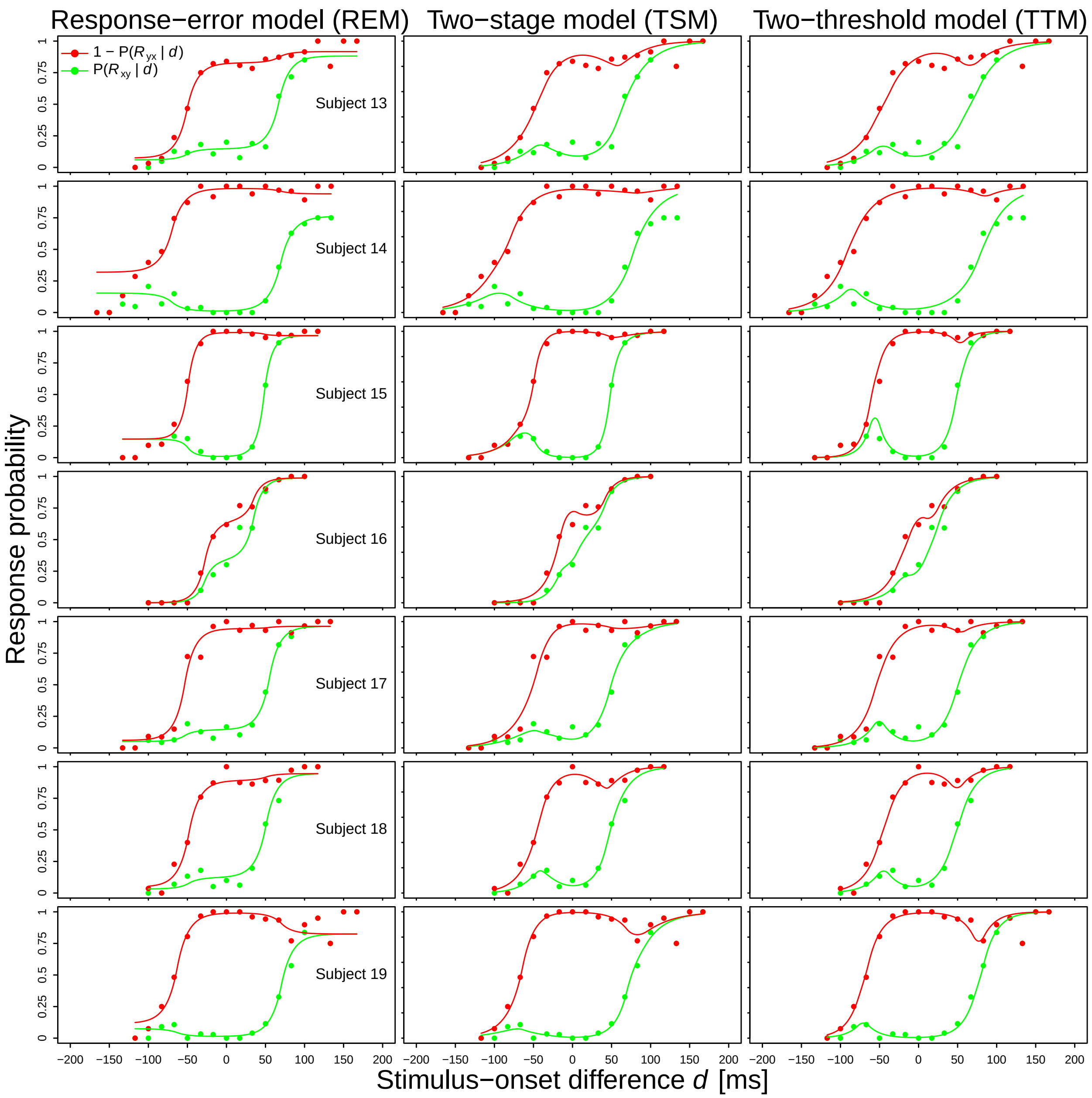

Note. x: right; y: left.

Figure S54

Observed (points) and fitted (lines) psychometric functions $P(R_{xy} \mid d)$ and $1 - P(R_{yx} \mid d)$ for the study by Lahkar et al. (2023)

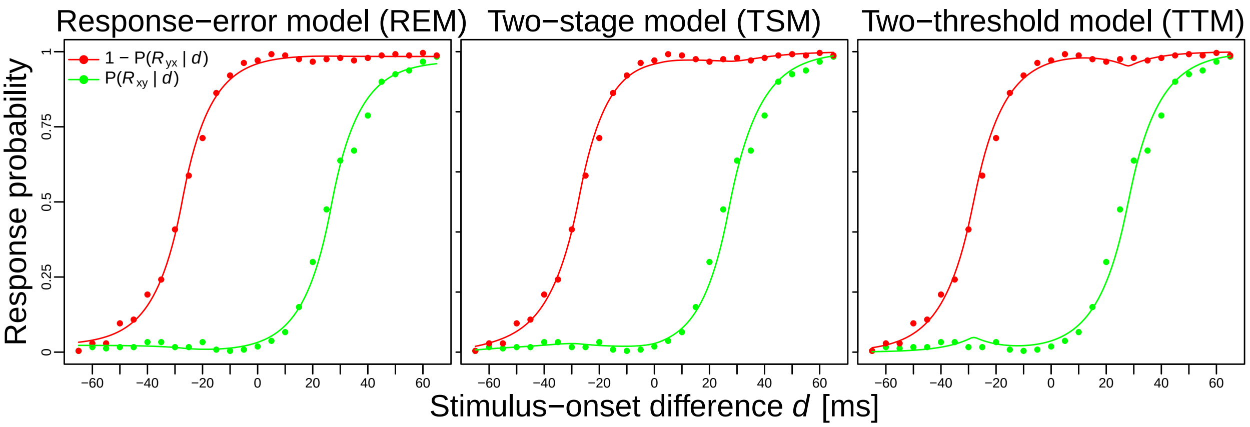

Note. x: right; y: left.

Tabular representation of other model comparison results

Parameter values for models with Laplace-distributed arrival-latency differences

Table S21

Mean best-fitting parameter values of the REM assuming a Laplace distribution of the arrival-latency difference across subjects and conditions within and across studies

Study	$\mu_{\mathbf{\Delta L}}$	$b_{\mathbf{\Delta L}}$	c	ϵ_{xy}	ϵ_{si}	ϵ_{yx}	κ_{xy-yx}	κ_{si-xy}	κ_{yx-xy}
Benussi (1913)	3	22	41	0.10	0.28	0.09	0.78	0.35	0.90
Allan (1975a)	−13	25	53	0.09	0.30	0.12	0.94	0.52	0.79
Allan (1975b)	−12	22	61	0.19	0.33	0.13	0.37	0.72	0.34
Ulrich (1987)	−2	16	49	0.16	0.37	0.05	0.94	0.47	0.93
Jaśkowski (1991a, Experiment 3)	−1	8	30	0.12	0.21	0.07	0.98	0.57	0.76
van Eijk et al. (2008)	−25	31	112	0.05	0.09	0.05	0.50	0.61	0.58
García-Pérez and Alcalá-Quintana (2018)	−3	14	50	0.07	0.16	0.08	0.74	0.56	0.68
Lahkar et al. (2023)	0	10	27	0.03	0.01	0.02	0.53	0.01	1.00
M	−7	18	53	0.10	0.22	0.08	0.72	0.48	0.75

Table S22

Mean best-fitting parameter values of the TSM assuming a Laplace distribution of the arrival-latency differences across subjects and conditions within and across studies

Study	$\mu_{\mathbf{\Delta L}_{su}}$	$\mu_{\mathbf{\Delta L}_{o}}$	$b_{\mathbf{\Delta L}_{su}}$	$b_{\mathbf{\Delta L}_{o}}$	c_{su}	c_o	g
Benussi (1913)	2	−3	29	41	31	60	0.51
Allan (1975a)	−13	−5	39	50	39	38	0.44
Allan (1975b)	−15	−28	52	34	54	61	0.78
Ulrich (1987)	−4	−17	29	32	31	50	0.53
Jaśkowski (1991a, Experiment 3)	−1	−15	12	14	25	43	0.63
van Eijk et al. (2008)	−27	−10	42	52	108	57	0.59
García-Pérez and Alcalá-Quintana (2018)	4	4	21	22	46	49	0.48
Lahkar et al. (2023)	0	28	14	17	27	31	0.02
M	−7	−6	30	33	45	49	0.50

Table S23

Mean best-fitting parameter values of the TTM assuming a Laplace distribution of the arrival-latency difference across subjects and conditions within and across studies

Study	$\mu_{\mathbf{\Delta L}}$	$b_{\mathbf{\Delta L}}$	c_{su}	c_o	g
Benussi (1913)	2	31	32	56	0.45
Allan (1975a)	−13	40	40	66	0.55
Allan (1975b)	−14	51	53	74	0.81
Ulrich (1987)	−4	29	31	62	0.43
Jaśkowski (1991a, Experiment 3)	−1	13	25	41	0.51
van Eijk et al. (2008)	−27	44	108	127	0.56
García-Pérez and Alcalá-Quintana (2018)	−2	17	46	62	0.57
Lahkar et al. (2023)	0	10	27	29	0.51
M	−7	29	45	65	0.55

Selection among models with different response-error and -bias parameters

Table S24

Sum of BIC values for a hierarchy of REMs across subjects and conditions within and across studies

Study	$\text{REM}_{\emptyset}$	REM_{xy}	REM_{si}	REM_{yx}	$\text{REM}_{xy,si}$	$\text{REM}_{xy,yx}$	$\text{REM}_{si,yx}$	REM	REM_{min}
Benussi (1913)	681.4	544.8	376.2	583.6	405.1	480.0	661.4	242.4	242.4
Allan (1975a)	1351.4	1159.1	517.0	1043.3	724.1	604.0	720.4	232.1	232.1
Allan (1975b)	936.8	845.3	372.5	787.1	532.6	924.9	804.1	452.5	372.5
Ulrich (1987)	1449.7	922.9	638.9	1320.8	820.6	902.9	1210.8	504.4	504.4
Jaśkowski (1991a, Experiment 3)	3442.4	2722.6	1936.8	2958.1	1668.6	2147.1	2817.7	1236.5	1236.5
van Eijk et al. (2008)	3505.2	3050.2	2731.7	2695.3	4003.6	5253.2	5752.5	2061.2	2061.2
García-Pérez and Alcalá-Quintana (2018)	3320.3	2892.5	1912.2	2572.1	2414.8	2135.7	2504.2	1535.0	1535.0
Lahkar et al. (2023)	640.3	420.0	278.6	397.1	931.1	872.4	969.2	155.4	155.4
$\sum$	15327.6	12557.4	8763.8	12357.3	11500.4	13320.1	15440.2	6419.4	6339.5

Non-monotonicity indices of observed and fitted psychometric functions

Table S25

Non-monotonicity index (NMI) of the data and the fits of the models assuming arrival-latency differences to be normally distributed (index N) or Laplace-distributed (index L) across subjects and conditions within and across studies

Study	Data	REM_N	TSM_N	TTM_N	REM_L	TSM_L	TTM_L
Benussi (1913)	0.096	0.000	0.000	0.000	0.000	0.042	0.074
Allan (1975a)	0.150	0.000	0.031	0.021	0.003	0.046	0.084
Allan (1975b)	0.233	0.015	0.076	0.110	0.025	0.057	0.160
Ulrich (1987)	0.500	0.038	0.395	0.413	0.044	0.493	0.703
Jaśkowski (1991a, Experiment 3)	3.305	0.748	2.027	2.057	0.780	2.121	2.523
van Eijk et al. (2008)	3.253	0.496	1.716	1.443	0.504	1.392	1.977
García-Pérez and Alcalá-Quintana (2018)	8.090	1.429	2.874	3.135	1.420	2.695	3.699
Lahkar et al. (2023)	0.096	0.010	0.034	0.031	0.014	0.012	0.041
$\sum$	15.723	2.736	7.153	7.210	2.790	6.858	9.261

Descriptions of the experiments

Allan, L. G. (1975a). The relationship between judgments of successiveness and judgments of order. *Perception & Psychophysics*, *18*, 29–36. https://doi.org/10.3758/BF03199363

Allan, L. G. (1975b). Temporal order psychometric functions based on confidence-rating data. *Perception & Psychophysics*, *18*, 369–372. https://doi.org/10.3758/BF03211214

Benussi, V. (1913). *Psychologie der Zeitauffassung [Psychology of time conception]*. Carl Winter's Universitätshandlung.

García-Pérez, M. A., & Alcalá-Quintana, R. (2018). Perceived temporal order and simultaneity: Beyond psychometric functions. In A. Vatakis, F. Balcı, M. Di Luca, & Á. Correa (Eds.), *Timing and time perception: Procedures, measures, and applications* (pp. 263–294). Brill. https://doi.org/10.1163/9789004280205_013

Jaśkowski, P. (1991a). Perceived onset simultaneity of stimuli with unequal durations. *Perception*, *20*, 715–726. https://doi.org/10.1068/p200715

Lahkar, R., Goyal, M., Mishra, P., Rao, B. N., Singh, Y., & Chowdhury, N. (2023). Insights into the perceptual moment theory: Experimental evidence from simultaneity judgment. *Attention, Perception, & Psychophysics*, *85*, 1199–1206. https://doi.org/10.3758/s13414-023-02684-7

Ulrich, R. (1987). Threshold models of temporal-order judgments evaluated by a ternary response task. *Perception & Psychophysics*, *42*, 224–239. https://doi.org/10.3758/BF03203074

van Eijk, R. L., Kohlrausch, A., Juola, J. F., & Van De Par, S. (2008). Audiovisual synchrony and temporal order judgments: Effects of experimental method and stimulus type. *Perception & Psychophysics*, *70*, 955–968. https://doi.org/10.3758/PP.70.6.955
